# ZC3H14 phosphorylation is required for nuclear speckle assembly during zygotic splicing activation in mice

**DOI:** 10.1038/s44319-026-00950-x

**Published:** 2026-10-07

**Authors:** Yang Wang, Hua Zhang, Zhe-Wei Hu, Dan Su, Jing Zhao, Xiao-Ying Fan, Li-Ling Liu, Heng-Yu Fan

**Affiliations:** 1 Life Sciences Institute, Zhejiang University, Hangzhou, 310058 China; 2 Zhejiang Key Laboratory of Precise Protection and Promotion of Fertility, Department of Obstetrics and Gynecology, Sir Run Run Shaw Hospital, School of Medicine, Zhejiang University, Hangzhou, 310016 China; 3 No. 9 Guangzhou National Laboratory, XingDaoHuanBei Road, Guangzhou International Bio Island, Guangzhou, 510005 Guangdong Province China; 4 Department of Reproductive Medicine and Genetics Center, The People’s Hospital of Guangxi Zhuang Autonomous Region, Nanning, 530021 China; 5 GMU-GIBH Joint School of Life Sciences, The Guangdong-Hong Kong-Macau Joint Laboratory for Cell Fate Regulation and Diseases, Guangzhou Medical University, Guangzhou, 510006 China

**Keywords:** Development, RNA Biology

## Abstract

Following zygotic genome activation (ZGA) in embryos, nuclear speckles, which are subnuclear domains where pre-mRNA undergoes splicing, are reassembled in a process called zygotic splicing activation (ZSA). ZSA is vital for early development, but its regulation is poorly understood. This study identifies the RNA-binding protein ZC3H14 as a key promoter of nuclear speckle formation during ZSA. Although viable, Zc3h14 knockout mice exhibit reduced female fertility. Most embryos from these females arrest during the 1-cell to 2-cell transition, and the remaining embryos arrest at the 2-cell stage, highlighting a critical role for maternally deposited ZC3H14. The protein localizes to nuclear speckles, and its phosphorylation by CDK12/13 regulates mRNA binding and initial speckles assembly. Blocking this phosphorylation disrupts proper splicing and leads to abnormal RNA accumulation. Consequently, phosphorylation-mimicking or phosphorylation-blocking ZC3H14 mutants expressed in zygotes positively and negatively affect development, respectively. Taken together, our results indicate that ZC3H14 phosphorylation by CDK12/13 maintains fertility in female mice by promoting nuclear speckle formation during ZSA.

## Synopsis

ZC3H14 phosphorylation by CDK12/13 is required for nuclear speckle reassembly during zygotic splicing activation in mice; its loss disrupts splicing, causes ptRNA accumulation, and leads to embryonic arrest at the 2-cell stage and female infertility.

- ZC3H14 phosphorylation by CDK12/13 is required for phosphorylation-dependent nuclear speckle assembly and proper splicing during zygotic splicing activation (ZSA).
- ZC3H14 dysfunction reduces alternative splicing, increases ptRNA accumulation, and impairs totipotency-to-pluripotency transition.
- ZC3H14 knockout causes female infertility with embryonic arrest at the 2-cell stage, revealing a maternal-effect requirement for ZC3H14 during ZSA.

## Abstract (web summary)

ZC3H14 phosphorylation by CDK12/13 is required for nuclear speckle reassembly during zygotic splicing activation in mice; its loss disrupts splicing, causes ptRNA accumulation, and leads to embryonic arrest at the 2-cell stage and female infertility.

## Graphical abstract

**Figure:**
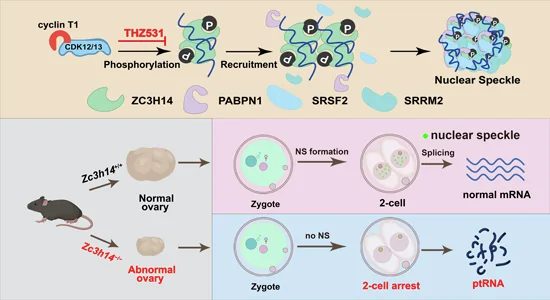

## Introduction

In mammals, zygotic development is initially controlled by maternal-derived materials from the oocyte, including mRNAs and proteins, at which point the zygotic genome is quiescent (Schultz, 1993; Telford et al, 1990). Subsequently, the maternal material is cleared and the zygotic genome is activated (ZGA; also referred to as embryonic genome activation, EGA, in some contexts), marking the embryo’s transition from “maternal control” to “embryonic control,” a developmental process known as maternal-to-zygotic transition (MZT) (Li et al, 2013; Tadros and Lipshitz, 2009). Recent studies have indicated that embryonic genome activation initiates as early as the one-cell stage across mammalian species, marking the onset of minor ZGA prior to the major wave of activation (Asami et al, 2022, 2023; Jin et al, 2026). However, the timing of major ZGA/EGA varies across species: in mice, it occurs at the 2-cell stage (Zeng et al, 2004; Wang et al, 2004; Xue et al, 2013), whereas in humans and cattle, it occurs at the 4- to 8-cell stage (Jin et al, 2026). This study focuses on the mouse model, in which major ZGA takes place at the 2-cell stage, when the transcriptional activity of the embryonic genome is significantly increased, resulting in a large number of zygotic transcripts that need to be spliced, capped, and tailed. Active splicing events occur during this process, and the expression of zygotic splicing factors undergoes drastic changes known as zygotic splicing activation (ZSA) (Zhang et al, 2024a), which ensures that transcripts are properly matured and transported to the cytoplasm for translation into function-specific proteins (Kalsotra and Cooper, 2011; Baralle and Giudice, 2017). Errors in the splicing process can lead to aberrant mRNA products that may not be properly transcribed into proteins or may accumulate as harmful substances within the cell (Wang et al, 2004).

Specifically, aberrantly spliced products can negatively affect normal embryonic development, leading to developmental disorders and even embryonic death (Shen et al, 2021). For example, SF3B1 is an important splicing factor phosphorylated during spliceosome activation (Wu et al, 2018; Hluchý et al, 2022). The small molecule pladienolide B (PlaB) inhibits splicing factor 3 B subunit 1 (SF3B1), which causes ZSA failure and affects the conversion of totipotency to pluripotency in early mouse embryos (Zhang et al, 2024a). However, the roles of other splicing factors in this complex physiological process remain unclear.

The mRNA metabolism is regulated by a variety of RNA-binding proteins related to mRNA degradation, which play an indispensable role in diverse mRNA degradation pathways (Zhao et al, 2020; Contreras et al, 2023; Wu et al, 2025). Poly(A) tail exosome targeting (PAXT) is an mRNA degradation pathway that mainly targets long polyadenylated RNA, such as spliced SNHG transcripts and prematurely terminated RNAs (ptRNAs) (Ogami et al, 2017). Zinc finger C3H1 domain-containing protein (ZFC3H1), mRNA transporter 4 (MTR4), zinc finger CCCH-type containing 14 (ZC3H14), and poly(A)-binding protein nuclear 1 (PABPN1) form core components of PAXT (Meola et al, 2016). The nuclear exosome targeting (NEXT) complex is another RNA degradation pathway that plays a role in post-transcriptional regulation, targeting short-lived target RNAs, such as promoter upstream transcripts (PROMPT), enhancer RNAs (eRNAs), and long noncoding RNAs (lncRNAs), without the requirement for polyadenylation (Lubas et al, 2015, 2011). The nuclear exosome targeting (NEXT) complex consists of MTR4, the zinc finger containing protein ZCCHC8, and the RNA-binding protein RBM7 (Puno and Lima, 2022).

In recent years, many studies have shown that mRNA metabolic disorders are associated with various diseases. Owing to a mutation in cyclin-dependent kinase 13 (CDK13), the alternative splicing regulator ZC3H14 cannot be activated by phosphorylation, which hinders the degradation of ptRNAs by PAXT, resulting in the accumulation of multiple oncogenic proteins and the induction of melanoma in zebrafish (Insco et al, 2023). However, the physiological significance of ZC3H14 phosphorylation in mammalian embryonic development remains unclear.

ZC3H14 is an important RNA-binding protein that contains a Q-rich structure at the N-terminus with a nuclear localization signal (NLS) in the middle, which normally allows the ZC3H14 protein to enter the nucleus (Wigington et al, 2016). The C-terminus contains CCCH zinc fingers that are associated with poly(A) RNA binding. ZC3H14 is mainly localized to the nucleus and contains three isoforms of different lengths. ZC3H14 and SRSF2 are co-localized in the nuclear speckle (Leung et al, 2009), which is the site of mRNA processing and splicing. ZC3H14 also interacts with several splicing factors (Li et al, 2024). Furthermore, ZC3H14 is closely associated with mRNA nucleation and is involved in the transport of mature mRNA from the nucleus to the cytoplasm (Fasken et al, 2008). ZC3H14 also plays an important role in regulating the polyadenylation acid tail (poly(A)) length of mRNA (Kelly et al, 2010; Pak et al, 2011). ZC3H14 binds to EIBs and 3’ UTRs to promote circRNA formation by interacting with the spliceosome. *Zc3h14* knockout male mice exhibited disrupted spermatogenesis and reduced circRNA levels (Li et al, 2024). However, the role of *Zc3h14* in female mouse oocyte development requires further investigation.

In this study, we found that ZC3H14 localized to nuclear speckles during the transcriptionally active period of mouse oocytes and early embryos, and that its phosphorylation promoted the formation of nuclear speckles and recruited other components of the PAXT complex, such as PABPN1. The small-molecule inhibitor THZ531 prevented nuclear speckle assembly by inhibiting the phosphorylation of ZC3H14, resulting in the accumulation of a large number of ptRNAs in the nucleus, ultimately causing developmental arrest of zygotes. *Zc3h14* knockout also caused ZSA defects and female infertility in mice. These regulatory mechanisms of ZC3H14 phosphorylation are related to mRNA processing and are essential for mouse embryo development.

## Results

### Dynamic localization of ZC3H14 and its co-localization with mRNA post-transcriptional processing proteins

**Figure EV1:**
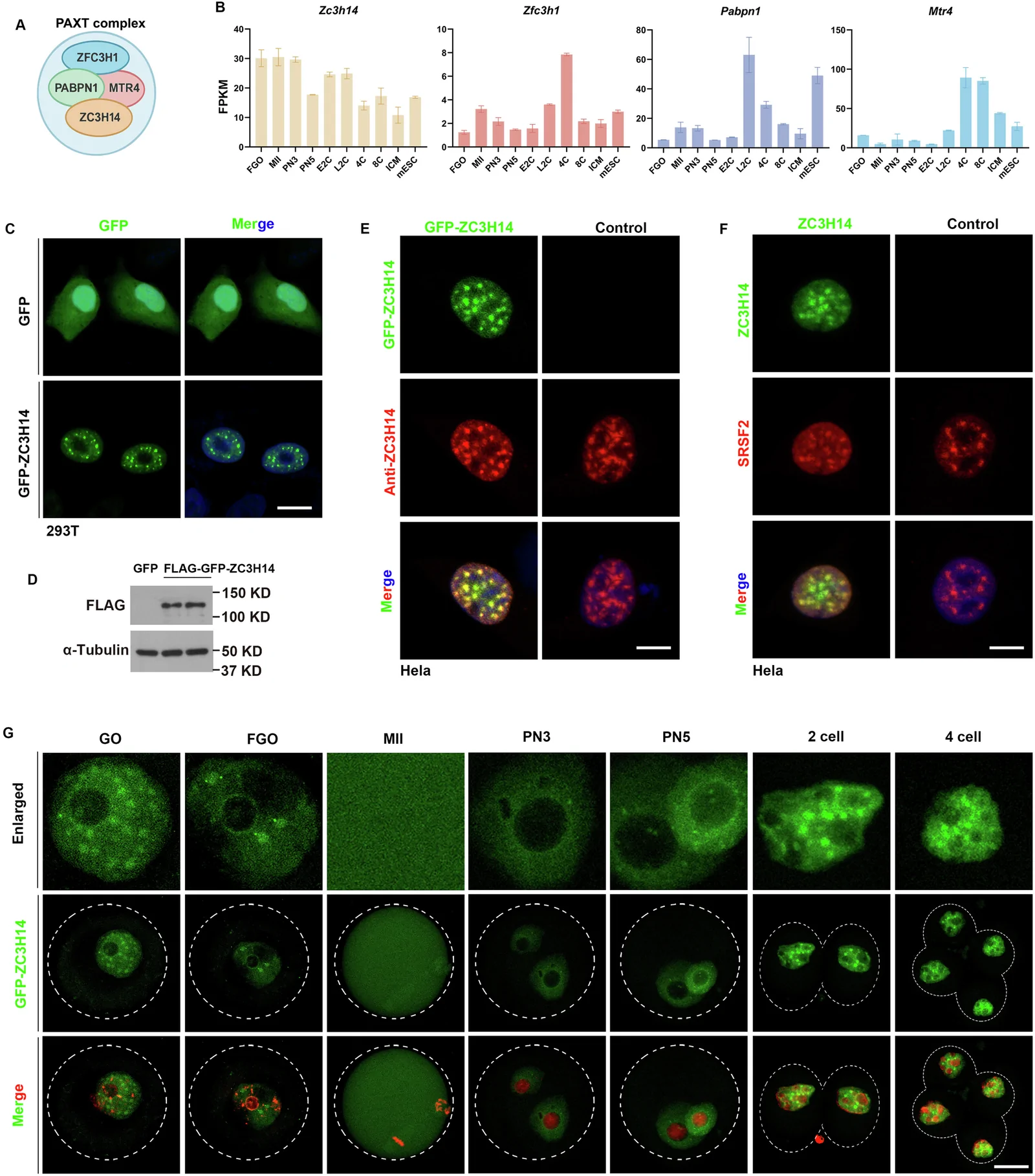
Localization and expression of ZC3H14 in oocytes and early mouse embryos. (**A**) Components of the PAXT complex include ZFC3H1, ZC3H14, PABPN1, and MTR4. (**B**) Expression level of PAXT components at different stages in mouse oocytes and early embryos. *n* = 3 biological replicates. Error bars, SEM. (**C**) Immunofluorescent staining showing the speckles of GFP-ZC3H14 in 293 T cells. Scale bars, 10 μm. (**D**) Western blotting results showing the expression of GFP-ZC3H14 in 293T. (**E**) Immunofluorescent staining showing the same speckles of GFP-ZC3H14 and endo-ZC3H14 in 293 T cells. Scale bars, 10 μm. (**F**) Immunofluorescent staining showing the co-localization of SRSF2 and endo-ZC3H14 in 293T cells. Scale bars, 10 μm. (**G**) Immunofluorescence staining showing the expression and localization of GFP-ZC3H14 in mouse oocytes and early embryos at different stages. Green, GFP-ZC3H14; red, DNA; The white dashed border highlights the membrane of the oocyte or embryo. scale bar, 20 μm.

The PAXT complex comprises four main components: ZFC3H1, PABPN1, MTR4, and ZC3H14 (Fig. EV1A). These components are highly expressed during the 2-cell and 4-cell stages of early embryonic development, suggesting that they may play critical roles during these stages. Among these, ZC3H14 was highly expressed at various stages of oocyte and early embryonic development (Fig. EV1B). We constructed a FLAG-GFP-ZC3H14 plasmid and transfected it into 293T cells. ZC3H14 was localized in the nucleus and formed speckle aggregates (Fig. EV1C). Western blotting (WB) analysis confirmed the expression of FLAG-GFP-ZC3H14 in these cells (Fig. EV1D). Immunofluorescence staining further validated the nuclear localization of ZC3H14, showing similar results in both GFP-ZC3H14-transfected and control cells (Fig. EV1E). The splicing factor SRSF2, a member of the SR protein family, is involved in alternative splicing and mRNA processing and is also a typical nuclear speckle protein (Lei et al, 2023; Gui et al, 1994; Fei et al, 2017; Kim et al, 2015). Immunofluorescent staining of Hela cells revealed complete co-localization of ZC3H14 and SRSF2 (Fig. EV1F). These results suggest that ZC3H14 is a component of the nuclear speckles in cells.

**Figure 1:**
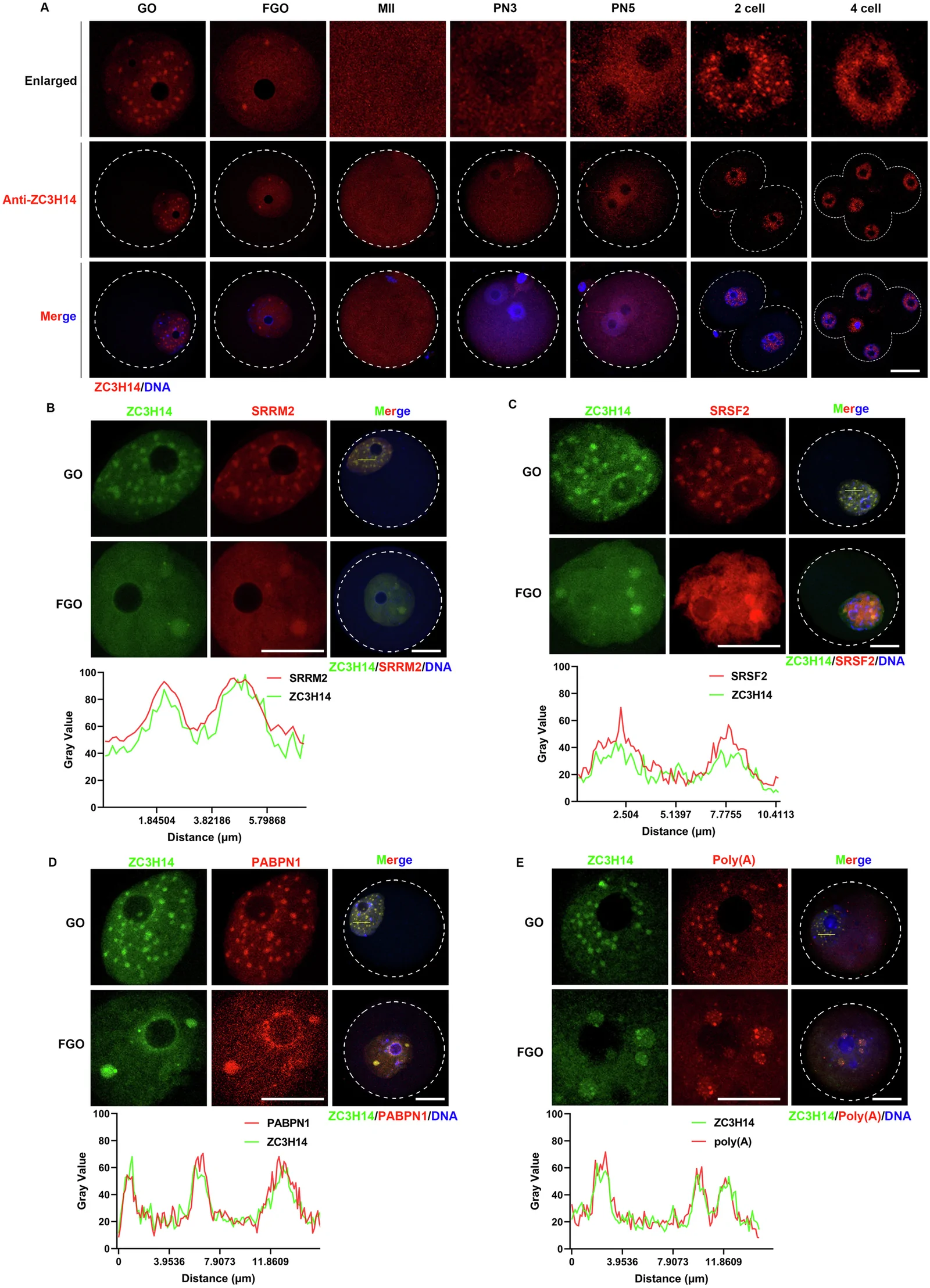
Dynamic localization changes of ZC3H14 and other proteins in mouse oocytes and early embryos. (**A**) Immunofluorescent staining showing the expression and localization of endogenous ZC3H14 in mouse oocytes and early embryos at different stages. The white dashed border highlights the membrane of the oocyte or embryo. Scale bars, 20 μm. (**B**) Immunofluorescent staining showing the localization of ZC3H14 and SRRM2 in a mouse growing oocyte (GO) and a fully grown oocyte (FGO). ImageJ was used to quantitatively analyze the distribution of ZC3H14 and SRRM2, indicated in the Figure by means of a yellow dotted line. The fluorescence intensity profiles used to qualitatively assess the fluorescence intensity changes of ZC3H14 and SRRM2 at the same position. The white dashed border highlights the membrane of the oocyte or embryo. Scale bars, 20 μm. (**C**) Immunofluorescent staining showing the localization of ZC3H14 and SRSF2 in GO and FGO. ImageJ was used to quantitatively analyze the distribution of ZC3H14 and SRSF2, indicated in the Figure by means of a yellow dotted line. The fluorescence intensity profiles used to qualitatively assess the fluorescence intensity changes of ZC3H14 and SRSF2 at the same position. The white dashed border highlights the membrane of the oocyte or embryo. Scale bars, 20 μm. (**D**) Immunofluorescent staining showing the localization of ZC3H14 and PABPN1 in GO and FGO. ImageJ was used to quantitatively analyze the distribution of ZC3H14 and PABPN1, indicated in the Figure by means of a yellow dotted line. The fluorescence intensity profiles used to qualitatively assess the fluorescence intensity changes of ZC3H14 and PABPN1 at the same position. The white dashed border highlights the membrane of the oocyte or embryo. Scale bars, 20 μm. (**E**) Immunofluorescent staining showing the localization of ZC3H14 and Poly(A) in GO and FGO. ImageJ was used to quantitatively analyze the distribution of ZC3H14 and Poly(A), indicated in the Figure by means of a yellow dotted line. The fluorescence intensity profiles used to qualitatively assess the fluorescence intensity changes of ZC3H14 and Poly(A) at the same position. The white dashed border highlights the membrane of the oocyte or embryo. Scale bars, 20 μm. Source data are available online for this figure.

We collected oocytes and embryos at different developmental stages and performed immunofluorescence staining using the ZC3H14 antibody, observing that endogenous ZC3H14 exhibits different localization patterns at different developmental stages. Notably, in the growing oocyte (GO), fully grown oocyte (FGO), and 2-cell stages, ZC3H14 formed prominent speckles in the nucleus. In fully grown oocytes, the number of speckles significantly decreased and became sparser than in other stages. In ovulated oocytes at metaphase II (MII), ZC3H14 showed a diffuse distribution. Notably, weak and sparse speckles began to appear at the PN3 and PN5 stage, coinciding with the onset of minor ZGA. The number and size of these speckles increased dramatically at the 2-cell stage when major ZGA occurs (Fig. 1A). The overexpression of GFP-ZC3H14 in oocytes and early embryos confirmed these observations (Fig. EV1G). Importantly, active transcription occurs during the GO and 2-cell stages, whereas transcription halts in the FGOs, suggesting that these localization changes may be directly related to its involvement in mRNA processing.

Serine/arginine repetitive matrix protein 2 (SRRM2) is a component of the spliceosome and plays a crucial role in the assembly of nuclear speckles (Ilik et al, 2020; Zhang et al, 2024b). Immunofluorescence revealed complete co-localization of ZC3H14, SRSF2, and SRRM2 during the FGO and GO stages, suggesting that ZC3H14 may play a role in mRNA splicing and nuclear speckle formation (Fig. 1B,C). PABPN1 is an RNA-binding protein that interacts with poly(A)-tailed mRNA through its RNA recognition motif (RRM) (Kühn and Wahle, 2004). The C-terminal (CCCH)_5_ domain of ZC3H14 functions as an RNA-binding domain. We observed that co-expressed ZC3H14 and PABPN1 co-localized in oocytes (Fig. 1D). Furthermore, immunofluorescence experiments showed that ZC3H14 co-localized with poly(A), further supporting the idea that ZC3H14 can bind poly(A)-tailed mRNAs (Fig. 1E).

### *Zc3h14* knockout leads to reduced fertility in mice

**Figure 2:**
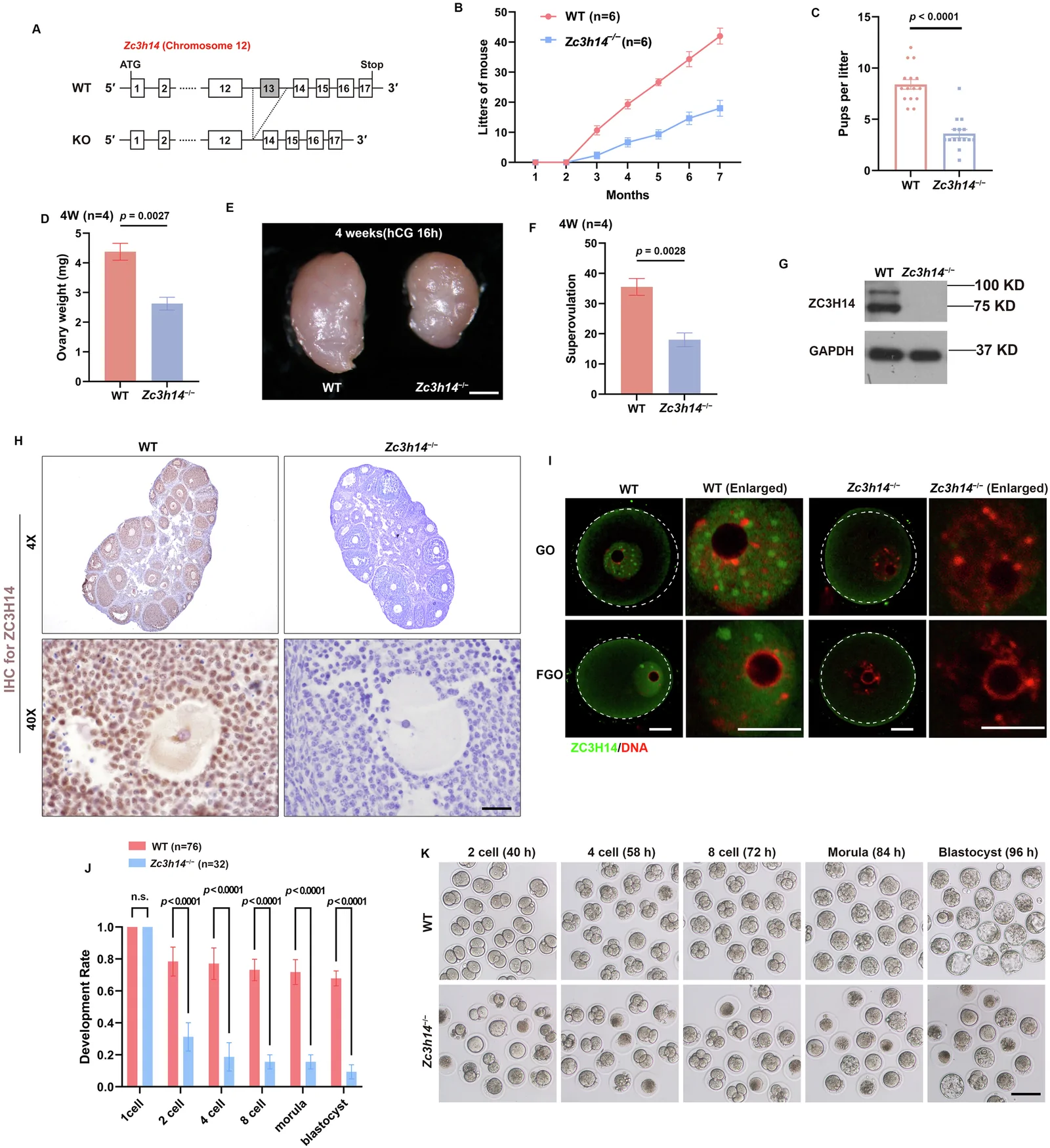
*Zc3h14*^*–/–*^ mice exhibit decreased fertility. (**A**) Diagram showing the strategy of *Zc3h14* knockout. (**B**) Fertility testing of WT and *Zc3h14*^*–/–*^ female mice. *n* = 6 females for each genotype. (**C**) Average number of pups per litter from WT and *Zc3h14*^*–/–*^ female mice. *n* = 6 females for each genotype. Error bars, SEM. Exact *P* values are indicated directly on the figure. Significance was determined by a two-tailed Student’s *t*-test. (**D**,** E**) Representative ovarian weights (**D**) and images (**E**) of WT and *Zc3h14*^*–/–*^ female mice at the 4-week-old. The experiment was independently repeated three times. 4W 4 weeks. Error bars, SEM. *P* = 0.0027 by two-tailed Student’s *t*-test. *n* number of mice analyzed for each genotype. Scale bar, 500 μm. (**F**) Number of oocytes ovulated by 4-week-old WT and *Zc3h14*^*–/–*^ mice 16 h after human chorionic gonadotropin (hCG) injection. Four female mice of each genotype (n) were used in this experiment. The experiment was independently repeated three times. Error bars, SEM. *P* = 0.0028 by two-tailed Student’s *t*-test. (**G**) Western blotting results showing ZC3H14 levels of WT and *Zc3h14*^*–/–*^ mice. GAPDH was blotted as a loading control. (**H**) Representative immunohistochemistry (IHC) staining images of ovaries from 4-week-old WT and *Zc3h14*^*–/–*^ mice. The 4× and 10× objectives were used. Scale bars, 50 μm. (**I**) Immunofluorescent staining showing the speckles of ZC3H14 in growing oocyte (GO) and fully grown oocyte (FGO) from WT and *Zc3h14*^*–/–*^ mice. The residual green signal in the knockout oocytes outlines the oocyte membrane/cytoplasm and represents background fluorescence, as confirmed by Western blot (**G**). The white dashed border highlights the membrane of the oocyte or embryo. Scale bars, 20 μm. (**J**) Preimplantation embryo development rates of 4-week-old WT and *Zc3h14*^*–/–*^ mice when embryos reach the corresponding stage. The experiment was independently repeated three times. The number of analyzed embryos are indicated (n). Error bars, SEM. Exact *P* values are indicated directly on the figure. Significance was determined by two-way analysis of variance (ANOVA) with a multiple comparisons test. n.s. not significant. (**K**) Representative images of preimplantation embryos at different stages that were ovulated by 4-week-old WT and *Zc3h14*^*–/–*^ mice. Scale bar, 100 μm. Time after human chorionic gonadotropin (hCG) injection is indicated (h). Source data are available online for this figure.

We constructed *Zc3h14* knockout (*Zc3h14*^*–/–*^) mice and found that they were viable. In fertility tests conducted over 7 months, the total number of pups and the average number of pups per litter in *Zc3h14*^*–/–*^ female mice were significantly reduced (Fig. 2A–C). The ovaries of 4-week-old wild-type (WT) and *Zc3h14*^*–/–*^ mice appeared similar in morphology, but those of *Zc3h14*^*–/–*^ mice were smaller than those of WT mice (Fig. 2D,E). After superovulation treatment, 4-week-old WT mice released an average of ~35 mature oocytes, whereas *Zc3h14*^*–/–*^ mice of the same age released significantly fewer oocytes (Fig. 2F). We confirmed knockout efficiency using WB and immunohistochemistry (IHC) (Fig. 2G,H). We performed immunofluorescence (IF) on oocytes from knockout mice and observed that ZC3H14 speckles completely disappeared (Fig. 2I).

After fertilization, we cultured oocytes in vitro and statistically analyzed their developmental stages at different time points. We found that most of the fertilized oocytes from WT mice developed normally to the blastocyst stage, whereas 70% of embryos from *Zc3h14*^*–/–*^ mice were arrested during the 1-cell to 2-cell transition and almost none developed to the blastocyst stage. Detailed stage-by-stage quantification revealed that while a small percentage of *Zc3h14*^*–/–*^ embryos could occasionally cleave to the 4-cell stage, they were morphologically abnormal and none progressed to the blastocyst stage (Fig. 2J,K). These results indicate that *Zc3h14* knockout significantly affected the reproductive capacity of female mice.

### Phosphorylation of ZC3H14 by CDK12/13 is essential for early mouse embryo development

**Figure 3:**
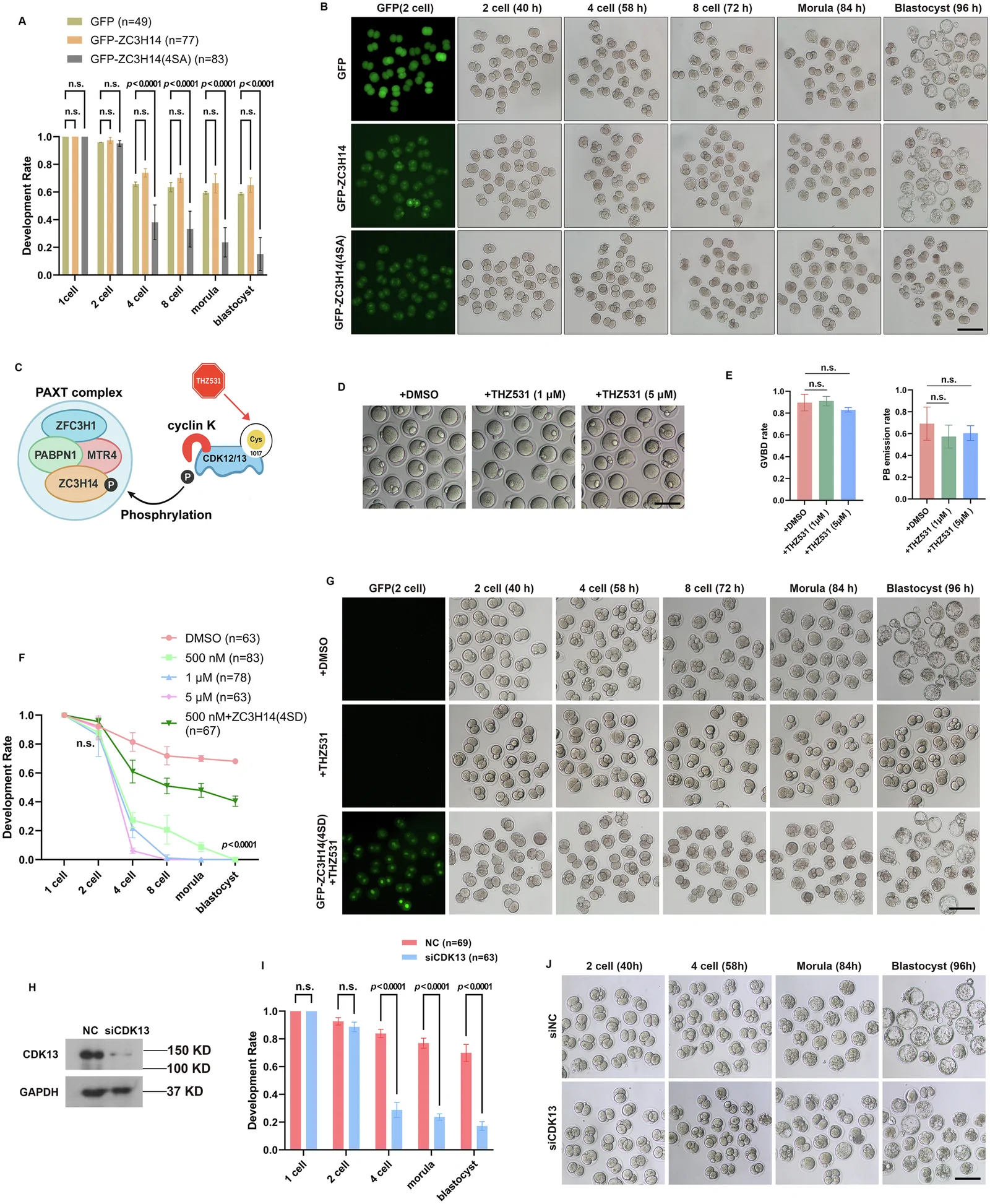
ZC3H14 phosphorylation is crucial for mouse early embryo development. (**A**) Development rates of preimplantation embryos after overexpression with GFP, GFP-ZC3H14, and GFP-ZC3H14 (4SA) when embryos reached corresponding stages. The experiment was independently repeated three times. The number of analyzed embryos are indicated (*n*). Error bars, SEM; n.s. nonsignificant. Exact *P* values are indicated directly on the figure. Significance was determined by two-way ANOVA with a multiple comparisons test. (**B**) Representative images of preimplantation embryos at different stages after overexpression with GFP-ZC3H14 and GFP-ZC3H14 phosphorylation site mutants (4SA). The mRNA injection experiments in (**A**, **B**) were performed in wild-type zygotes. Scale bar, 100 μm. Time after human chorionic gonadotropin (hCG) injection is indicated (h). (**C**) Illustration of CDK12/13 phosphorylating ZC3H14 and THZ531 blocking ZC3H14 phosphorylation. (**D**) Representative images of mouse MII oocytes after treatment with dimethyl sulfoxide (DMSO) or THZ531 (1 or 5 μM). Scale bar, 100 μm. (**E**) GVBD and PB emission rates of mouse oocytes after treatment with dimethyl sulfoxide (DMSO) or THZ531 (1 or 5 μM) when embryos reached corresponding stages. *n* = 3 biological replicates. Error bars, SEM. n.s. nonsignificant, by one-way ANOVA followed by Tukey’s test. (**F**) Development rates of early embryos after DMSO or THZ531 treatment or overexpression with GFP-ZC3H14 mimicking phosphorylated mutants (4 SD) at different time points. The experiment was independently repeated three times. The numbers of analyzed embryos are indicated (n). Error bars, SEM. n.s. nonsignificant. *P* values shown on the figure were determined by two-way analysis of variance (ANOVA) with a multiple comparisons test. (**G**) Representative images of early embryos at different stages after DMSO or THZ531 treatment or overexpression with GFP-ZC3H14(4 SD) during THZ531 treatment. Scale bar, 100 μm. (**H**) Western blotting results showing CDK13 protein levels of the NC and siCDK13 group. GAPDH was blotted as a loading control. (**I**) Development rates of early embryos after treatment with siRNA at different time points. The experiment was independently repeated three times. The numbers of analyzed embryos are indicated (n). Error bars, SEM. n.s. nonsignificant. *P* values shown on the figure were determined by two-way ANOVA with a multiple comparisons test. (**J**) Representative images of preimplantation embryos at different stages after treatment with siCDK13. Scale bar, 100 μm. Source data are available online for this figure.

ZC3H14 contains four major phosphorylation sites at serine residues 343, 409, 475, and 515 (Insco et al, 2023). We mutated these four serine residues to alanine to obtain the ZC3H14 (4SA) mutant to block phosphorylation, and all the serine residues were mutated to aspartic acid to obtain the ZC3H14 (4 SD) mutant to mimic phosphorylation. To evaluate the effects of phosphorylation mutations in ZC3H14 on early embryo development, we microinjected in vitro-transcribed mRNAs encoding GFP-ZC3H14 (4SA) into fertilized oocytes. The control group contained either GFP-ZC3H14 or GFP. The results showed that most fertilized oocytes from the control groups (GFP-ZC3H14 and GFP) developed to the blastocyst stage, whereas the GFP-ZC3H14 (4SA) mutation arrested most early embryos at the 2-cell stage and failed to develop into blastocysts (Fig. 3A,B).

We used THZ531, a small-molecule inhibitor that selectively binds to the C-terminal domain of CDK12/13 and inhibits its enzymatic function, thereby preventing CDK12/13 from phosphorylating ZC3H14 (Fig. 3C) (Zhang et al, 2016). In addition to the 2-cell stage, ZC3H14 exhibited the same speckle distribution at the GO stage (Fig. 1A). We investigated whether ZC3H14 plays a role in oocyte maturation. However, treatment with THZ531 did not significantly affect oocyte maturation, as there were no significant changes in germinal vesicle breakdown (GVBD) or polar body emission (PBE) levels (Fig. 3D,E), These results suggest that ZC3H14 is not essential for oocyte meiotic maturation under our experimental conditions.

**Figure EV2:**
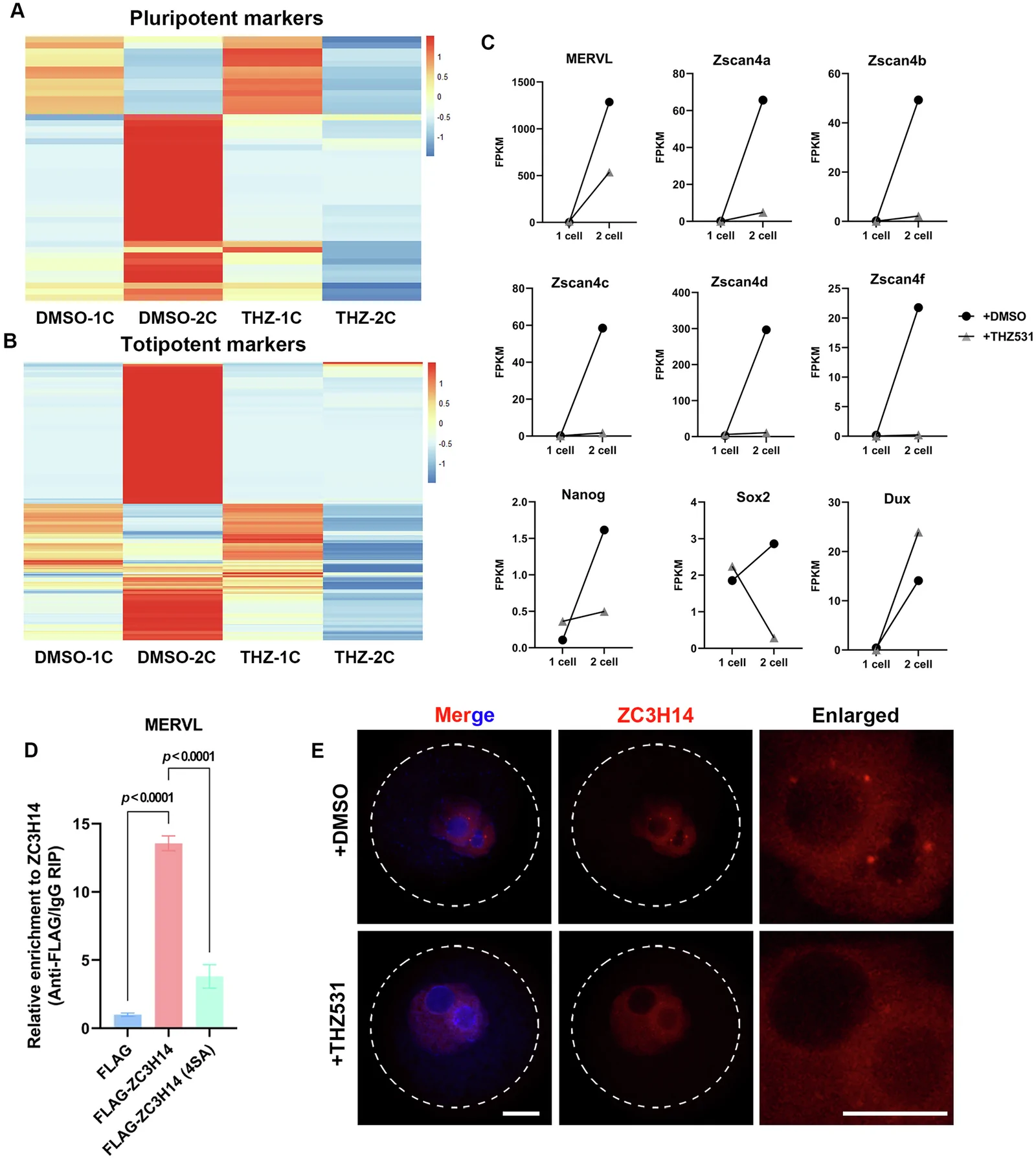
The effect of THZ531 on the expression levels of pluripotent and multipotent genes. (**A**) Heatmap shows the relative expression levels of representative pluripotent genes in 1-cell and 2-cell embryos treated with THZ531 or DMSO. (**B**) Heatmap shows the relative expression levels of representative totipotent genes in 1-cell and 2-cell embryos treated with THZ531 or DMSO. (**C**) The expression levels of some totipotent and pluripotent genes in 2-cell embryos treated with THZ531 or DMSO. (**D**) RNA immunoprecipitation (RIP) results showing the interaction of ZC3H14 with indicated transcripts. RNAs recovered from the immunoprecipitants were subjected to RT-qPCR of the indicated transcripts. The experiment was independently repeated three times. Error bars, SEM. *P* < 0.0001 by one-way ANOVA followed by Tukey’s test. (**E**) Immunofluorescent staining showing the speckles of ZC3H14 in 1-cell embryos after treatment with THZ531 or DMSO. The white dashed border highlights the membrane of the oocyte or embryo. Scale bar, 20 μm.

We then cultured fertilized oocytes in media containing 1 μM THZ531 and compared their developmental rates with the control group treated with an equivalent volume of DMSO. THZ531-treated zygotes were arrested at the 2-cell stage, whereas DMSO-treated zygotes developed normally into blastocysts, which is consistent with the results of the phosphorylation mutation. Furthermore, we replaced all four serine residues with aspartic acid residues (4 SD) to mimic the constitutive phosphorylation of ZC3H14. After treatment with THZ531, some fertilized eggs developed normally to the blastocyst stage (Fig. 3F,G), suggesting that the pharmacological effects of THZ531 were ZC3H14-specific. The 2-cell stage, a critical point in the transition from totipotency to pluripotency, showed altered expression of pluripotency markers in THZ531-treated embryos (Fig. EV2A,B; Dataset EV1). Compared to controls, THZ531 treatment leads to little change at the 1-cell stage, but downregulates key totipotency markers (e.g., *Zscan4* family, MERVL) and pluripotency markers (e.g., *Nanog*, *Sox2*) at the 2-cell stage. Interestingly, upstream activators like *Dux* show a trend towards upregulation, possibly a compensatory response to insufficient ZGA (Fig. EV2C). To directly test whether ZC3H14 binds MERVL or other repeat-derived transcripts, we performed RIP-qPCR experiments on lysates from wild-type 2-cell embryos using a ZC3H14 antibody. Our results show that ZC3H14 significantly enriches MERVL RNA compared to IgG control. Importantly, this enrichment is greatly diminished in the 4SA phosphorylation-deficient mutant, indicating that ZC3H14 phosphorylation is required for efficient binding to MERVL transcripts (Fig. EV2D). As a parallel validation of THZ531 pharmacological inhibition, we performed siRNA knockdown experiments targeting *Cdk13* (Fig. 3H). We tracked the development rate of embryos at different stages, and the results showed that most embryos were still arrested at the 2-cell stage (Fig. 3I,J), consistent with the phenotype seen with THZ531 treatment. These results demonstrate that the phosphorylation of ZC3H14 by CDK12/13 was critical for normal embryonic development.

### Phosphorylation of ZC3H14 promotes its own speckle formation

**Figure 4:**
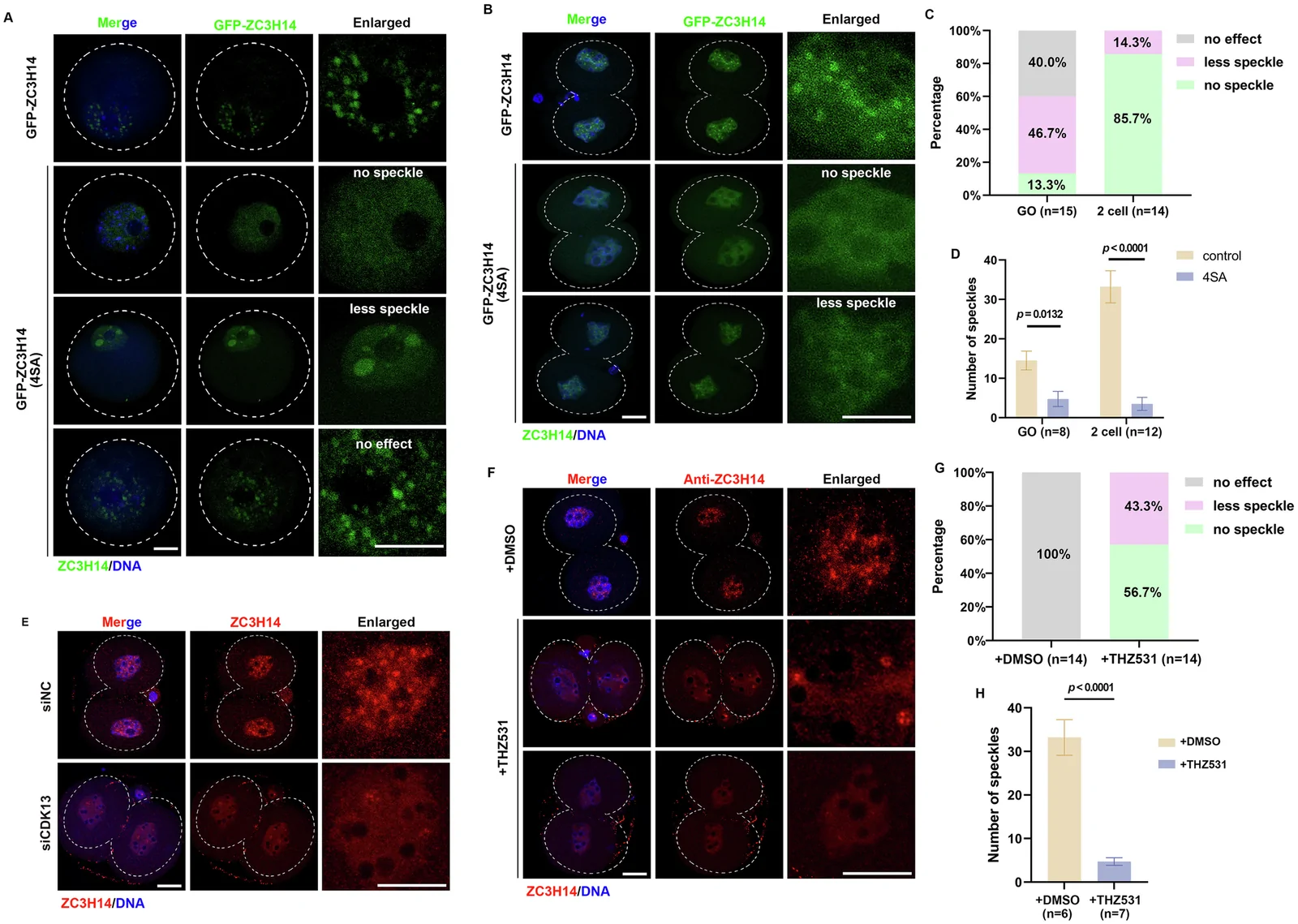
Phosphorylation of ZC3H14 promotes the formation of speckles. (**A**) Immunofluorescent staining showing the speckle changes of GFP-ZC3H14 and GFP-ZC3H14(4SA) in a mouse growing oocyte. The white dashed border highlights the membrane of the oocyte or embryo. Scale bar, 20 μm. (**B**) Immunofluorescent staining showing the speckle changes of GFP-ZC3H14 and GFP-ZC3H14(4SA) in 2-cell embryos. The white dashed border highlights the membrane of the oocyte or embryo. Scale bar, 20 μm. (**C**) Ratio of speckle changes in (**A**, **B**). The black numbers show the percentages of no effect, less speckle, and no speckle. (**D**) Quantification of speckles of GFP-ZC3H14 and GFP-ZC3H14(4SA) in GO and 2-cell embryos. The experiment was independently repeated three times. The numbers of analyzed embryos are indicated (n). Error bars, SEM. Exact *P* values are indicated directly on the figure. Significance was determined by two-way analysis of ANOVA with a multiple comparisons test. (**E**) Immunofluorescent staining showing the speckles of ZC3H14 in 2-cell embryos treated with siCDK13. The white dashed border highlights the membrane of the oocyte or embryo. Scale bar, 20 μm. (**F**) Immunofluorescent staining showing the speckles of ZC3H14 in 2-cell embryos after treatment with THZ531 (1 μM) or DMSO. The white dashed border highlights the membrane of the oocyte or embryo. Scale bar, 20 μm. (**G**) Ratio of speckle changes in (**E**). The black numbers show the percentages of no effect, fewer speckles, and no speckles. (**H**) Number of GFP-ZC3H14 speckles after treatment with THZ531 (1 μM) or DMSO in (**E**). The experiment was independently repeated three times. The number of analyzed embryos are indicated (n). Error bars, SEM. *P* < 0.0001 by two-tailed Student’s *t*-test. Source data are available online for this figure.

We microinjected the in vitro-transcribed mRNAs encoding GFP-ZC3H14 (4SA) into the GO and 2-cell stages. The 4SA mutation caused disappearance or reduction of speckles at the GO stage, although a few oocytes remained unaffected (Fig. 4A). Furthermore, the 4SA mutation significantly altered the localization of GFP-ZC3H14 in 2-cell embryos, with most speckles disappearing or decreasing in number, and the majority of GFP-ZC3H14 showing a diffuse distribution (Fig. 4B). We quantified the effects of the mutation on speckle number and localization during both stages and found that the 4SA mutation had a greater impact on GFP-ZC3H14 localization during the 2-cell stage (Fig. 4C,D). These results confirm that ZC3H14 phosphorylation was crucial for speckle formation at specific stages.

After knocking down CDK13, the number of ZC3H14 speckles in the two-cell stage was significantly reduced (Fig. 4E). Similarly, we used THZ531 to inhibit the enzymatic activity of CDK13. IF results showed that during the transition from the 1-cell to the 2-cell stage, THZ531 treatment also led to a reduction or complete disappearance of nuclear speckles in endogenous ZC3H14 cells compared to the control group. THZ531 treatment led to a reduction of ZC3H14 speckles in the nucleus of 1-cell stage embryos (Fig. EV2E), consistent with the effects observed at the 2-cell stage (Fig. 4F). This confirms that THZ531-mediated inhibition of ZC3H14 phosphorylation begins to affect speckle assembly as early as the 1-cell stage, prior to the major wave of speckle formation at the 2-cell stage. We also quantified the changes in speckle numbers at 2-cell stage and found that the proportion of cells with completely disappeared speckles was higher, which is consistent with the results of the 4SA mutation (Fig. 4G,H). Overall, THZ531 treatment significantly reduced the number of ZC3H14 speckles compared to those in the control group treated with DMSO.

**Figure EV3:**
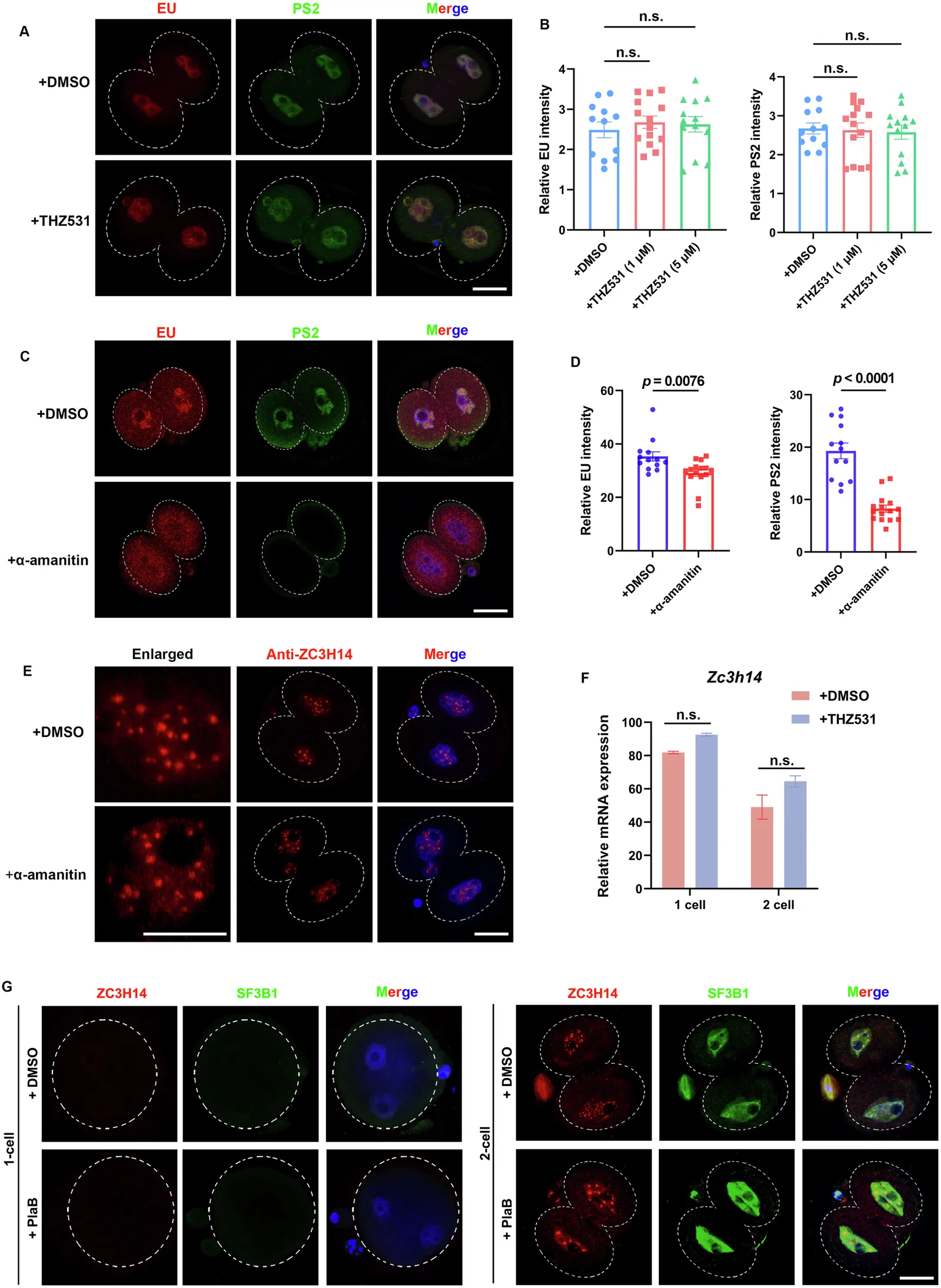
Speckle formation by ZC3H14 is not dependent on transcription and active splicing. (**A**) Immunofluorescence staining showing the fluorescence intensity of EU and pS2 in 2-cell embryos after treatment with THZ531 or DMSO. The white dashed border highlights the membrane of the oocyte or embryo. Scale bar, 20 μm. EU ethynyl uridine, pS2 phosphorylated RNA polymerase II. (**B**) ImageJ was used to quantitatively analyze the relative EU and pS2 intensity in (**A**). The number of analyzed embryos are indicated (n). Error bars, SEM. n.s. nonsignificant, by one-way ANOVA followed by Tukey’s test. (**C**) Immunofluorescence staining showing the fluorescence intensity of EU and pS2 in 2-cell embryos after treatment with α-amanitin (25 μM) or DMSO. The white dashed border highlights the membrane of the oocyte or embryo. Scale bar, 20 μm. (**D**) ImageJ was used to quantitatively analyze the relative EU and pS2 intensity in (**C**). The number of analyzed embryos are indicated (n). Error bars, SEM. Exact *P* values are indicated directly on the figure. Significance was determined by a two-tailed Student’s *t*-test. (**E**) Immunofluorescence staining showing the speckles of ZC3H14 in 2-cell embryos after treatment with α-amanitin (25 μM) or DMSO. The white dashed border highlights the membrane of the oocyte or embryo. Scale bar, 20 μm. (**F**) The relative RNA expression level of ZC3H14 in 1-cell and 2-cell embryos after treatment with THZ531 (1 μM) or DMSO. *n* = 3 biological replicates. Error bars, SEM. n.s. nonsignificant, by a two-tailed Student’s *t*-test. (**G**) Immunofluorescent staining showing the speckles of ZC3H14 and the expression level of SF3B1 in zygote and 2-cell embryos after treatment with PlaB (100 nM) or DMSO. The white dashed border highlights the membrane of the oocyte or embryo. Scale bar, 20 μm.

We examined whether THZ531 treatment affected ZC3H14 localization by monitoring its transcriptional activity. We detected global transcription activities in 2-cell embryos using a 5-ethynyl uridine (EU) incorporation assay and IF staining of RNA polymerase II phosphorylated at serine-2 (pS2) and found no significant difference between the treatment and control groups, suggesting that THZ531 did not affect early embryonic transcription at this concentration (Fig. EV3A,B). To further exclude transcriptional effects, we treated fertilized eggs with the small-molecule inhibitor α-amanitin, which effectively inhibits transcription at a concentration of 25 μg/μl (Fig. EV3C,D). IF results showed that α-amanitin treatment did not affect ZC3H14 localization (Fig. EV3E), indicating that ZC3H14-mediated nuclear speckle assembly does not depend on newly transcribed RNAs from zygotic genome activation. RNA-seq and RT-PCR analyses confirmed that THZ531 did not significantly affect the mRNA expression of ZC3H14 (Fig. EV3F). To test whether speckle formation is a consequence of global splicing activity, we treated 1-cell embryos with the splicing inhibitor PlaB. Notably, ZC3H14 nuclear speckles were still able to assemble at the 2-cell stage under PlaB treatment, although the number of speckles was reduced (Fig. EV3G), indicating that speckle assembly is not simply a downstream consequence of active splicing. These results suggest that changes in ZC3H14 speckles were not related to transcription or expression levels but rather to CDK12/13-mediated phosphorylation.

### ZC3H14 is crucial for the phosphorylation-dependent assembly of nuclear speckles during ZSA

**Figure 5:**
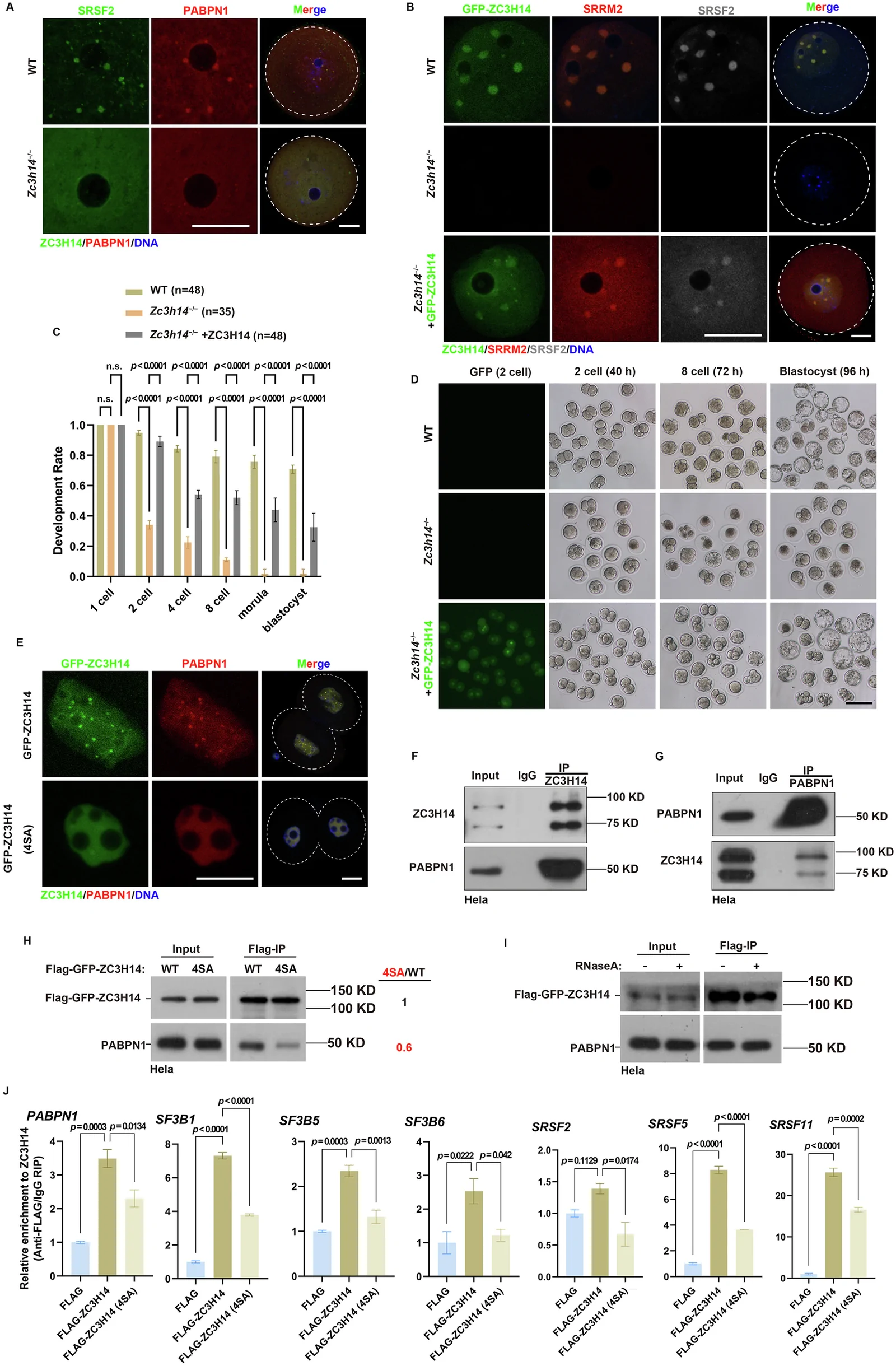
Phosphorylation of ZC3H14 promotes its binding to mRNA and the assembly of nuclear speckles. (**A**) Immunofluorescent staining showing the localization of SRSF2 and PABPN1 in a fully grown oocyte (FGO) from WT and *Zc3h14*^*–/–*^ mice. The white dashed border highlights the membrane of the oocyte or embryo. Scale bars, 20 μm. (**B**) Immunofluorescent staining showing the localization of SRSF2 and SRRM2 in oocyte from WT and *Zc3h14*^*–/–*^ mice, and *Zc3h14*^*–/–*^ oocyte after GFP-ZC3H14 microinjection. The white dashed border highlights the membrane of the oocyte or embryo. Scale bars, 20 μm. (**C**) Preimplantation embryo development rates of 4-week-old WT and *Zc3h14*^*–/–*^ mice when embryos reach the corresponding stage, and the development rate of *Zc3h14*^*–/–*^ zygote after GFP-ZC3H14 microinjection. The experiment was independently repeated three times. The number of analyzed embryos are indicated (n). Error bars, SEM. Exact *P* values are indicated directly on the figure. Significance was determined by two-way ANOVA with a multiple comparisons test. (**D**) Representative images of preimplantation embryos at different stages that were ovulated by 4-week-old WT and *Zc3h14*^*–/–*^ mice, and the image of *Zc3h14*^*–/–*^ zygote after GFP-ZC3H14 microinjection. Scale bar, 100 μm. Time after human chorionic gonadotropin (hCG) injection is indicated (h). (**E**) Immunofluorescent staining showing the speckles of GFP-ZC3H14 and PABPN1 after GFP-ZC3H14 or GFP-ZC3H14(4SA) microinjection in 2-cell embryos. The white dashed border highlights the membrane of the oocyte or embryo. Scale bar, 20 μm. (**F**,** G**) Immunoprecipitation (IP) showing the interaction of ZC3H14 with PABPN1. (**H**,** I**) Co-IP showing the interaction of PABPN1 with GFP-ZC3H14 or GFP-ZC3H14 (4SA), and the interaction of GFP-ZC3H14 with PABPN1 after treatment with RNase A. (**J**) RNA immunoprecipitation (RIP) results showing the interaction of ZC3H14 with indicated transcripts. RNAs recovered from the immunoprecipitants were subjected to RT-qPCR of the indicated transcripts. The experiment was independently repeated three times. Error bars, SEM. Exact *P* values are indicated directly on the figure. Significance was determined by one-way ANOVA followed by Tukey’s test. Source data are available online for this figure.

**Figure EV4:**
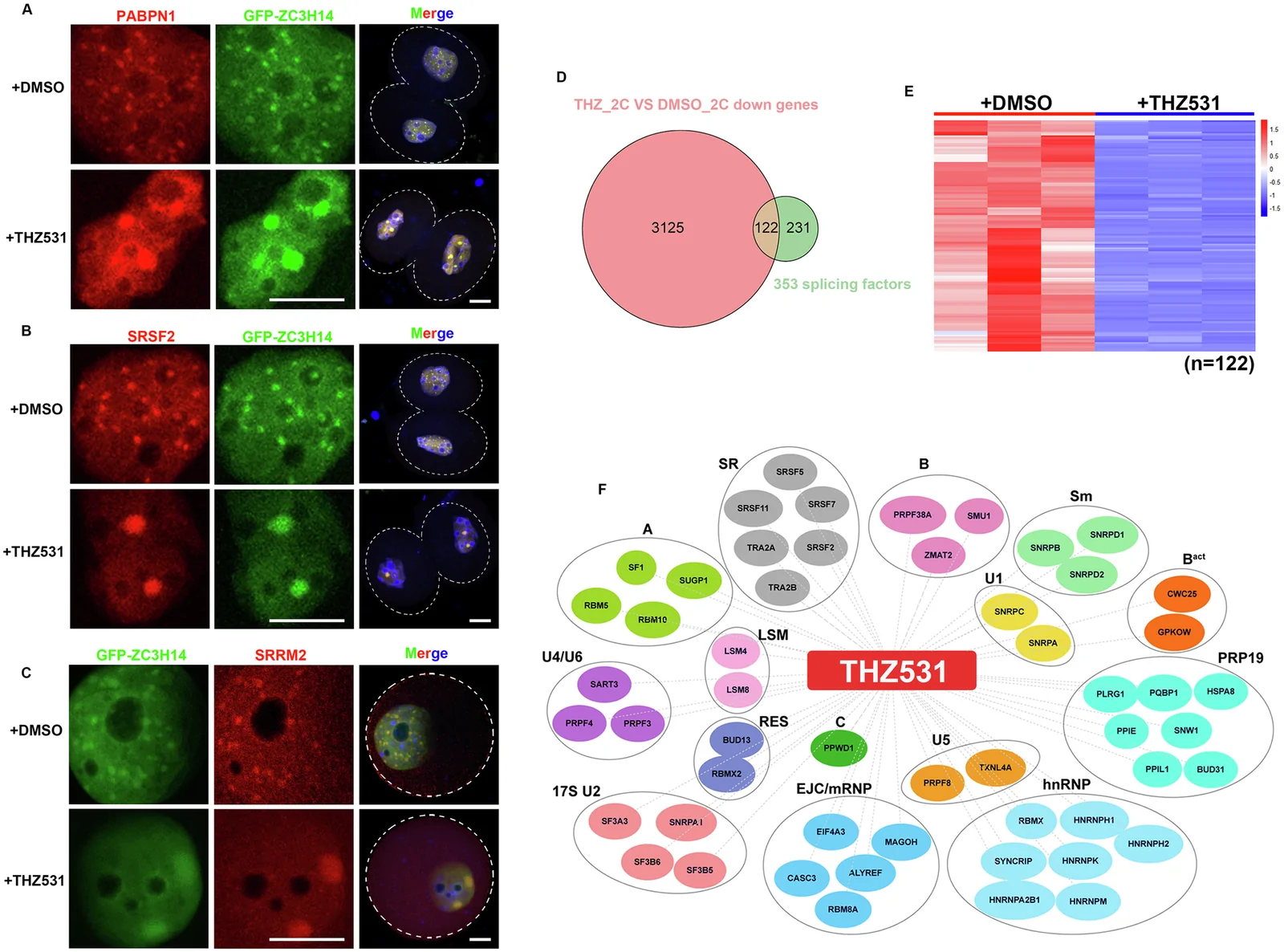
Effects of THZ531 on alternative splicing and formation of nuclear speckles. (**A**) Immunofluorescent staining showing the speckle scales of ZC3H14 and PABPN1 in 2-cell embryos after treatment with THZ531 (1 μM) or DMSO. The white dashed border highlights the membrane of the oocyte or embryo. Scale bar, 20 μm. (**B**) Immunofluorescent staining showing the speckle scales of ZC3H14 and SRSF2 in 2-cell embryos after treatment with THZ531 (1 μM) or DMSO. The white dashed border highlights the membrane of the oocyte or embryo. Scale bar, 20 μm. (**C**) Immunofluorescent staining showing the speckle scales of ZC3H14 and SRRM2 in growing oocyte after treatment with THZ531 (1 μM) or DMSO. The white dashed border highlights the membrane of the oocyte or embryo. Scale bar, 20 μm. (**D**) Venn diagrams showing the overlap of downregulated genes from THZ531 treatment and 353 splicing factors. (**E**) Heatmap showing the normalized expression levels of 122 splicing factors from (**B**) in 2-cell embryos treated with DMSO or THZ531. (**F**) Spliceosome components affected by THZ531 from RNA-seq data.

We performed IF on oocytes from *Zc3h14*^*–/–*^ mice and observed that the proteins SRSF2 and PABPN1, which were co-localized with ZC3H14, also disappeared (Fig. 5A). Overexpression of GFP-ZC3H14 in *Zc3h14*^*–/–*^ oocytes restored the SRRM2 and SRSF2 speckles (Fig. 5B). We found that most embryos from *Zc3h14*^*–/–*^ mice were arrested during the 1-cell to 2-cell transition, and overexpression of GFP-ZC3H14 significantly developed them into the blastocyst stage (Fig. 5C,D). Furthermore, we found that after treatment with THZ531, the number of speckles in the nucleus of ZC3H14 and three co-localized proteins, SRRM2, SRSF2, and PABPN1, significantly decreased, whereas the control group was treated with DMSO (Fig. EV4A–C). Our IF experiments also showed that the overexpression of GFP-ZC3H14 (4SA) in 2-cell embryos resulted in the disappearance of ZC3H14 and PABPN1 speckles, indicating that nuclear speckles failed to assemble more severely than THZ531 treatment (Fig. 5E). PABPN1 is a classical RNA-binding protein that is localized to nuclear speckles. Through immunoprecipitation (IP) experiments, we demonstrated that ZC3H14 and PABPN1 interacted directly (Fig. 5F,G), and the four phosphorylation site mutations in ZC3H14 affected their interaction, which was RNA-independent (Fig. 5H,I). RIP-qPCR experiments revealed that mutations in the phosphorylation sites of ZC3H14 weakened its interaction with some mRNAs, including those encoding PABPN1 and several splicing factors (Fig. 5J). These results suggest that inhibiting ZC3H14 phosphorylation may affect mRNA binding and the phosphorylation-dependent assembly of nuclear speckles during ZSA.

### THZ531 inhibits alternative splicing by suppressing ZC3H14 phosphorylation

**Figure 6:**
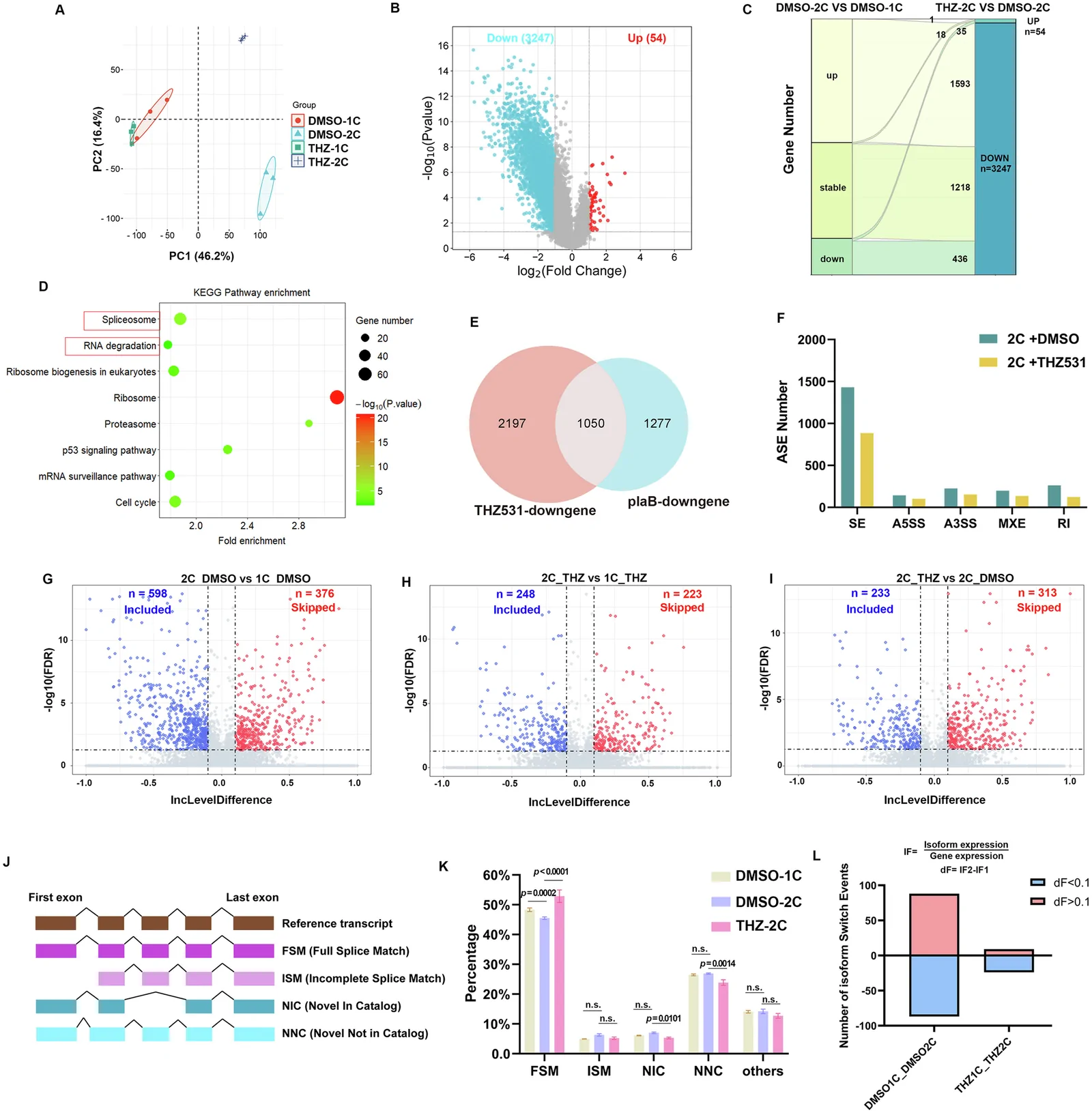
Alternative splicing events (ASEs) are reduced owing to THZ531 inhibition of ZC3H14 phosphorylation. (**A**) Transcriptome-based PCA of embryos treated with THZ531 or DMSO. 1C 1-cell, 2C 2-cell, THZ THZ531. (**B**) Differentially expressed gene (DEG) numbers in mouse early embryos at different stages. DEGs were identified using the limma package (moderated *t*-test) with Benjamini–Hochberg correction for multiple comparisons; significance was defined as FDR <0.05 and |log_2_(fold change)| >2 or <0.5. FPKM, fragments per kilobase of transcript per million mapped reads. (**C**) StreamGraph showing the sources of up- and downregulated genes. The black number represents the number of genes. (**D**) KEGG pathway analysis of 3318 downregulated genes with a bubble chart. KEGG pathway enrichment analysis was performed on the identified DEGs using the clusterProfiler package in R. The hypergeometric test was applied to assess the statistical significance of pathway enrichment. *P* values were corrected for multiple comparisons using the Benjamini–Hochberg method. Pathways with FDR <0.05 were considered significantly enriched. (**E**) Venn diagrams showing the overlap of downregulated genes from THZ531 and PlaB (100 nM) treatment. (**F**) Change in the numbers of five different ASEs in 2-cell embryos treated with THZ531 or DMSO.SE skipped exon, A5SS alternative 5’ splice site, A3SS alternative 3’ splice site, MXE mutually exclusive exon, RI retained intron. (**G**–**I**) ASE number of 2-cell embryos treated with DMSO or THZ531 are shown with a volcano map. ASEs were identified using the limma package (moderated *t*-test) with Benjamini–Hochberg correction for multiple comparisons; significance was defined as FDR <0.05 and |ILD|>0.1. FDR false discovery rate, ILD incLevelDifference. (**J**) Structure diagram showing splice matches of the different transcripts. FSM full splice match, ISM incomplete splice match, NIC novel in catalog, NNC novel not in catalog. (**K**) Ratio of different splice match transcripts after treatment with DMSO or THZ531 in mouse embryos at the 1- to 2-cell stage. *n* = 3 biological replicates. Error bars, SEM; n.s. nonsignificant. Exact *P* values are indicated directly on the figure. Significance was determined by two-way ANOVA with a multiple comparisons test. (**L**) Number of isoform switch events after treatment with DMSO or THZ531 in mouse embryos at the 1- to 2-cell stage. Source data are available online for this figure.

To investigate the impact of ZC3H14 inhibition on the transcriptome, we treated mouse fertilized eggs with 1 μM THZ531 and collected 1–2-cell embryos, using DMSO as a control for sequencing with Smart-seq2. Principal component analysis (PCA) revealed some separation between THZ531-treated and control embryos as early as the 1-cell stage; however, the transcriptional differences became much more pronounced and robust at the 2-cell stage (Fig. 6A). THZ531 was added at the early 1-cell stage, and it is plausible that it exerts a mild effect on the minor wave of zygotic transcription. However, the major transcriptional and splicing defects are clearly manifested at the 2-cell stage, coinciding with major ZGA when the demand on the splicing machinery is at its peak. A volcano plot showed that 3247 genes were downregulated and 54 genes were upregulated in THZ531-treated 2-cell embryos compared to the control (Fig. 6B; Dataset EV2). The downregulated genes mainly belonged to the original upregulated and stable genes during zygote-to-2-cell development (Fig. 6C). Gene Ontology (GO) and Kyoto Encyclopedia of Genes and Genomes (KEGG) pathway analyses of the downregulated genes revealed enrichment in RNA splicing and degradation, ribosome biogenesis, and p53 signaling pathways (Fig. 6D). Analyses of published RNA-seq data indicated that 2-cell mouse embryos treated with PlaB had 2327 downregulated genes, with 45.1% (1050) of these genes overlapping with those downregulated by THZ531 treatment, indicating that THZ531 treatment affected a significant portion of splicing-sensitive genes (Fig. 6E). Common alternative splicing events (ASEs) include intron retention (IR), exon skipping (SE), mutually exclusive exons (MXE), and alternative 5’ and 3’ splice sites (A5SS and A3SS, respectively). Analysis of the five different types of ASEs revealed that A5SS, A3SS, MXE, SE, and IR showed varying degrees of reduction after THZ531 treatment (Fig. 6F).

We used replicate multivariate analysis of transcript splicing (rMATs) (Shen et al, 2012, 2014) to analyze the changes in ASEs. Normally, 598 events were included, and 376 skipped events occurred during the 1–2-cell development process (Fig. 6G). After THZ531 treatment, the number of included events decreased to 248, and the number of skipped events decreased to 223 (Fig. 6H), indicating that THZ531 treatment reduced the overall number of splicing events. Separate analysis of the different splicing events that occurred during the 2-cell stage showed that compared to the control, THZ531-treated 2-cell embryos experienced 233 included events and 313 skipped events (Fig. 6I). This indicates that many abnormal splicing events occurred after treatment with THZ531. Additionally, of the 353 splicing factors in mice, 34.5% (122) showed downregulated expression after THZ531 treatment (Fig. EV4D,E; Dataset EV3). These splicing factors belong to different components of the spliceosome, including the SR protein family, Sm-like proteins, and U1, U2, U4/U6, and U5 ribonucleoproteins (Fig. EV4F).

**Figure EV5:**
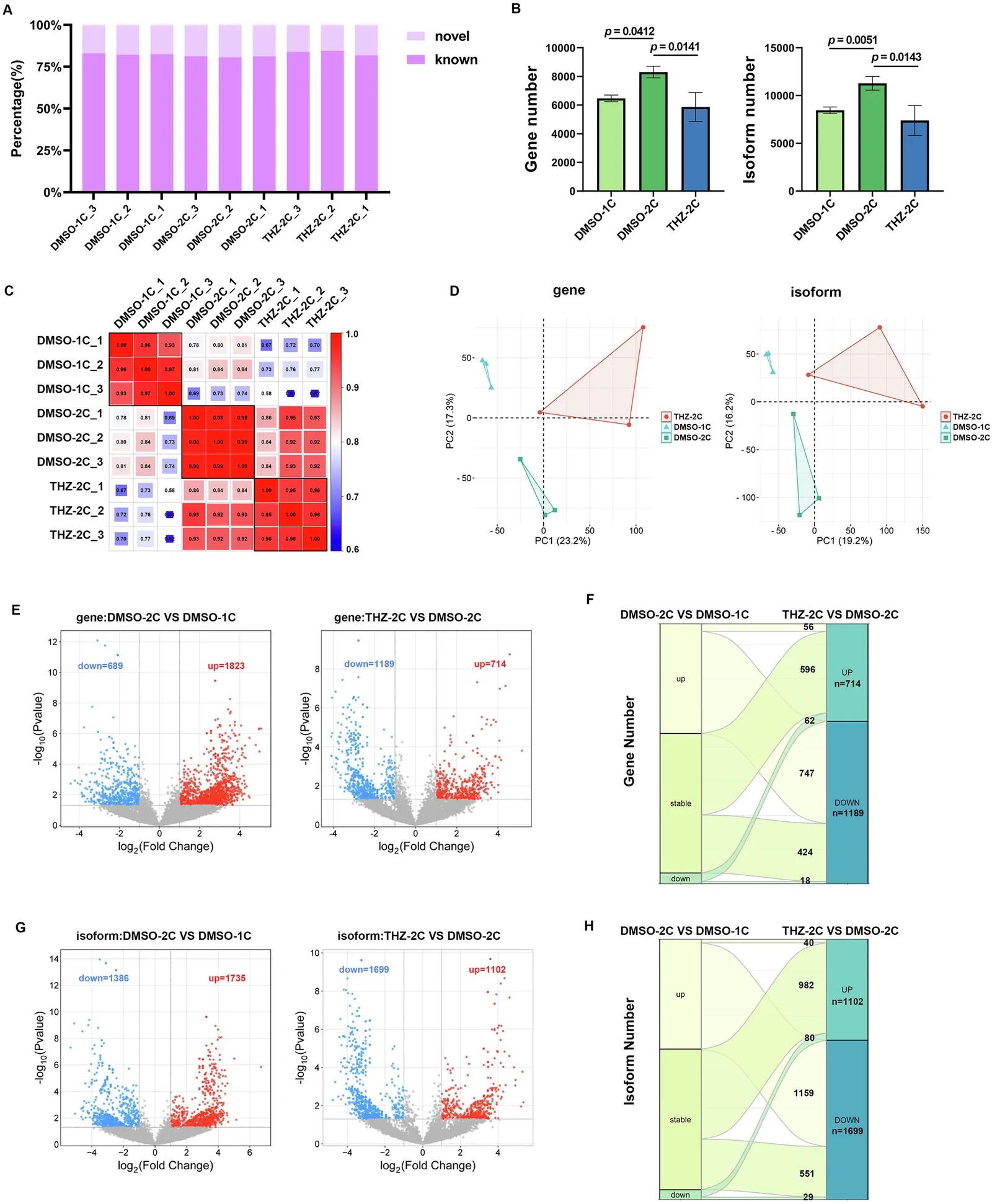
Full-length transcriptome sequence quality control and differentially expressed genes or isoforms. (**A**) Statistics on the proportion of known transcripts in each sample. (**B**) Gene or isoform number of 1–2-cell embryos treated with THZ531 or DMSO. *n* = 3 biological replicates. Error bars, SEM. Exact *P* values are indicated directly on the figure. Significance was determined by one-way ANOVA followed by Tukey’s test. (**C**) Analyses of the correlations between the indicated biological replicates of 1-cell and 2-cell embryos treated with DMSO or THZ531. (**D**) Transcriptome-based PCA of embryos treated with THZ531 or DMSO. (**E**) Differentially expressed gene (DEG) numbers in mouse early embryos at different stages. DEGs and isoforms (**G**) were identified using the limma package (moderated t-test) with Benjamini–Hochberg correction for multiple comparisons; significance was defined as FDR <0.05 and |log2(fold change)| >2 or <0.5. CPM counts per million mapped reads. (**F**) StreamGraph shows the sources of up- and down-regulated genes or isoforms (**H**). The black number represents the number of genes or isoforms (**H**).

To gain a deeper understanding of the ZSA induced by ZC3H14, we performed the third-generation sequencing (TGS) -based full-length transcriptome sequencing in single 1- and 2-cell mouse embryos. First, we evaluated the quality of TGS data. Each 2-cell embryonic sample was captured with over 8000 genes and 10,000 transcripts, but significantly decreased in the THZ531-treated group. Approximately 80% of the detected transcripts in each sample were known (Fig. EV5A,B; Dataset EV4). Moreover, both gene and transcript expressions showed high consistency within groups, but largely different between groups according to correlation and PCA analysis (Fig. EV5C,D). These results indicate that the single-embryo full-length transcriptome sequencing data are highly reliable.

The isoform structure analysis revealed a significant decrease full splice match (FSM) isoforms from 1-cell to 2-cell stage, indicating more alternative splicing events occurring. THZ531 treatment increased the FSM ratio at the 2-cell stage while reducing the Splice Match of NIC and NNC (Fig. 6J,K; Dataset EV4). Meanwhile, the number of Isoform Switch Events from 1-cell to 2-cell stages also significantly decreased after THZ531 treatment (Fig. 6L; Dataset EV4). These results further indicate that THZ531 reduces ZSA events.

### Aberrant alternative splicing leads to the accumulation of ptRNAs in the nucleus, causing developmental arrest in embryos

**Figure 7:**
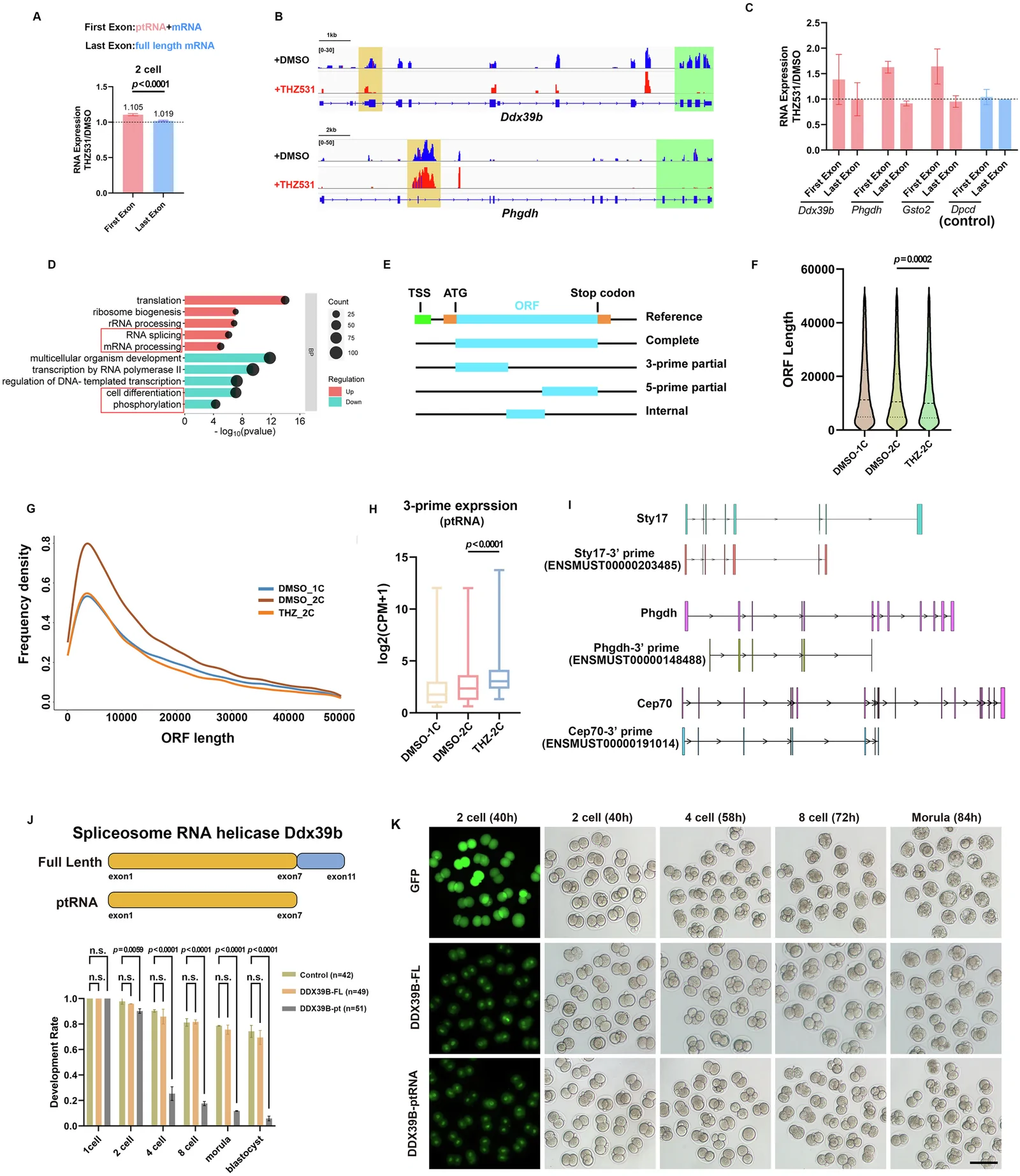
THZ531 hinders RNA degradation and leads to the accumulation of a large amount of ptRNAs in the nucleus, resulting in the retardation of zygote development. (**A**) RNA expression levels of the first and last exons in 2-cell embryos after being treated with THZ531. *n* = 3 biological replicates. Error bars, SEM. *P* < 0.0001 by two-tailed Student’s *t*-test. (**B**) Graphic representation of *Ddx39b* and *Phgdh* genes showing the reduction in read coverage in downstream portions of the transcript after THZ5311 treatment by the visualization results from the Integrative Genomics Viewer (IGV). (**C**) RNA expression in 2-cell embryos treated with THZ531 was measured by RT-qPCR for three genes with increased ptRNAs and one control gene. *n* = 3 biological replicates. Error bars, SEM. (**D**) Gene Ontology (GO) pathway analysis of the up- and down-regulated genes in a bar chart from full-length transcriptome seq. GO enrichment analysis was performed on the identified DEGs using the clusterProfiler package in R. The hypergeometric test was applied to assess the statistical significance of GO term enrichment. *P* values were corrected for multiple comparisons using the Benjamini–Hochberg method. GO terms with FDR <0.05 were considered significantly enriched. (**E**) Illustration showing the structure of different transcripts. TSS transcription start site, ORF open reading frame. (**F**, **G**) Data were presented as violin plots showing the distribution of ORF length across treatment groups (**F**). The three dashed lines within each violin indicate the median (25th percentile) and the first and third quartiles (25th and 75th percentiles). Statistical significance was assessed using one-way ANOVA followed by Tukey’s multiple comparisons test. *n* = 3 biological replicates. *P* values are indicated above the plots. ORF length in early embryos treated with THZ531 or DMSO (**G**). 1C 1-cell, 2C 2-cell, THZ THZ531. (**H**) Relative expression of 3’-prime partial (ptRNAs) derived from full-length transcriptome seq data, and the graphic representation of *Sty17*, *Phgdh*, and *Cep70* genes (**I**). Data were presented as box plots. The center line represents the median (50th percentile). The box spans the interquartile range (IQR) from the 25th to the 75th percentile. Whiskers extend to the most extreme data points within 1.5 × IQR from the box boundaries. Individual points beyond the whisker lines indicate outliers. The end of the lower whisker represents the minimum value among non-outliers, and the end of the upper whisker represents the maximum value among non-outliers. *n* = 3 biological replicates. *P* < 0.0001 by one-way ANOVA followed by Tukey’s test. (**J**) Development rates of cultured control, DDX39B-FL, and DDX39B-ptRNA overexpressing embryos. The experiment was independently repeated three times. The number of analyzed embryos are indicated (n). Error bars, SEM. Exact *P* values are indicated directly on the figure. Significance was determined by two-way ANOVA with a multiple comparisons test. (**K**) Representative images of preimplantation embryos at different stages after overexpression with GFP, *Ddx39b-*FL, and *Ddx39b-*ptRNA. FL full length*;* Scale bar, 100 μm. Source data are available online for this figure.

To investigate the effects of THZ531-mediated splicing on RNA levels, we tested for a global RNA phenotype by quantifying differential exon usage. We statistically analyzed the ratio of the expression levels of the first and last exons between DMSO and THZ531 treatment. The first exon represents both ptRNAs and full-length mRNAs, whereas the last exon represents only the full-length mRNA. The fold change between THZ531-treated two cells and the control group indicated that the average expression level of the first exon increased for global genes, whereas the average expression of the last exon remained unchanged. This suggested that ptRNA accumulated in THZ531-treated 2-cell embryos (Fig. 7A; Dataset EV5).

Through Integrative Genomics Viewer (IGV) visualization, it can be seen that the 5’ exons of two representative genes, *Ddx39b* and *Phgdh*, were transcribed completely, but the 3’ exons were missing (Fig. 7B). We performed RT-qPCR validation on four transcripts containing upregulated ptRNAs (*Ddx39b*, *Phgdh*, and *Gsto2*) and a control transcript (*Dpcd*). In THZ531-treated cells, the expression of the first exon of ptRNA was higher than that in the non-treated group, whereas the expression of the last exon was similar to that in the control group. The first and last exons of the control transcript *Dpcd* showed no changes after THZ531 treatment (Fig. 7C). These data confirm the increase in ptRNA levels in THZ531-treated 2-cell embryos. GO analysis of the upregulated and downregulated genes revealed enrichment of RNA splicing, mRNA processing, cell differentiation, and phosphorylation (Fig. 7D). To confirm this, we analyzed the TGS data, which showed that the global ORF length generally decreased after THZ531 treatment (Fig. 7E–G). Additionally, the 5’ prime expression significantly increased (Fig. 7H). Visualization software was used to observe the exon expression of three representative genes, *Sty17*, *Phgdh*, and *Cep70* (Fig. 7I), indicating the production of more ptRNAs in THZ531-treated 2-cells. To study the effects of ptRNA accumulation on early mouse embryonic development, we constructed *Ddx39b*-ptRNA with the last four exons deleted at the 3’ end and overexpressed it in mouse zygotes via microinjection. The control group was microinjected with GFP mRNA. We found that overexpression of full-length DDX39B (DDX39B-FL) did not affect embryo development, whereas the DDX39B-ptRNA construct caused significant 2-cell arrest (Fig. 7J,K). These results indicated that THZ531 caused aberrant alternative splicing, leading to the accumulation of large amounts of ptRNA in the nucleus and developmental arrest in early mouse embryos.

We analyzed differential gene expression. Under normal conditions, there were 1823 upregulated genes and 689 downregulated genes in 1-cell to 2-cell embryos. Compared with DMSO-treated 2-cell embryos, 1189 genes were downregulated, and only 714 genes were upregulated in THZ531-treated 2-cell embryos, consistent with the Smart-Seq2 results (Fig. EV5E). The 714 upregulated genes mainly belonged to the original stable and downregulated genes, whereas the 1189 downregulated genes belonged to the original stable and upregulated genes (Fig. EV5F). Changes in the isoforms were consistent with those in the genes (Fig. EV5G,H).

## Discussion

ZGA is a critical event in early embryonic development when the transcriptional activity of the embryonic genome increases significantly, resulting in a large number of transcripts. ZSA also occurs during this process, and the number of splicing events increases significantly. Zygote splicing ensures the correct maturation of mRNA and thus, the accuracy of gene expression. Erroneous splicing can lead to the accumulation of aberrant mRNA products, resulting in the retardation of early embryonic development. Despite the significance of ZSA, many unknown mechanisms regulate its mRNA processing and metabolism. Studying these mechanisms could enhance our understanding of the importance of mRNA splicing in early embryonic development and help address the challenges associated with embryonic development.

There are many different domains and nucleosomes in the cell nucleus that are involved in various aspects of gene expression (Smith et al, 2020; Galganski et al, 2017). ZC3H14 is crucial for the proper assembly of the PAXT complex, which involves other RNA processing proteins such as PABPN1, MTR4, and ZFC3H1 (Meola et al, 2016; Insco et al, 2023). Its expression is high during early embryonic stages, specifically in oocytes and early embryos. We observed dynamic changes in ZC3H14 during various developmental stages, and it showed different localization patterns. It formed nuclear speckles in growing oocytes and 2-cell embryos, but this formation diminished at other stages. The observation that ZC3H14 forms prominent nuclear speckles specifically during transcriptionally active stages (GO and 2-cell) and becomes diffuse during transcriptionally silent stages (FGO, MII) suggests a functional link between speckle localization and active RNA processing. Interestingly, ZC3H14 speckles often localize to DAPI-low regions, which correspond to transcriptionally permissive chromatin. The α-amanitin experiment demonstrates that active transcription is not required for speckle formation. The competence for ZC3H14 to form speckles is likely conferred by stage-specific phosphorylation. Once formed, these speckles may be positioned near active genes to facilitate co-transcriptional splicing, consistent with the known role of nuclear speckles as hubs for RNA processing.

Phenotypically, genetic *Zc3h14* knockout did not affect the development and growth of mice, but only affected fertility, highlighting its importance in germ cells. Importantly, ZC3H14 is not a general splicing factor required in all cell types. The observation that systemic Zc3h14 knockout mice are viable and develop normally, aside from reduced female fertility, does not contradict its essential role in early embryogenesis. Instead, this highlights a context-specific requirement for ZC3H14. During the unique developmental window of ZSA, the embryo must rapidly and accurately splice a massive wave of newly transcribed zygotic genes, placing an extraordinary demand on the splicing machinery that ZC3H14 helps to meet. In most somatic cells, functional redundancy with other RNA-binding proteins (e.g., ZC3H13) or alternative pathways likely compensates for the loss of ZC3H14, allowing for normal development.

It is worth noting that while Zc3h14 systemic knockout embryos exhibit a fully penetrant 2-cell arrest, THZ531 treatment results in a quantitatively less severe phenotype, with a small percentage of embryos developing beyond the 2-cell stage. This quantitative difference is likely due to the distinct nature of these interventions. Systemic knockout leads to a complete and permanent absence of ZC3H14 throughout oogenesis and early embryogenesis, whereas THZ531 is applied acutely and reversibly during a limited window after fertilization. Even at effective concentrations, incomplete target inhibition or rapid drug metabolism may allow a fraction of embryos to partially escape the arrest. Thus, the two approaches are complementary, revealing both the essentiality of ZC3H14 and the dose-dependent requirement for its phosphorylation. In addition, under our experimental conditions, THZ531 has no effect on the meiotic maturation of oocytes. This contrasts with a recent network analysis predicting ZC3H14 involvement (Deng et al, 2026), which predicted ZC3H14’s participation through network analysis but did not perform functional validation, emphasizing that predictive networks may not always reflect functional necessity, as there may be redundancy, such as with its homolog ZC3H13.

A key question in the field is whether nuclear speckle assembly is a cause or a consequence of active splicing. Our data support a causal role for ZC3H14 phosphorylation upstream of speckle formation and proper ZSA. First, the 4SA phospho-dead mutant fails to form speckles and causes developmental arrest, while the 4 SD phospho-mimetic partially rescues THZ531-induced defects. Second, inhibiting global splicing with PlaB does not prevent ZC3H14 speckle assembly, indicating that speckle formation is not merely a byproduct of splicing activity. Third, inhibiting transcription with α-amanitin does not affect ZC3H14 speckles, ruling out dependence on nascent ZGA transcripts. Together, these findings establish that CDK12/13-mediated phosphorylation of ZC3H14 drives speckle reassembly, which in turn facilitates efficient splicing of zygotic transcripts. However, the relationship is likely bidirectional: proper splicing may reinforce speckle integrity, creating a positive feedback loop. ZC3H14 phosphorylation thus acts as a critical node in this network, ensuring the rapid and accurate processing of the large wave of transcripts produced during ZGA.

Based on these observations, we explored the role of ZC3H14 in early embryonic development. Our study demonstrated that maternal ZC3H14 was essential for the initiation of early embryonic development. ZC3H14 phospho-mutants (4SA, mimicking the non-phosphorylated forms) led to developmental arrest at the 2-cell stage, indicating the importance of ZC3H14 phosphorylation in proper embryonic progression. Mutations at these phosphorylation sites disrupted the formation of nuclear speckles, with a more significant effect observed during the 2-cell stage. These results suggest that the failure of ZC3H14 to form nuclear speckles may be one of the reasons for the stunted embryonic development in mice.

It is important to distinguish between the contributions of maternally deposited ZC3H14 and zygotically expressed ZC3H14. Our knockout studies show that embryos lacking maternal ZC3H14 arrest at the 2-cell stage, and this arrest can be rescued by microinjecting *Zc3h14* mRNA into *Zc3h14*^*−/−*^ oocytes, confirming that the oocyte-supplied protein is essential. Whether zygotically expressed ZC3H14 (which would normally appear after ZGA) can also support development remains to be tested using zygote-specific knockout strategies. Nonetheless, the THZ531 inhibitor experiments, which act on fertilized eggs, clearly demonstrate that the phosphorylation-regulated function of ZC3H14 is required during the zygotic stage itself, independent of any potential contribution from newly synthesized protein.

ZC3H14 is not phosphorylated in CDK13-mutated cells (Insco et al, 2023). Our experimental results revealed that the inhibition of CDK12/13 using the small-molecule inhibitor THZ531 reduced the number of nuclear speckles in mouse oocytes and 2-cell embryos, further highlighting the critical role of CDK12/13-mediated ZC3H14 phosphorylation in nuclear speckle formation under developmental conditions. We explored the effects of phosphorylation on the nuclear speckle assembly. IF and co-IP experiments revealed that ZC3H14 directly interacted with PABPN1, and mutations in the phosphorylation sites hindered this interaction. Mutations in the phosphorylation sites led to the improper assembly of nuclear speckles. These results suggested that ZC3H14 phosphorylation was crucial for nuclear speckle assembly. CDK13 mutations lead to the accumulation of ptRNAs (Insco et al, 2023). Consistent with the results of previous studies, we found that a large number of incomplete transcripts were generated in THZ531-treated 2-cell embryos, especially those with deletions at the 5’ and 3’ ends, through full-length transcriptome sequencing analysis.

Our experiments with 293T cells, growing oocytes, and activated mouse embryos showed that ZC3H14 localized to nuclear speckles, which are subcellular membraneless structures in the nucleus rich in transcription, RNA processing, export-related factors, and a variety of noncoding RNAs. Additionally, ZC3H14 co-localized with the splicing factors SRSF2 and SRRM2, suggesting its involvement in RNA splicing and processing. RNA-seq analysis of embryos treated with THZ531 showed significant differences in gene expression, particularly at the 2-cell stage. We analyzed the variation in variable splicing events using rMATS software and found that THZ531 treatment resulted in a decrease in the number of global splicing events in 1–2-cell embryos.

SF3B1 acts as a splice factor (Yamauchi et al, 2022). The PlaB is a small-molecule inhibitor that specifically targets SF3B1 and effectively inhibits mRNA splicing (Kotake et al, 2007). Our study found that a large proportion of the genes downregulated by PlaB and THZ531 coincided, indicating that ZC3H14 and SF3B1 play partially synergistic roles in zygotic transcript splicing. However, the specific regulatory mechanisms underlying this process require further investigation.

Many questions regarding the role of ZC3H14 remain unanswered. Our study mainly focused on mouse models, which are species-specific despite the high similarity between mice and humans during embryonic development. Therefore, whether the results of this study apply to human embryonic development requires further investigation. To improve the wide applicability of this study, further comparative studies between species, such as human embryos, zebrafish, and other models, should be carried out. The functional differences and universality of ZC3H14 in different species were verified through systematic comparisons. Although this study revealed the effects of ZC3H14 phosphorylation on its localization and function, the interactions between ZC3H14 and other proteins and their detailed mechanisms of action remain unclear.

Because ZC3H14 is related to mRNA splicing and embryonic development, studying its role in the transition from embryonic totipotency to pluripotency may have clinical significance. As the incidence of infertility in humans increases, future research should explore the impact of ZC3H14 in this context, focusing on whether mutations occur in ZC3H14 in cases of infertility, and developing potential gene-targeted therapeutic strategies. Based on the regulation of ZC3H14 phosphorylation, specific small-molecule inhibitors or agonists could be developed in the future to modulate its function, thereby affecting mRNA splicing and related post-transcriptional processes, which may have significant clinical application value in the treatment of human reproductive diseases.

**Figure 8:**
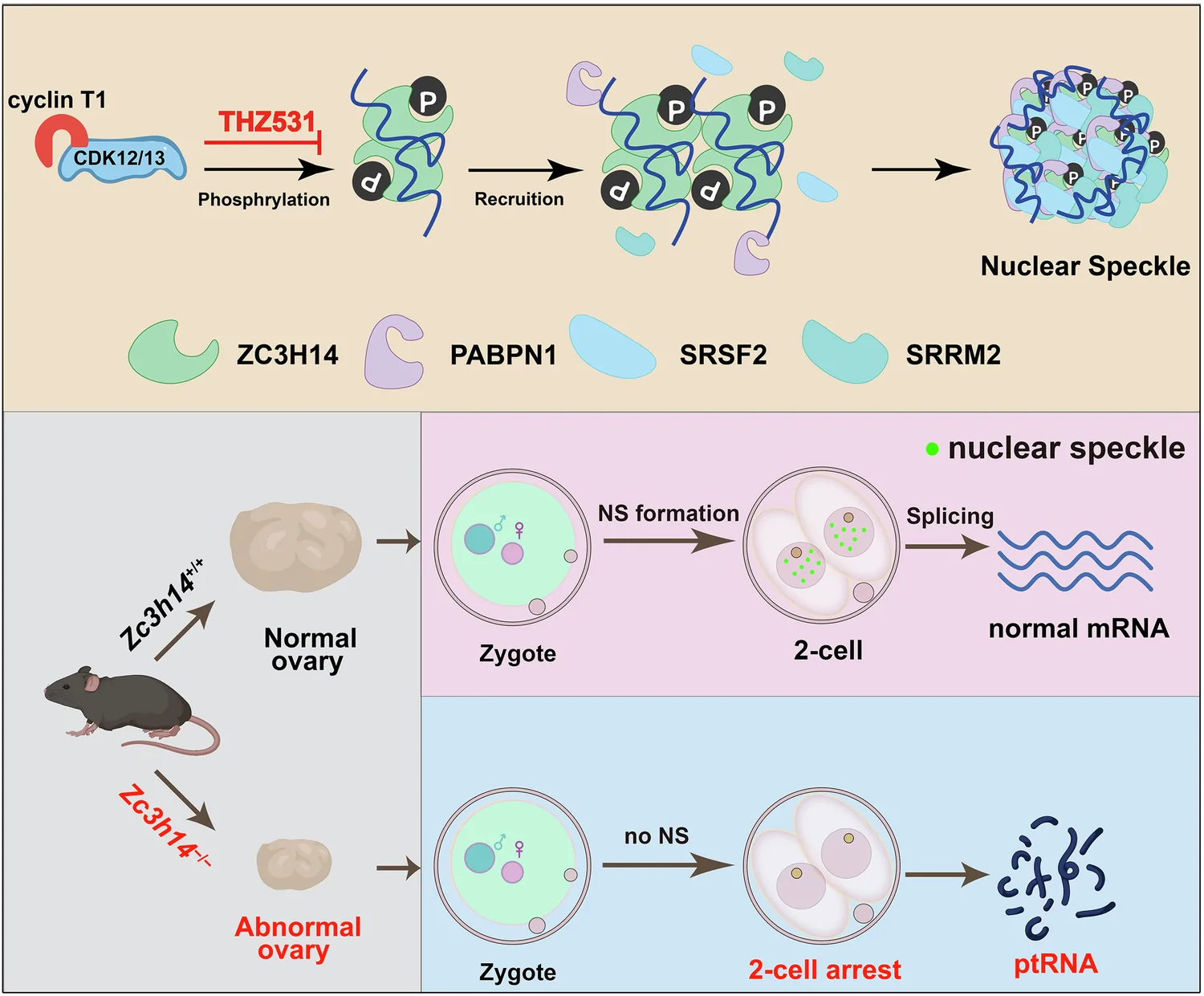
Pattern and functions of ZC3H14 during zygotic splicing activation and preimplantation development of mouse embryos. In the 2-cell embryos, many nascent transcripts are produced during ZGA. They need to be further spliced into mature mRNA within the nucleus and transported to the cytoplasm, a process typically performed by the spliceosome in a nuclear speckle. ZC3H14 is an important protein that promotes the phosphorylation-dependent formation of nuclear speckles at the 2-cell stage. CDK12/13 phosphorylates ZC3H14, enabling it to bind RNA. ZC3H14 then forms nuclear speckles by recruiting several other proteins, such as PABPN1, SRRM2, and SRSF2. The 2-cell embryos of *Zc3h14*^*–/–*^ mice are unable to form nuclear speckles. As a result, the RNA splicing process is disrupted, leading to the production of many abnormal RNAs and causing embryonic development arrest at the 2-cell stage.

In conclusion, our study revealed for the first time that ZC3H14 is present in the nuclear speckles of mouse oocytes and early embryos and that its localization is associated with transcriptional activity and shows dynamic changes. It is crucial for early mouse embryonic development, and its phosphorylation regulates its localization. Our study demonstrates that mutations in the phosphorylation site of ZC3H14 affects its binding to other components of the spliceosome, revealing that aberrant mRNA splicing regulated by ZC3H14 phosphorylation can lead to ptRNA accumulation in the nucleus, which in turn leads to the arrest of early embryonic development (Fig. 8). In summary, these results expand our understanding of the multiple roles of ZC3H14 in post-transcriptional regulation during early embryonic development.

## Methods

**Table:** Reagents and tools table

Reagent/resource	Reference or source	Identifier or catalog number
**Experimental models**		
*Zc3h14*^*−/−*^, C57BL/6 (*M. musculus*)	https://www.cyagen.cn/mice-bank/S-KO-18322	S-KO-18322
Wild-type, C57BL/6J (*M. musculus*)	Zhejiang University Laboratory Animal Center	N/A
**Recombinant DNA**		
pRK5-FLAG-GFP-ZC3H14 (WT full-length)	This laboratory	N/A
pRK5-FLAG-GFP-ZC3H14 (4SA mutant: S343A/S409A/S475A/S515A)	This laboratory (site‑directed mutagenesis)	N/A
pRK5-FLAG-GFP-ZC3H14 (4 SD mutant: S343D/S409D/S475D/S515D)	This laboratory (site‑directed mutagenesis)	N/A
pRK5-FLAG-GFP (empty vector)	This laboratory	N/A
Ddx39b-FL (full-length)	This laboratory	N/A
Ddx39b-ptRNA (truncated, lacking last 4 exons)	This laboratory	N/A
**Antibodies**		
Anti-ZC3H14 (rabbit polyclonal)	Thermo Fisher Scientific	Cat# 289190-1-AP
Anti-PABPN1 (rabbit polyclonal)	Bethyl	Cat# A303-523A
Anti-SRSF2 (mouse monoclonal)	Sigma-Aldrich	Cat# SAB4200725
Anti-SRRM2 (rabbit polyclonal)	Proteintech	Cat# 30741-1-AP
Anti-CDK13 (rabbit polyclonal)	Abcam	Cat# ab170161
Anti-GAPDH (mouse monoclonal)	Proteintech	Cat# 60004-1-Ig
Anti-FLAG (mouse monoclonal)-	Sigma-Aldrich	Cat# F3165
Anti-HA tag (rabbit monoclonal)	Cell Signaling Technology	Cat# 3724
Anti-RNA Pol II pS2 (rabbit polyclonal)	Abcam	Cat# ab5095
Anti-α-Tubulin (mouse monoclonal)	Sigma-Aldrich	Cat# F2168
Anti-rabbit IgG-HRP (secondary)	Cell Signaling Technology	Cat# 7074
Alexa Fluor® 488-conjugated anti-mouse/rabbit (secondary)	Thermo Fisher Scientific	Cat# A-11001, A-11008
Alexa Fluor® 594-conjugated anti-mouse/rabbit (secondary)	Thermo Fisher Scientific	Cat# A-11012, A-11005
Alexa Fluor® 647-conjugated anti-mouse/rabbit (secondary)	Thermo Fisher Scientific	Cat# A-21235, A-21244
**Oligonucleotides and other sequence-based reagents**		
Primers	This study	Table EV2
siRNA	This study	Table EV2
**Chemicals, enzymes and other reagents**		
THZ531 (CDK12/13 inhibitor)	MedChem Express	Cat# HY-103618;
Pladienolide B (PlaB, SF3B1 inhibitor)	Selleck Chemicals	Cat# S7176
α-Amanitin (transcription inhibitor)	Sigma-Aldrich	Cat# A2263
Pregnant Mare Serum Gonadotropin (PMSG)	Ningbo Second Hormone Factory https://www.nshf.com/products/Serum-Gonadotrophin-for-Injection.html	N/A
Human Chorionic Gonadotropin (hCG)	Ningbo Second Hormone Factory https://www.nshf.com/products/dqs.html	N/A
Hyaluronidase	Sigma-Aldrich	Cat# H3506
M2 medium	Sigma-Aldrich	Cat# M7167
M16 medium	Sigma-Aldrich	Cat# M7292
KSOM medium	Millipore	Cat# MR-020 P-5D
Mineral oil	Sigma-Aldrich	Cat# M5310
TRIzol Reagent	Thermo Fisher Scientific	Cat# 15596026
RNeasy Mini Kit	QIAGEN	Cat# 74104
Power SYBR® Green PCR Master Mix	Applied Biosystems	Cat# 4367659
5-Ethynyl uridine (EU)	Thermo Fisher Scientific	Cat# E10345
Click-iT® RNA Alexa Fluor® 488 Imaging Kit	Thermo Fisher Scientific	Cat# C10329
mMESSAGE mMACHINE™ T7 Transcription Kit	Invitrogen	Cat# AM1344
Poly(A) Tailing Kit	Invitrogen	Cat# AM1350
TruePrep DNA Library Prep Kit V2	Vazyme	Cat# TD503
ERCC RNA Spike-In Mix	Thermo Fisher Scientific	Cat# 4456740
Ampure XP Beads	Beckman Coulter	Cat# A63882
**Software**		
TopHat	https://ccb.jhu.edu/software/tophat	v2.0.6
Cuffquant / Cuffnorm	http://cole-trapnell-lab.github.io/cufflinks	N/A
rMATS	https://rnaseq-mats.sourceforge.io	v.4.1.1
SMRT-Link	https://www.pacb.com	v.8.0.0.80529
scISA-Tools	https://github.com/shizhuoxing/scISA-Tools	N/A
SQANTI3	https://github.com/ConesaLab/SQANTI3	N/A
TransDecoder	https://github.com/TransDecoder	v.5.5.0
IsoformSwitchAnalyzeR	https://bioconductor.org/packages/IsoformSwitchAnalyzeR	N/A
ImageJ	https://imagej.nih.gov/ij/	N/A
Prism	https://www.graphpad.com	v.9.0
R	https://www.r-project.org	v.4.0.3
**Other**		
Microinjection system (TransferMan® NK2)	Eppendorf	N/A
Confocal microscope (LSM710)	Zeiss	N/A
Rea-time PCR system (ABI 7500)	Applied Biosystems	N/A
PacBio Sequel IIe sequencing platform	PacBio	N/A
Illumina NovaSeq 6000 sequencing platform	Illumina	N/A

### Mice

The *Zc3h14*^*−/−*^ mouse strain was constructed using CRISPR-Cas9-based gene targeting. A systemic knockout of the *Zc3h14* gene was achieved on the C57BL/6JCya background, targeting Exon 13, with a deletion fragment of about 0.1 kb. The *Zc3h14*^*−/−*^ mice used in this study were purchased from Cyagen Biosciences. More information about the mice can be accessed through the following link: https://www.cyagen.cn/mice-bank/S-KO-18322. All mice were housed under specific pathogen-free conditions in a constant environment, including a temperature of 20–22 °C, a 12/12-h light/dark cycle, 50–70% humidity, and food and water provided ad libitum. The animal care and experimental procedures were conducted in accordance with the guidelines of the Animal Research Committee of Zhejiang University.

### Oocyte culture

Three-week-old female mice were intraperitoneally primed with 5 IU of pregnant mare serum gonadotropin (PMSG) and were humanely euthanized after 44 h. Fully grown oocytes were harvested in M2 medium (M7167; Sigma-Aldrich) and cultured in mini-drops of M16 medium (M7292; Sigma-Aldrich) covered with mineral oil (M5310; Sigma-Aldrich) and then incubated at 37 °C in a 5% CO_2_ atmosphere.

### Superovulation and fertilization

Three-week-old female mice were intraperitoneally injected with 5 IU PMSG. After 44 h, the mice were injected with 5 IU hCG. After an additional 16 h, oocyte-cumulus complexes were retrieved from the oviducts, and oocytes were obtained after treatment with hyaluronidase (Sigma-Aldrich). To obtain early embryos, female mice were mated with 10–12-week-old WT males. Successful mating was confirmed by the presence of vaginal plugs. Embryos were harvested from the oviducts at the indicated time points after hCG injection.

### FlSH assay

The oocytes were washed with 0.2% bovine serum albumin (BSA) and transferred to 4% PFA for 25 min at room temperature. Next, the oocytes were permeabilized at room temperature for 20 min with 2% Triton X-100 and 1× RNA ribonuclease (RNase) inhibitor and transferred to a wash buffer (preheated to 37 °C) containing 1× RNA RNase inhibitor for 15 min. The oocytes were then transferred to a hybridization buffer into which a probe was added and allowed to incubate in a thermostat incubator overnight at 37 °C in the dark. This was followed by washing of the oocytes in warm wash buffer for at least 50 min at 37 °C and a rinse in 2× saline-sodium treat for 2 min at room temperature. Next, the oocytes were incubated with appropriate primary and secondary antibodies, similar to those used for IF.

### Treatment of mouse embryos with THZ531, α-amanitin and PlaB

Zygotes were collected from the oviducts 22 h after hCG treatment. Zygotes were cultured in potassium simplex optimized medium (KSOM) supplemented with THZ531 (MedChem Express, Monmouth Junction, NJ, USA; Cat. No.: HY-103618). The concentration of THZ531 (1 µM) was chosen based on published studies (Zhang et al, 2016) and our preliminary toxicity assays. Zygotes were cultured in KSOM supplemented with 1 µM THZ531 from 22 h post-hCG for 24 h. For α-amanitin and PlaB treatments, concentrations of 25 µg/µl and 100 nM were used, respectively, based on established protocols (Zhang et al, 2024a). To confirm that 1 µM THZ531 did not affect global transcription, we performed 5-ethynyl uridine (EU) incorporation and RNA polymerase II Ser2 phosphorylation (pS2) staining on treated embryos, which showed no significant difference from controls (Fig. EV3A,B).

### Plasmid construction and in vitro transcription

The resulting cDNA was subcloned into a FLAG-tagged expression vector (pRK5). The expression vector was linearized with HindIII, and the linearized RNA was transcribed in vitro using the T7 mMESSAGE mMACHINE kit (Invitrogen, Carlsbad, CA, USA; AM1344). Phenol-chloroform and ethanol precipitation were used for RNA extraction and purification, respectively. Transcribed RNAs were polyadenylated in vitro using a poly (A) tailing kit (AM1350; Invitrogen).

### Microinjection of mRNAs

Microinjections were performed using an Eppendorf TransferMan NK2 micromanipulator. Fully grown oocytes were cultured in M2 medium containing 2 μM milrinone to suppress spontaneous germinal vesicle breakdown, and zygotes were cultured in potassium simplex optimized medium (KSOM). Approximately 5–10 pL of 500–1000 ng/µL mRNA was microinjected into the oocyte cytoplasm or fertilized eggs at 20–24 h post-hCG injection. As a negative control, 5–10 pL of 500 μg/mL RNA transcribed from the empty vector (pRK5-Flag-GFP) was microinjected into the control oocytes. The oocytes and zygotes were cultured in M16 or KSOM medium at 37 °C in a 5% CO_2_ atmosphere.

### EU incorporation assay

Embryos were cultured in KSOM containing 100 μM EU for 2 h. Fixation, permeabilization, and staining were performed using the Click-iT® RNA Alexa Fluor® 488 Imaging Kit (Thermo Fisher Scientific, Waltham, MA, USA; 48 C10329) according to the manufacturer’s protocol. The embryos were imaged using a Zeiss LSM710 confocal microscope (Oberkochen, Germany).

### Library preparation and data analysis of poly(A) RNA-seq

Embryos were collected from 3-week-old female WT mice (10 embryos per sample). Each sample was directly lysed with 2 mL lysis buffer (0.2% Triton X-100 and RNase inhibitor) and 0.2 mL of 1:1000 diluted ERCC. The RNA in the cell lysis buffer was reverse-transcribed using the Smart-seq2 method. qRT-PCR analysis of cDNA quality was conducted to confirm that the main peaks of the cDNA library pool were longer than 1000 bp. The full-length cDNA library was fragmented using a TruePrep DNA Library Prep Kit V2 from Illumina (Vazyme, TD503). DNA fragments were selected, and the final sequencing libraries were constructed from 300 to 400 bp of the amplified cDNA. Sequencing data were extracted on the Illumina NovaSeq 6000 platform in 151 bp paired-end mode. A script was used to filter low-quality reads, and the clean reads were mapped to the mouse genome mm10 using TopHat (version 2.0.6). Gene expression levels were calculated and normalized to FPKM using cuffquant and cuffnorm. The absolute mRNA copy number was calculated using the spiked-in ERCC molecules. FPKM and mRNA copy number values were used to represent the gene expression levels. Only the expressed genes (FPKM >1 in at least one sample) were considered in subsequent analyses.

Genes with fold change >2 and *p* value <0.05 were considered as differentially expressed genes. Gene transfer format (gtf) documents, including only the first or last exons, were generated separately using standard gtf documents according to the exon sorting number. The expression levels of the first or last exons were quantified from separate gtf documents with uniquely mapped reads. Statistical analyses and figure drawings were performed using R software (http://www.rproject.org). The Spearman correlation coefficient (rs) was calculated using the “cor” function, and the complete linkage hierarchical algorithm was used to cluster the genes. Differential gene expression analysis was performed using the limma package in R software.

### Splicing factor analysis

The list of total splicing factors was derived from published articles and “RNA splicing”—or “spliceosome”—associated GO terms from MGI47-50. All splicing factors in mice and those associated with THZ531 treatment are listed in Dataset EV3.

### Single-cell cDNA amplification and TGS library construction for PacBio sequencing

We used the same amplification procedure as for SCAN-seq (Fan et al, 2020), except for changing the reverse transcription primer to a 10× gel bead for each reaction of the embryonic samples. Each pre-amplification product was purified using 0.6×Ampure XP beads (Beckman, Cat. A63882). The concentrations were measured using Qubit dsDNA HS and BR Assay Kits (Invitrogen, Cat. Q32854). PCR products from approximately 60 blastomeres that were confirmed to be effectively amplified were pooled in proportion to the number of amplified cycles. We took 100 ng of the pooled cDNA to build the PacBio sequencing library following the protocol of HIT-scISOseq (Shi et al, 2023) and sequenced on the PacBio sequel IIe.

### Single-cell full-length transcript sequencing data pre-processing

Stand-alone versions of the SMRT-Link (version 8.0.0.80529) software package were used to transform raw Subreads to Calling Circular Consensus Sequencing (CCS) reads with the following parameters: min passes 0, min length 50, max length 21,000, and min rq 0.75. After CCS calling, we used the HIT-scISOseq analysis software kit scISA-Tools (https://github.com/shizhuoxing/scISA-Tools) for Full-length non-concatemer (FLNC) read identification, cell barcoding, and UMI extraction and correction.

### Alignment and generation of a single-cell gene/isoform expression matrix

After trimming the primers, cell barcodes, UMIs, and poly(A) tails, the remaining FLNC sequences were aligned to the mouse genome (10x Genomics pre-build mouse mm10 reference dataset: refdata-gex-mm10-2020-A) using minimap2 (version 2.17-r974-dirty) in spliced alignment mode with the following parameters: “-ax splice-uf—secondary = no-C5.” We then used gffcompare (version 0.11.6) to assign the mapped FLNCs to mm10 annotation gene models (10x Genomics pre-build mouse mm10 reference dataset: refdata-gex-mm10-2020-A) based on the FLNCs genome alignment SAM file. Based on the identified cell barcodes, we used the scGene_matrix utility of scISA-Tools to generate a single-cell gene expression matrix. Expression values were normalized to copy numbers per 100,000 mapped reads (CPM/10). Gene and isoform expression in these experiments is listed in Dataset EV4.

### Isoform type classification by ORF prediction

After processing using SQANTI3, the FASTA file of the mapped genome sequences was extracted according to the SQANTI3 output GTF file. Based on the FASTA file, TransDecoder (v5.5.0) was used for ORF extraction and prediction. For those predicted to have multiple ORFs, the longest ones were selected as the representative ORF. The TransDecoder assigns each detected isoform to one of four types based on whether it contains the start and stop codons of the reference ORF: complete, 5-prime partial, 3-prime partial, and internal. Additionally, we assigned isoforms that did not map to an ORF region by TransDecoder. The isoform types and ORF lengths used in these experiments are listed in Dataset EV4.

### Isoform switch analysis

Based on the isoform expression matrix, we used IsoformSwitchAnalyzeR to identify switch isoforms between each pair of adjacent embryonic stages under the criteria of isoform_switch_q_value <0.05 and gene_switch_q_value <0.01.

### Analysis of ASEs

Using replicate multivariate analysis of transcript splicing (rMATS) (Shen et al, 2012, 2014), we detected five ASEs: SE, A5SS, A3SS, MXE, and retained introns. Various ASEs were considered when the false discovery rate (FDR) was <0.05, and the IncLevel difference was >0.1 or <−0.1.

### Statistical analysis

Only littermates were used as WT control and knockout mice. Samples of the same genotype and age were randomly allocated to experimental groups. The investigators were blinded to the group allocation during oocyte manipulation and RNA-seq data collection and analysis, and oocytes with signs of degeneration before or after culture were excluded from the analyses. Data were presented as means ± SEM. For experiments involving embryo development, “*n*” represents the number of embryos analyzed from at least three independent biological replicates (each replicate using embryos from different litters). For other experiments, “*n*” represents the number of biological replicates. A two-tailed unpaired Student’s *t*-test was used to compare the different groups. Multi-group comparison experiments were used with one-way ANOVA followed by Tukey’s test, or two-way ANOVA with a multiple comparisons test. All exact *P* values are indicated directly on the figure. “n.s.” indicates nonsignificant.

### WB analysis

Oocytes were lysed in a loading buffer containing β-mercaptoethanol and then heated at 95 °C for 10 min. Immunoblotting and sodium dodecyl sulfate–polyacrylamide gel electrophoresis were performed according to standard procedures using the Mini-PROTEAN Tetra Cell System (Bio-Rad, Hercules, CA, USA). All antibodies used are listed in Table EV1.

### IF assay

The oocytes and embryos were fixed with 4% PFA for 30 min and permeabilized for 20 min with 0.3% Triton X-100 in phosphate-buffered saline. Antibody staining was performed as previously described44. Imaging was performed using a Zeiss LSM710 confocal microscope. Quantitative analysis of the fluorescence signals was performed using the ImageJ software. The antibodies used in the experiments are listed in Table EV1.

### Quantitative RT-PCR

Oocytes or embryos were collected from 3-week-old female mice (10 oocytes or embryos per sample). Total RNA was extracted from oocytes using the RNeasy Mini Kit (QIAGEN, Hilden, Germany) according to the manufacturer’s instructions. Reverse transcription was performed using the Superscript RT kit (Bio-Rad). Quantitative RT-PCR was conducted using Power SYBR Green PCR Master Mix (Applied Biosystems, Life Technologies) on an ABI 7500 Real-Time PCR System (Applied Biosystems, Waltham, MA, USA). The primers are listed in Table EV2.

## Supplementary information

Table EV1

Table EV2

Peer Review File

Dataset EV1

Dataset EV2

Dataset EV3

Dataset EV4

Dataset EV5

Source data Fig. 1

Source data Fig. 2

Source data Fig. 3

Source data Fig. 4

Source data Fig. 5

Source data Fig. 6

Source data Fig. 7

Expanded View Figures

## Data Availability

Previously published RNA-seq data about PlaB-treated embryos used in this work were from the National Center for Biotechnology Information (NCBI) with the accession number PRJNA004899. The datasets generated during the current study are available in the GEO accession number GSE306152 of smart-seq2 (https://www.ncbi.nlm.nih.gov/geo/query/acc.cgi?acc=GSE306152), and the GEO accession number GSE306643 of full-length seq (https://www.ncbi.nlm.nih.gov/geo/query/acc.cgi?acc=GSE306643). The source data of this paper are collected in the following database record: biostudies:S-SCDT-10_1038-S44319-026-00950-x.
