# Supplementary material for: ZC3H14 phosphorylation is required for nuclear speckle assembly during zygotic splicing activation in mice
Source: EMBO Rep. 2026 Oct 7;27(21):6396–424. doi: 10.1038/s44319-026-00950-x (PMC13743749; doi:10.1038/s44319-026-00950-x)
Supplement: Supplementary file 1 [file 44319_2026_950_MOESM16_ESM.pdf]

Expanded View Figures

Figure EV1. Localization and expression of ZC3H14 in oocytes and early mouse embryos.

(**A**) Components of the PAXT complex include ZFC3H1, ZC3H14, PABPN1, and MTR4. (**B**) Expression level of PAXT components at different stages in mouse oocytes and early embryos. $n = 3$ biological replicates. Error bars, SEM. (**C**) Immunofluorescent staining showing the speckles of GFP-ZC3H14 in 293 T cells. Scale bars, 10 µm. (**D**) Western blotting results showing the expression of GFP-ZC3H14 in 293T. (**E**) Immunofluorescent staining showing the same speckles of GFP-ZC3H14 and endo-ZC3H14 in 293 T cells. Scale bars, 10 µm. (**F**) Immunofluorescent staining showing the co-localization of SRSF2 and endo-ZC3H14 in 293T cells. Scale bars, 10 µm. (**G**) Immunofluorescence staining showing the expression and localization of GFP-ZC3H14 in mouse oocytes and early embryos at different stages. Green, GFP-ZC3H14; red, DNA; The white dashed border highlights the membrane of the oocyte or embryo. scale bar, 20 µm.

 © The Author(s)

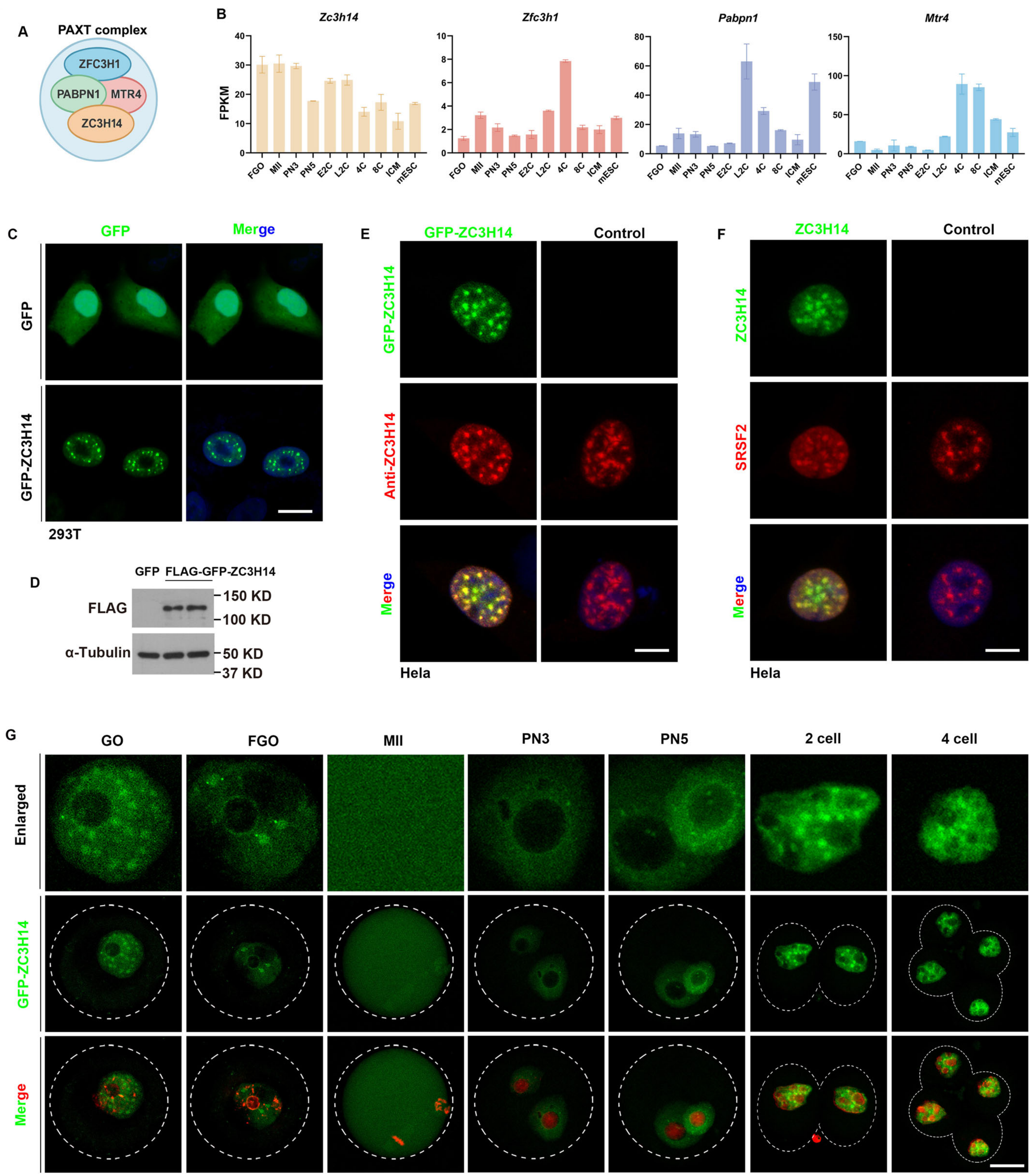
A
PAXT complex
ZFC3H1
PABPN1
MTR4
ZC3H14
B
Zc3h14
Zfc3h1
Pabpn1
Mtr4
FPKM
FGO MII PN3 PN5 E2C L2C 4C 8C ICM mESC
C
GFP
Merge
GFP-ZC3H14
293T
D
GFP FLAG-GFP-ZC3H14
FLAG
α-Tubulin
150 KD
100 KD
50 KD
37 KD
E
GFP-ZC3H14
Control
Anti-ZC3H14
Merge
Hela
F
ZC3H14
Control
SRSF2
Merge
Hela
G
GO FGO MII PN3 PN5 2 cell 4 cell
Enlarged
GFP-ZC3H14
Merge

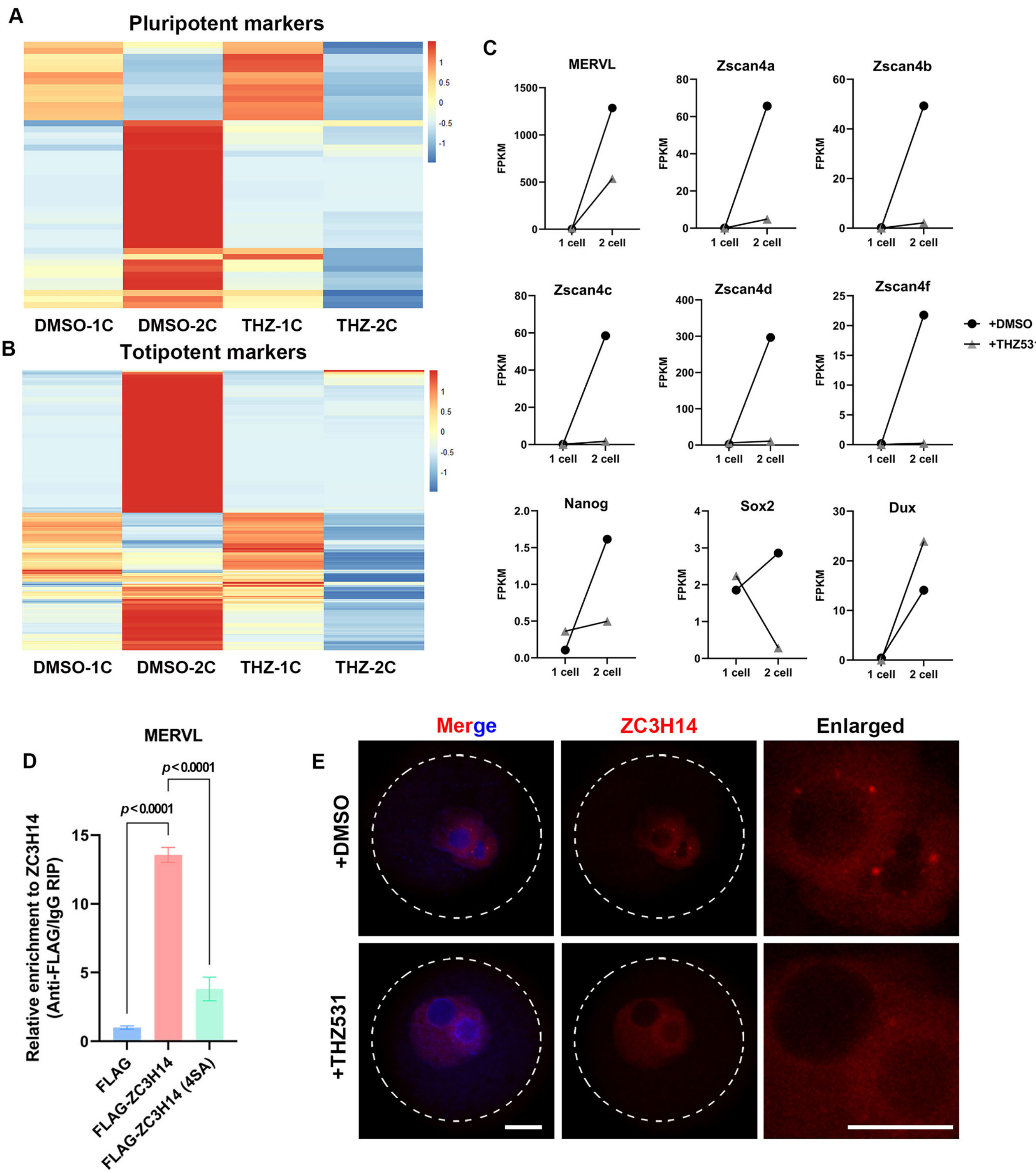

Figure EV2. The effect of THZ531 on the expression levels of pluripotent and multipotent genes.

(**A**) Heatmap shows the relative expression levels of representative pluripotent genes in 1-cell and 2-cell embryos treated with THZ531 or DMSO. (**B**) Heatmap shows the relative expression levels of representative totipotent genes in 1-cell and 2-cell embryos treated with THZ531 or DMSO. (**C**) The expression levels of some totipotent and pluripotent genes in 2-cell embryos treated with THZ531 or DMSO. (**D**) RNA immunoprecipitation (RIP) results showing the interaction of ZC3H14 with indicated transcripts. RNAs recovered from the immunoprecipitants were subjected to RT-qPCR of the indicated transcripts. The experiment was independently repeated three times. Error bars, SEM. $P < 0.0001$ by one-way ANOVA followed by Tukey's test. (**E**) Immunofluorescent staining showing the speckles of ZC3H14 in 1-cell embryos after treatment with THZ531 or DMSO. The white dashed border highlights the membrane of the oocyte or embryo. Scale bar, 20 μm.

 © The Author(s)

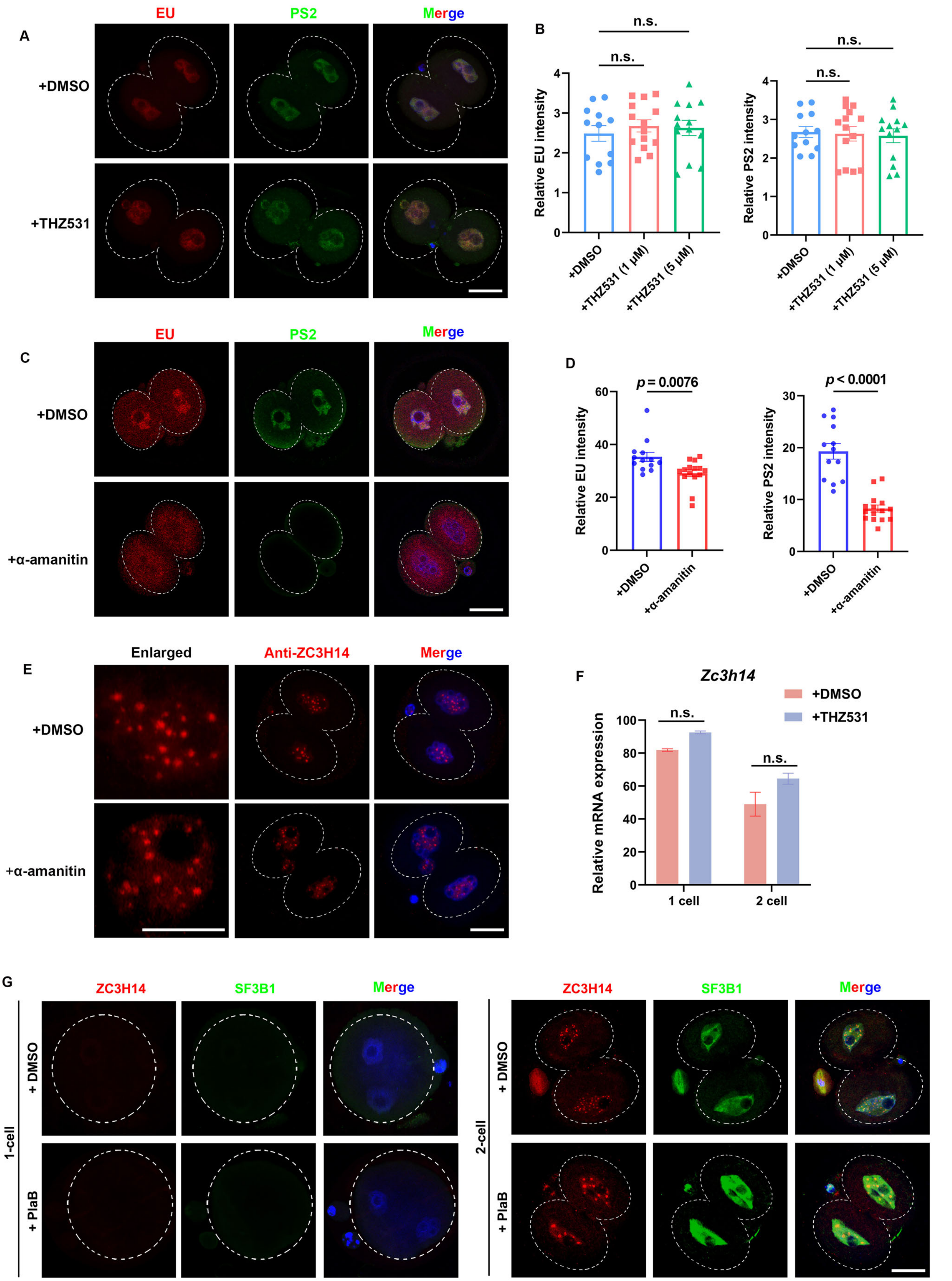
A
EU
PS2
Merge
+DMSO
+THZ531
B
n.s.
n.s.
Relative EU intensity
+DMSO
+THZ531 (1 μM)
+THZ531 (5 μM)
n.s.
n.s.
Relative PS2 intensity
C
EU
PS2
Merge
+DMSO
+α-amanitin
D
p = 0.0076
Relative EU intensity
p < 0.0001
Relative PS2 intensity
+DMSO
+α-amanitin
E
Enlarged
Anti-ZC3H14
Merge
+DMSO
+α-amanitin
F
Zc3h14
+DMSO
+THZ531
n.s.
n.s.
Relative mRNA expression
1 cell
2 cell
G
ZC3H14
SF3B1
Merge
1-cell
+ DMSO
+ PlaB
2-cell

Figure EV3. Speckle formation by ZC3H14 is not dependent on transcription and active splicing.

(**A**) Immunofluorescence staining showing the fluorescence intensity of EU and pS2 in 2-cell embryos after treatment with THZ531 or DMSO. The white dashed border highlights the membrane of the oocyte or embryo. Scale bar, 20 μm. EU ethynyl uridine, pS2 phosphorylated RNA polymerase II. (**B**) ImageJ was used to quantitatively analyze the relative EU and pS2 intensity in (**A**). The number of analyzed embryos are indicated (n). Error bars, SEM. n.s. nonsignificant, by one-way ANOVA followed by Tukey's test. (**C**) Immunofluorescence staining showing the fluorescence intensity of EU and pS2 in 2-cell embryos after treatment with α-amanitin (25 μM) or DMSO. The white dashed border highlights the membrane of the oocyte or embryo. Scale bar, 20 μm. (**D**) ImageJ was used to quantitatively analyze the relative EU and pS2 intensity in (**C**). The number of analyzed embryos are indicated (n). Error bars, SEM. Exact *P* values are indicated directly on the figure. Significance was determined by a two-tailed Student's *t*-test. (**E**) Immunofluorescence staining showing the speckles of ZC3H14 in 2-cell embryos after treatment with α-amanitin (25 μM) or DMSO. The white dashed border highlights the membrane of the oocyte or embryo. Scale bar, 20 μm. (**F**) The relative RNA expression level of ZC3H14 in 1-cell and 2-cell embryos after treatment with THZ531 (1 μM) or DMSO. $n = 3$ biological replicates. Error bars, SEM. n.s. nonsignificant, by a two-tailed Student's *t*-test. (**G**) Immunofluorescent staining showing the speckles of ZC3H14 and the expression level of SF3B1 in zygote and 2-cell embryos after treatment with PlaB (100 nM) or DMSO. The white dashed border highlights the membrane of the oocyte or embryo. Scale bar, 20 μm.

 © The Author(s)

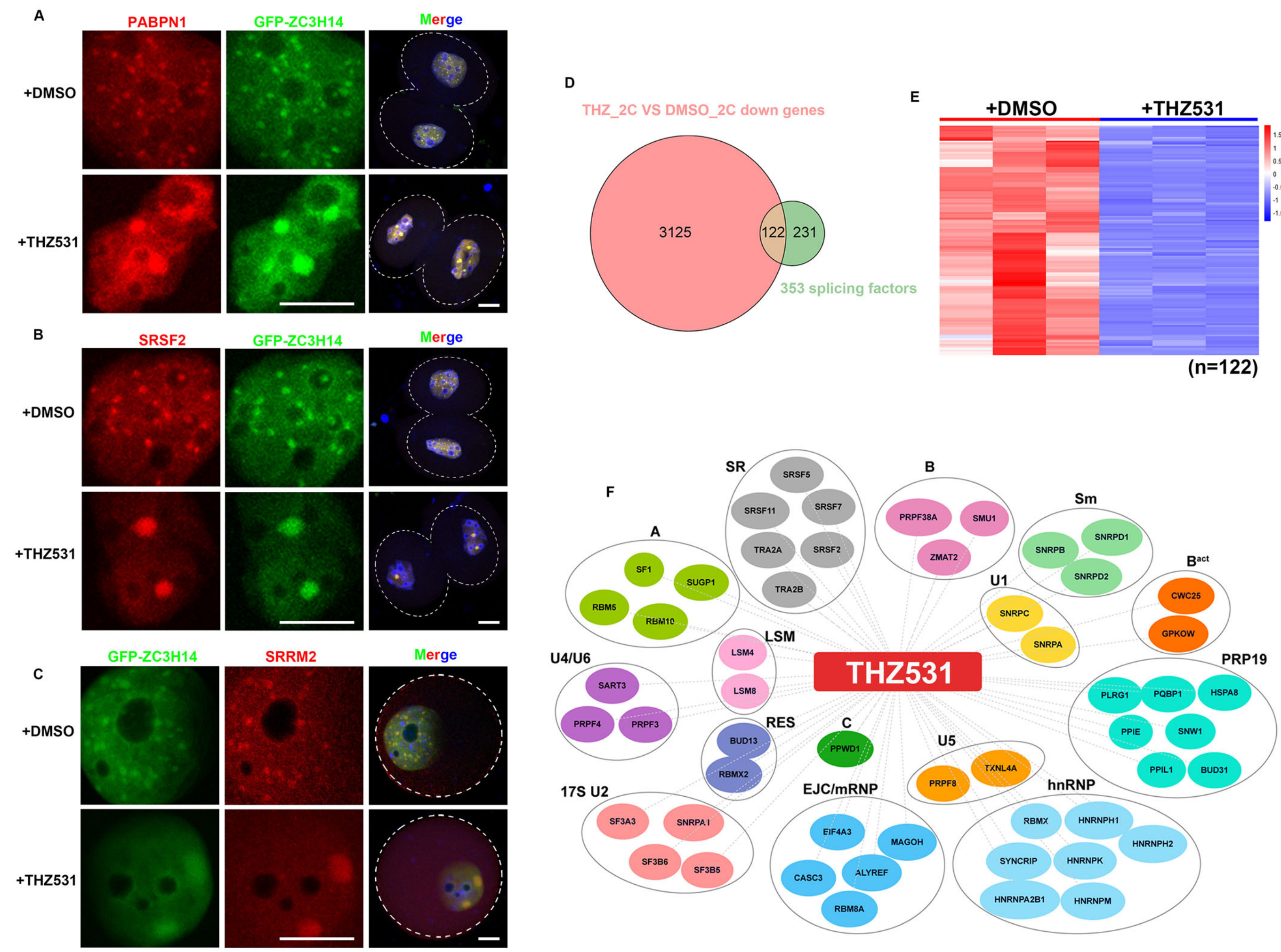

Figure EV4. Effects of THZ531 on alternative splicing and formation of nuclear speckles.

(**A**) Immunofluorescent staining showing the speckle scales of ZC3H14 and PABPN1 in 2-cell embryos after treatment with THZ531 (1 µM) or DMSO. The white dashed border highlights the membrane of the oocyte or embryo. Scale bar, 20 µm. (**B**) Immunofluorescent staining showing the speckle scales of ZC3H14 and SRSF2 in 2-cell embryos after treatment with THZ531 (1 µM) or DMSO. The white dashed border highlights the membrane of the oocyte or embryo. Scale bar, 20 µm. (**C**) Immunofluorescent staining showing the speckle scales of ZC3H14 and SRRM2 in growing oocyte after treatment with THZ531 (1 µM) or DMSO. The white dashed border highlights the membrane of the oocyte or embryo. Scale bar, 20 µm. (**D**) Venn diagrams showing the overlap of downregulated genes from THZ531 treatment and 353 splicing factors. (**E**) Heatmap showing the normalized expression levels of 122 splicing factors from (**B**) in 2-cell embryos treated with DMSO or THZ531. (**F**) Spliceosome components affected by THZ531 from RNA-seq data.

© The Author(s)

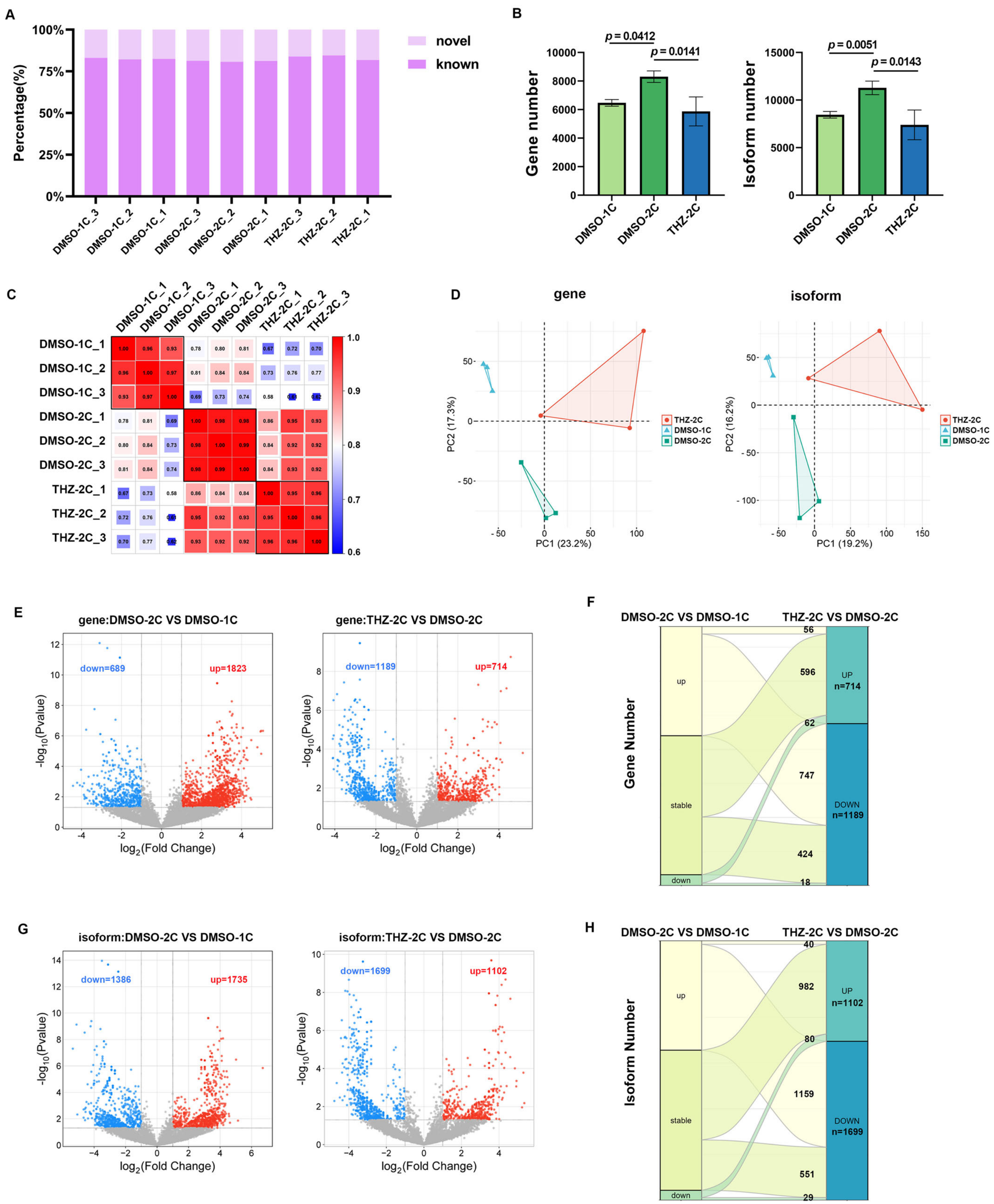
A
B
C
D
E
F
G
H
novel
known
Percentage(%)
Gene number
Isoform number
p = 0.0412
p = 0.0141
p = 0.0051
p = 0.0143
gene
isoform
PC1 (23.2%)
PC2 (17.3%)
PC1 (19.2%)
PC2 (16.2%)
THZ-2C
DMSO-1C
DMSO-2C
gene:DMSO-2C VS DMSO-1C
gene:THZ-2C VS DMSO-2C
down=689
up=1823
down=1189
up=714
isoform:DMSO-2C VS DMSO-1C
isoform:THZ-2C VS DMSO-2C
down=1386
up=1735
down=1699
up=1102
-log10(Pvalue)
log2(Fold Change)
DMSO-2C VS DMSO-1C
THZ-2C VS DMSO-2C
Gene Number
Isoform Number
up
stable
down
UP
n=714
DOWN
n=1189
UP
n=1102
DOWN
n=1699

 © The Author(s)

Figure EV5. Full-length transcriptome sequence quality control and differentially expressed genes or isoforms.

(**A**) Statistics on the proportion of known transcripts in each sample. (**B**) Gene or isoform number of 1–2-cell embryos treated with THZ531 or DMSO. $n = 3$ biological replicates. Error bars, SEM. Exact *P* values are indicated directly on the figure. Significance was determined by one-way ANOVA followed by Tukey's test. (**C**) Analyses of the correlations between the indicated biological replicates of 1-cell and 2-cell embryos treated with DMSO or THZ531. (**D**) Transcriptome-based PCA of embryos treated with THZ531 or DMSO. (**E**) Differentially expressed gene (DEG) numbers in mouse early embryos at different stages. DEGs and isoforms (**G**) were identified using the limma package (moderated t-test) with Benjamini–Hochberg correction for multiple comparisons; significance was defined as FDR <0.05 and |log2(fold change)| >2 or <0.5. CPM counts per million mapped reads. (**F**) StreamGraph shows the sources of up- and down-regulated genes or isoforms (**H**). The black number represents the number of genes or isoforms (**H**).

© The Author(s)
