# Supplementary material for: ZC3H14 phosphorylation is required for nuclear speckle assembly during zygotic splicing activation in mice
Source: EMBO Rep. 2026 Oct 7;27(21):6396–424. doi: 10.1038/s44319-026-00950-x (PMC13743749; doi:10.1038/s44319-026-00950-x)
Supplement: Supplementary file 2 [file 44319_2026_950_MOESM3_ESM.pdf]

EMBO reports

ZC3H14 Phosphorylation is Required for Nuclear Speckle Assembly During Zygotic Splicing Activation in Mice

Yang Wang, Hua Zhang, Zhe-Wei Hu, Dan Su, Jing Zhao, Xiao-Ying Fan, Li-Ling Liu, and Heng-Yu Fan

Corresponding author(s): Heng-Yu Fan (hyfan@zju.edu.cn) , Xiao-Ying Fan (Fan_xiaoying@gzlab.ac.cn), Li-Ling Liu (llliu@gxams.org.cn)

Review Timeline:

Submission Date:	2nd Mar 26
Editorial Decision:	18th May 26
Revision Received:	20th Jul 26
Editorial Decision:	31st Aug 26
Revision Received:	2nd Sep 26
Accepted:	10th Sep 26

Editor: Esther Schnapp / Martina Rembold

Transaction Report:

(Note: With the exception of the correction of typographical or spelling errors that could be a source of ambiguity, letters and reports are not edited. Depending on transfer agreements, referee reports obtained elsewhere may or may not be included in this compilation. Referee reports are anonymous unless the Referee chooses to sign their reports.)

1st Editorial Decision **18th May 2026**

Dear Dr. Fan,

Thank you for the submission of your manuscript to EMBO reports and for your revision plan that outlines how you want to address the referee comments that are again pasted below.

I agree with your suggestions for how to revise your ms and would like to invite you to revise it along the lines you suggest. I also agree that exploring potential compensatory mechanisms for ZC3H14 loss in somatic cells is not a requirement for EMBO reports.

Please note that the referee concerns must be fully addressed and their suggestions taken on board. Please address all referee concerns in a complete point-by-point response. Acceptance of the manuscript will depend on a positive outcome of a second round of review. It is EMBO reports policy to allow a single round of major revision only and acceptance or rejection of the manuscript will therefore depend on the completeness of your responses included in the next, final version of the manuscript.

We realize that it is difficult to revise to a specific deadline. In the interest of protecting the conceptual advance provided by the work, we recommend a revision within 3 months (18th Aug 2026). Please discuss the revision progress ahead of this time with the editor if you require more time to complete the revisions.

IMPORTANT NOTE: we perform an initial quality control of all revised manuscripts before re-review. Your manuscript will FAIL this control and the handling will be DELAYED if the following APPLIES:
1) A data availability section providing access to data deposited in public databases is missing. If you have not deposited any data, please add a sentence to the data availability section that explains that.
2) Your manuscript contains statistics and error bars based on n=2. Please use scatter blots in these cases. No statistics should be calculated if n=2.

When submitting your revised manuscript, please review the submission guidelines (https://link.springer.com/journal/44319/submission-guidelines#cms-Revised-submissions) and carefully review the instructions that follow below. Failure to include requested items will delay the evaluation of your revision.

1) a .docx formatted version of the manuscript text (including legends for main figures, EV figures and tables). Please make sure that the changes are highlighted to be clearly visible.

2) individual production quality figure files as .eps, .tif, .jpg (one file per figure). See hhttps://media.springernature.com/original/springer-cms/rest/v1/content/27825798/data/v1 for more info on how to prepare your figures.

3) We replaced Supplementary Information with Expanded View (EV) Figures and Tables that are collapsible/expandable online. A maximum of 5 EV Figures can be typeset. EV Figures should be cited as 'Figure EV1, Figure EV2" etc... in the text and their respective legends should be included in the main text after the legends of regular figures.

- For the figures that you do NOT wish to display as Expanded View figures, they should be bundled together with their legends in a single PDF file called *Appendix*, which should start with a short Table of Content. Appendix figures should be referred to in the main text as: "Appendix Figure S1, Appendix Figure S2" etc. See 'Expanded View' section of the submission guidelines.

- Additional Tables/Datasets should be labeled and referred to as Table EV1, Dataset EV1, etc. Legends have to be provided in a separate tab in case of .xls files. Alternatively, the legend can be supplied as a separate text file (README) and zipped together with the Table/Dataset file.

4) a .docx formatted letter INCLUDING the reviewers' reports and your detailed point-by-point responses to their comments. As part of the EMBO Press transparent editorial process, the point-by-point response is part of the Review Process File (RPF), which will be published alongside your paper.

5) a complete author checklist, which you can download from our author guidelines. Please insert information in the checklist that is also reflected in the manuscript. The completed author checklist will also be part of the RPF.

6) Please note that all corresponding authors are required to supply an ORCID ID for their name upon submission of a revised manuscript (<https://orcid.org/>), which can be linked to your profile in the manuscript submission system.

7) Before submitting your revision, primary datasets produced in this study need to be deposited in an appropriate public database. Please remember to provide a reviewer password if the datasets are not yet public. The accession numbers and database should be listed in a formal "Data Availability" section placed after Methods. Please note that the Data Availability Section is restricted to new primary data that are part of this study. * Note - All links should resolve to a page where the data can

be accessed. *

If your study has not produced novel datasets, please mention this fact in the Data Availability Section.

8) At EMBO Press we ask authors to provide source data for the main manuscript figures. You will receive a separate email with instructions for providing source data with your revised manuscript, including how to upload and organize the files.

9) Our journal also encourages inclusion of *data citations in the reference list* to directly cite datasets that were re-used and obtained from public databases. Data citations in the article text are distinct from normal bibliographical citations and should directly link to the database records from which the data can be accessed. In the main text, data citations are formatted as follows: "Data ref: Smith et al, 2001" or "Data ref: NCBI Sequence Read Archive PRJNA342805, 2017". In the Reference list, data citations must be labeled with "[DATASET]". A data reference must provide the database name, accession number/identifiers and a resolvable link to the landing page from which the data can be accessed at the end of the reference.

10) Regarding data quantification, the following points must be specified in each figure legend:

- the name of the statistical test used to generate error bars and P values,

- the number (n) of independent experiments (please specify technical or biological replicates) underlying each data point,

- the nature of the bars and error bars (s.d., s.e.m.),

- If the data are obtained from n Program fragment delivered error ``Can't locate object method "less" via package "than" (perhaps you forgot to load "than"?) at //ejpvfs23/sites23b/embor_www/letters/embor_decision_revise_and_review.txt line 54.' 2, use scatter blots showing the individual data points.

Discussion of statistical methodology can be reported in the materials and methods section, but figure legends should contain a basic description of n, P and the test applied.

- Please also include scale bars in all microscopy images.

11) The journal requires a statement specifying whether or not authors have competing interests (defined as all potential or actual interests that could be perceived to influence the presentation or interpretation of an article). In case of competing interests, this must be specified in your disclosure statement.

12) All Materials and Methods need to be described in the main text using our 'Structured Methods' format, which is required for all research articles. According to this format, the Methods section includes a separate Reagents and Tools Table file (listing key reagents, experimental models, software and relevant equipment and including their sources and relevant identifiers) and a Methods and Protocols section describing the methods using a step-by-step protocol format. The aim is to facilitate adoption of the methodologies across labs. More information on how to adhere to this format as well as a downloadable template (.docx) for the Reagents and Tools Table can be found in our author guidelines.

We would also welcome the submission of cover suggestions, or motifs to be used by our Graphics Illustrator in designing a cover.

As part of the EMBO publication's Transparent Editorial Process, EMBO reports publishes online a Review Process File (RPF) to accompany accepted manuscripts. This File will be published in conjunction with your paper and will include the referee reports, your point-by-point response and all pertinent correspondence relating to the manuscript.

You are able to opt out of this by letting the editorial office know (emboreports@embo.org). If you do opt out, the Review Process File link will point to the following statement: "No Review Process File is available with this article, as the authors have chosen not to make the review process public in this case."

I look forward to seeing a revised form of your manuscript when it is ready.

Kind regards,
Esther

Esther Schnapp, PhD
Senior Editor
EMBO reports

Referee #1:

This study systematically investigates the role of the RNA-binding protein ZC3H14 in early mouse embryonic development, with a particular focus on its function during zygotic splicing activation (ZSA). Through a combination of genetic knockout, phospho-mutant analyses, and pharmacological inhibition, the authors propose that CDK13-mediated phosphorylation of ZC3H14 promotes de novo nuclear speckle assembly, thereby ensuring proper RNA splicing. Transcriptomic and full-length RNA sequencing analyses further demonstrate that loss of ZC3H14 function leads to widespread splicing defects and accumulation of premature transcripts (ptRNAs), ultimately resulting in developmental arrest at the 2-cell stage. Overall, this study provides valuable insights into post-transcriptional regulation and nuclear organization during the maternal-to-zygotic transition. Nonetheless, a number of concerns remain that should be further clarified.

major concerns：

1: Does Zc3h14 knockout affect the assembly and stability of the PAXT complex, and are the expression levels or subcellular localization of other core components altered accordingly?

2: The stronger co-localization of ZC3H14 with DAPI signals at the GO and 2-4 cell stages may reflect stage-specific chromatin states or transcriptional activity; is this dynamic localization directly linked to the timing of its function in regulating ZSA?

3: Is nuclear speckle assembly a causal and necessary driver of ZSA, or merely a correlative consequence of disrupted RNA processing, as current evidence appears insufficient to establish causality?

4: In Fig. 1i, it is unclear whether the residual green signal in Zc3h14 knockout oocytes represents background fluorescence or remaining protein expression

5: Although Zc3h14 knockout embryos are described as arrested at the 2-cell stage, some embryos appear to progress to the 4-cell stage in the images; a quantitative analysis of developmental stage distribution would strengthen this conclusion, and similar quantification is needed for Fig. 3A-B.

6: There appears to be a discrepancy between reduced oocyte numbers in Zc3h14 knockout mice and the lack of effect of THZ531 on oocyte maturation.

7: In the THZ531-treated transcriptome, are both totipotency- and pluripotency-associated genes upregulated at the 1-cell stage and downregulated at the 2-cell stage; a systematic analysis of key markers such as Dux and Zscan4 would provide clearer insight.

8: Among the genes downregulated by both THZ531 and pladienolide B, which key genes are shared, and what are their developmental stage-specific expression patterns, particularly for canonical 2-cell genes?

9: Does THZ531-induced splicing inhibition exhibit any preferential effect on totipotency-associated transcripts versus pluripotency-associated transcripts?

10: What are the key splicing events regulated by ZC3H14, and are totipotency-associated transcripts selectively affected? Has the splicing of 2-cell-specific genes been systematically analyzed? Does ZC3H14 preferentially bind and regulate MERVL or other repeat-derived transcripts?

Minor concerns：

1: In Fig. 3A-B, it should be clarified whether mRNA injection experiments were performed in wild-type or knockout embryos to accurately interpret the functional outcomes.

2: In Fig. 1i, the nature of the residual fluorescence signal in knockout oocytes should be clarified, ideally with additional validation to confirm complete protein depletion.

3: Please clearly indicate the source of the data used. For example, which article the embryo data of plab treat is derived from.

Referee #2:

Reviewer Comments

Manuscript Title: ZC3H14 is Required for de novo Nuclear Speckle Assembly During Zygotic Splicing Activation in Mice.

Overall Assessment

This study focuses on the function and mechanism of ZC3H14 in regulating de novo assembly of nuclear speckles during zygotic splicing activation (ZSA) in mice. The manuscript features a complete experimental system, diverse technical approaches, and preliminary reveals the critical role of the CDK13-ZC3H14 in early embryonic development, with clear scientific novelty. However, the manuscript requires major revisions for re-evaluation, with minor revisions needed for further refinement.

I. Major Revisions

1. The manuscript clearly states that Zc3h14 systemic knockout mice are viable with normal development, and only exhibit reduced female fertility with embryonic arrest at the 2-cell stage. However, the core conclusion claims that ZC3H14 is a key regulator of de novo nuclear speckle assembly and global splicing activation during ZSA. If this protein is a core factor for RNA splicing and nuclear speckle assembly in eukaryotic cells, it cannot explain why somatic cells with Zc3h14 knockout develop and survive completely normally, while only embryos exhibit a lethal phenotype. This represents a fundamental logical contradiction in the manuscript. Please supplement experiments to identify isoform-specific expression of ZC3H14 between somatic cells and early embryos, and verify whether there is an embryo-specific functional isoform. Detect whether there is compensatory upregulation of homologous proteins or activation of alternative nuclear speckle assembly/splicing regulatory pathways in Zc3h14 knockout somatic cells, to explain the survival mechanism of somatic cells. If the above experiments cannot be completed, an objective and in-depth analysis of the potential mechanisms underlying this phenotypic contradiction should be included in the Discussion.

2. The core conclusion that "phosphorylation of ZC3H14 by CDK13 regulates its function" relies solely on treatment with the

inhibitor THZ531. However, THZ531 is a dual inhibitor of CDK12 and CDK13 with no significant subtype specificity, making it impossible to exclude the contribution of CDK12 inhibition to the observed phenotypes. Supplement an in vitro kinase assay: purify recombinant CDK13 kinase and wild-type/4SA mutant ZC3H14 proteins, and directly demonstrate that CDK13 phosphorylates ZC3H14 with the four serine residues as core phosphorylation sites, via isotope labeling or phospho-specific antibody detection. Cdk13-specific siRNA/shRNA knockdown experiments as a parallel validation of pharmacological inhibition, to detect the phosphorylation level of ZC3H14, nuclear speckle assembly, and embryonic developmental phenotypes, and compare with the results of THZ531 treatment.

3. The manuscript draws the conclusion of "maternal-specific function of ZC3H14" based on the 2-cell arrest phenotype of embryos from Zc3h14 systemic knockout female mice, with two critical flaws: (1) Systemic knockout female mice exhibit phenotypes of reduced ovarian volume and significantly decreased ovulation number, making it impossible to distinguish whether embryonic arrest is a direct effect of maternal ZC3H14 depletion in oocytes, or an indirect consequence of abnormal ovarian development and reduced oocyte quality. (2) The functional difference between maternally derived and zygotically derived ZC3H14 is not distinguished, and it cannot be confirmed whether zygotic ZC3H14 is not involved in early embryonic development.

To validate the maternal-specific function of ZC3H14, a mandatory maternal mRNA rescue experiment must be supplemented to confirm that embryonic arrest is directly caused by the loss of zygotic maternally supplied ZC3H14, rather than indirect effects of abnormal oocyte development from systemic knockout. A zygote-specific knockout experiment is also required to verify that zygotic ZC3H14 is dispensable for early embryonic development, which is the core criterion for a maternal effect factor.

4. For the core claim that "ZC3H14 is a key driver of de novo nuclear speckle assembly during ZSA", the manuscript has an incomplete evidence chain and unestablished causal relationship.

The spliceosome inhibitor pladienolide B (PlaB) directly inhibit global splicing activity, and detect whether ZC3H14 nuclear speckle assembly is affected at the 2-cell stage. This is the direct experiment to rule out reverse causality. It will clarify whether the loss of nuclear speckles is the cause of splicing defects, or a secondary consequence of impaired splicing.

Inhibit global zygotic transcription with α-amanitin at the 1-cell stage (prior to nuclear speckle assembly), and observe whether ZC3H14 nuclear speckles can form normally at the 2-cell stage. This experiment will clarify whether ZC3H14-mediated nuclear speckle assembly is dependent on nascent transcripts from zygotic genome activation (ZGA), which is directly related to the core scientific question of the manuscript.

II. Minor Revisions

1. Statistical method standardization: For multi-group comparison experiments (e.g., 3 or more treatment groups in Fig3A, 3F), multiple t-tests should be replaced with one-way analysis of variance (ANOVA) followed by post hoc multiple tests (e.g., Tukey's test). A comprehensive full-text review of the manuscript is recommended.

2. Data presentation standardization: All quantitative plots must clearly indicate the type of error bars (standard error of the mean (SEM)/standard deviation (SD)), and clearly define the meaning of the sample size n: specify whether n is the number of biological replicates (number of mice/independent experiments), technical replicates, or the number of embryos/nuclei counted, and maintain consistency throughout the manuscript.

3. Abbreviation unification: Unify the abbreviations of proper nouns throughout the manuscript: for example, the abbreviation plaB is given when pladienolide B first appears, and the abbreviation must be consistent throughout the main text and supplementary materials.

4. Terminology precision correction: "de novo nuclear speckle assembly" is recommended to be revised to "phosphorylation-dependent nuclear speckle reassembly", as nuclear speckles dynamically disassemble and reassemble during the cell cycle, rather than being completely "synthesized de novo"

5. References section correction: Correct the duplicate citation issue (Insco et al, 2023a/b are the same literature and are cited repeatedly). Check the author, year, and journal information of all citations to correct citation errors.

6. Experimental method details for reproducibility: Supplement the method for the precise volume and concentration of mRNA injection, the specific time point of injection (e.g., specific stage of the pronuclear phase).

7. Drug treatment experiment details: Supplement the experimental basis for the selection of drug concentrations of THZ531, α-amanitin, and plaB, the exact time point and duration of treatment. Supplement the detection of transcriptional activity (5-ethynyl uridine (EU) incorporation, RNA polymerase II phosphorylated at serine-2 (pS2) staining) under 5 μM THZ531 treatment to prove that global transcription is not affected at this concentration.

Referee #3:

The manuscript by Wang et al. investigates the role of the RNA-binding protein, ZC3H14, during zygotic splicing activation (ZSA) in oogenesis and early mammalian embryogenesis. In mammals, zygotic genome activation (ZGA) is a critical process facilitating the maternal-to-zygotic transition. In the mouse model, major ZGA is said to occur at the 2-cell stage, during which a substantial increase in transcription necessitates efficient RNA processing, including splicing, capping, and polyadenylation. Proper activation of zygotic splicing is essential for transcript maturation and export, and defects in this process can result in aberrant splicing, developmental abnormalities, or embryonic lethality. For instance, the splicing factor, SF3B1, has been implicated in this context. In the current study, the authors propose that ZC3H14 localizes to nuclear speckles during ZSA and that it facilitates recruitment of the PAXT complex, including PABPN1, in a phosphorylation-dependent manner. Using the CDK12/13 inhibitor, THZ531, they demonstrate that perturbation of ZC3H14 phosphorylation disrupts speckle assembly and leads to developmental arrest. Furthermore, using comparative transcriptomic analyses (Smart-seq) between THZ531-treated

embryos and publicly available RNA-seq datasets from 2-cell embryos treated with pladienolide B, the authors suggest that inhibition of ZC3H14 affects ZSA-associated transcript splicing.

While the study presents a comprehensive ZSA spliced transcriptome dataset related to early embryonic development, its novelty appears limited. As acknowledged by the authors, several aspects of ZC3H14 biology-including its localization to nuclear speckles, involvement in RNA splicing, role in gametogenesis (particularly spermatogenesis), and phosphorylation by CDK13-have been previously reported in other systems. Thus, the scientific impact of the current work remains rather weak.

Major comments

The dataset presented is of potential interest to the field. However, the central claim regarding the requirement of ZC3H14 in ZSA is not sufficiently substantiated. A significant limitation is that many of the key experiments, including transcriptomic analyses, rely on treatment with THZ531. Given that CDK13 has multiple known phosphorylation targets beyond ZC3H14-including components of the translation machinery such as 4E-BP1 and eIF4B, as well as RNA processing factors like RBM39 and ASF/SF2-the observed phenotypes cannot be attributed specifically to ZC3H14 function. Consequently, the immunofluorescence and Smart-seq data derived from THZ531-treated embryos provide only indirect evidence and do not convincingly demonstrate a direct role for ZC3H14 in splicing regulation.

Additionally, the discrepancy between results describing phenotype of Zc3h14 knockout mice and the developmental arrest observed upon THZ531 treatment is not adequately addressed. This inconsistency weakens the overall argument and raises concerns about the specificity of the conclusions.
Several data interpretations require further clarification. For example, in Figure 1D, speckled localization of ZC3H14 is also observed at the 1-cell stage (PN3 and PN5), yet this observation is not sufficiently discussed. This is particularly relevant considering recent studies reporting that embryonic genome activation starts at the 1-cell stage across different mammalian species (e.g., DOI: 10.1073/pnas.2526998123; 10.1016/j.celrep.2023.112023; 10.1016/j.stem.2021.11.012), which are not cited in the manuscript.

Additional major points include (italic sentence are quote from the manuscript):

•*line 191: It indicates that ZC3H14 plays an extremely minor role in the maturation of oocytes.*
A recent report by Deng et al. (Nucleic Acid Research, 2026) indicates that ZC3H14 is part of the protein network involved in oocyte maturation, alongside other factors such as TDP43, which is critical for nuclear speckle assembly.
Additionally, multiple previous references indicate the conserved function of ZC3H14 in regulating RNA stability, making its regulation crucial for developmental stages requiring intensive RNA processing, such as oocyte meiotic maturation.
https://doi.org/10.1093/hmg/ddx248
Please explain/discuss the discrepancy of the conclusions.

• line 223: *IF results showed that during the transition from the 1-cell to the 2-cell stage, THZ531 treatment led to a reduction or complete disappearance of nuclear speckles in endogenous ZC3H14 cells compared to the control group (Fig. 4E)*.
Please add the IF data of the 1-cell stage to support the claim.

• line 262: *Principal component analysis (PCA) showed no significant differences between the experimental and control 1-cell embryos, whereas the 2-cell embryos showed clear distinctions (Fig. 6A).*
Fig 6 A: PCA shows a clear difference in clustering between control and experimental 1-cell embryos. Please revise the l.262 statement and expand the discussion to explain this.

• line 339: *To study the effects of ptRNA accumulation on early mouse embryonic development, we constructed Ddx39b-ptRNA with the last four exons deleted at the 3' end and overexpressed it in mouse zygotes via microinjection. The control group was microinjected with GFP mRNA.*
What would be the developmental effect of full-length Ddx39b-RNA?

Minor comments
The manuscript would benefit from more careful editing to ensure consistency in reference formatting. Additionally, multiple typographical errors and duplications are present in the reference list and should be corrected.

Fig S1B scale bar is missing

Fig S2 legend says immunofluorescence staining, however the main text says "*.....overexpression of GFP-ZC3H14 in oocytes and early embryos confirmed these observations (Fig. S2)*". Please clarify what it is that is being referred to in each and how they relate to each other.

Re: EMBOR-2026-63958V1
Point-by-point responses to reviewers' comments

Referee 1:
This study systematically investigates the role of the RNA-binding protein ZC3H14 in early mouse embryonic development, with a particular focus on its function during zygotic splicing activation (ZSA). Through a combination of genetic knockout, phospho-mutant analyses, and pharmacological inhibition, the authors propose that CDK13-mediated phosphorylation of ZC3H14 promotes de novo nuclear speckle assembly, thereby ensuring proper RNA splicing. Transcriptomic and full-length RNA sequencing analyses further demonstrate that loss of ZC3H14 function leads to widespread splicing defects and accumulation of premature transcripts (ptRNAs), ultimately resulting in developmental arrest at the 2-cell stage. Overall, this study provides valuable insights into post-transcriptional regulation and nuclear organization during the maternal-to-zygotic transition. Nonetheless, a number of concerns remain that should be further clarified.

Response: We sincerely thank Referee 1 for the thorough and insightful evaluation of our manuscript, and for clearly articulating the core issues that need to be addressed. We greatly appreciate the constructive suggestions, which have helped us recognize the key weaknesses in establishing causality and specificity. We have carefully considered all the concerns raised and are fully committed to addressing each of them. Below we have prepared detailed, point-by-point responses and revision plans.

major concerns：
1: Does *Zc3h14* knockout affect the assembly and stability of the PAXT complex, and are the expression levels or subcellular localization of other core components altered accordingly?

Response: This is an important point. We have already shown that ZC3H14 KO disrupts the nuclear speckle localization of PABPN1 (Fig. 5A). To more comprehensively address the PAXT complex, we examined the subcellular localization and protein expression levels of the other two core PAXT components, ZFC3H1 and MTR4, in WT versus *Zc3h14* KO oocytes. As shown in the figure below, both ZFC3H1 and MTR4 remained diffusely distributed throughout the nucleoplasm and showed no significant changes in protein expression levels in the absence of ZC3H14. These results indicate that the effect of *Zc3h14* knockout on the PAXT complex is limited to the nuclear speckle localization of PABPN1, and does not impact the overall stability or expression of the other core components.

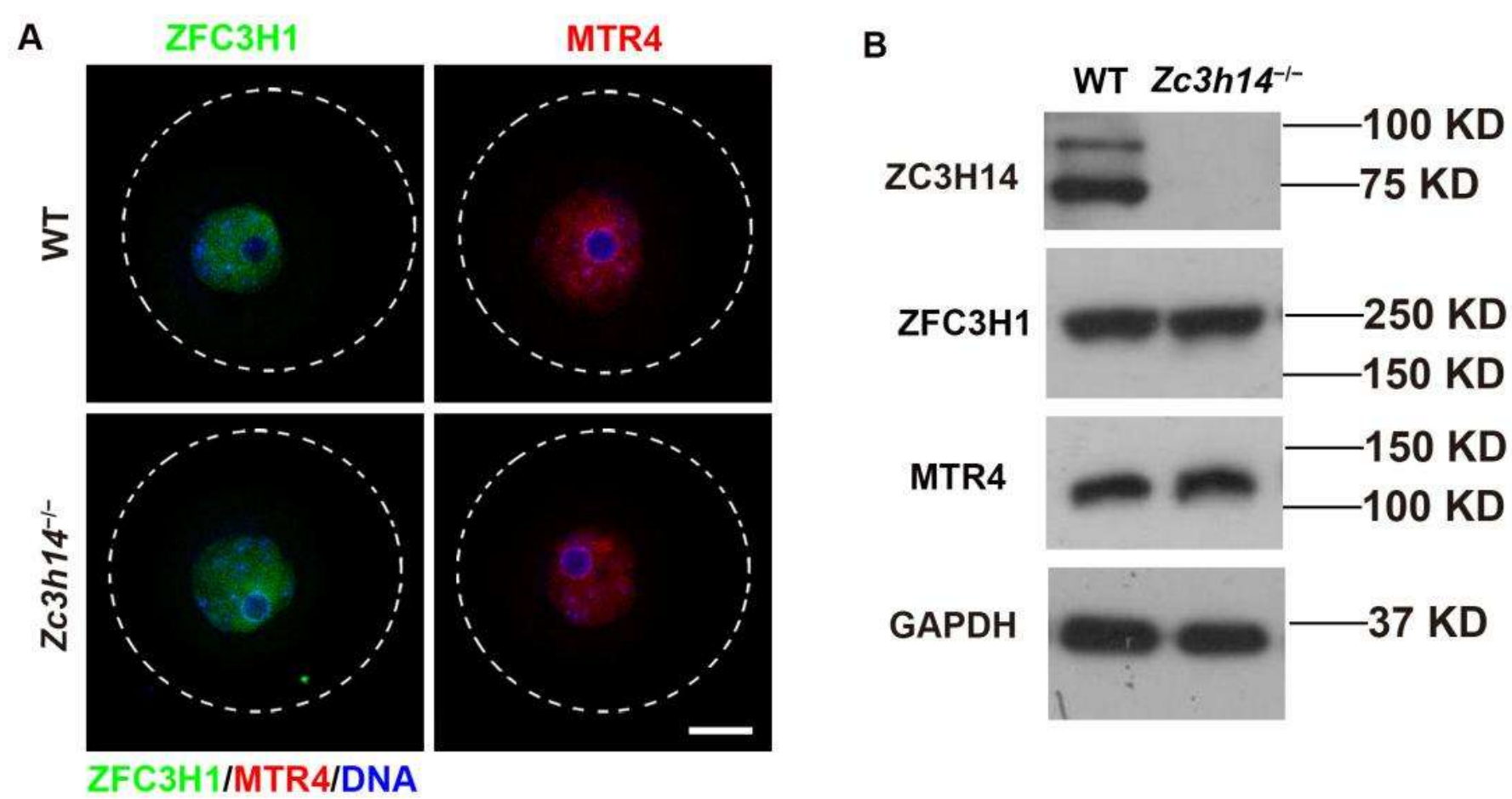

2: The stronger co-localization of ZC3H14 with DAPI signals at the GO and 2-4 cell stages may reflect stage-specific chromatin states or transcriptional activity; is this dynamic localization directly linked to the timing of its function in regulating ZSA?

Response: We thank the reviewer for this insightful observation. We agree that the colocalization with DAPI-low regions (active chromatin) is consistent with nuclear speckles being positioned near transcriptionally active sites. Our data show that ZC3H14 speckles form prominently during transcriptionally active stages (GO, 2-cell) and are diffuse in silent stages (FGO, MII). Notably, weak and sparse speckles begin to appear as early as the PN5 stage, coinciding with the onset of minor ZGA, and their number and size increase dramatically at the 2-cell stage when major ZGA occurs (revised Fig. 1A). Importantly, the α-amanitin experiment (new Fig. EV3E) shows that active transcription is not required for speckle formation *per se*. This suggests a model where the *competence* for ZC3H14 to form speckles is gained through stage-specific CDK12/13-mediated phosphorylation, and their positioning near active chromatin is likely important for efficient co-transcriptional splicing. We have added this discussion to the revised manuscript.

3: Is nuclear speckle assembly a causal and necessary driver of ZSA, or merely a correlative consequence of disrupted RNA processing, as current evidence appears insufficient to establish causality?

Response: This is indeed the core issue. In our original submission, we provided multiple lines of evidence supporting a causal role: in Zc3h14 KO oocytes, speckle markers SRSF2 and PABPN1 are lost, and reintroduction of WT, but not 4SA, ZC3H14 restores them (Fig. 5A-B). The 4SA phospho-dead mutant also fails to form speckles and causes developmental arrest, while the 4SD phospho-mimetic partially rescues THZ531-induced defects (Fig. 3F-G, Fig. 5E). Additionally, THZ531 treatment reduces nuclear speckles of ZC3H14 and its co-localized proteins (Fig. EV4A-C). To more directly address the concern of reverse causality, we treated 1-cell embryos with the splicing inhibitor PlaB and found that ZC3H14 nuclear speckles can still assemble at the 2-cell stage under PlaB treatment, although the number of speckles is reduced (new Fig. EV3G). This indicates that speckle assembly is not merely a downstream consequence of global splicing activity. Furthermore, inhibiting transcription with α-amanitin does not prevent ZC3H14 speckle formation (new Fig. EV3E), ruling out dependence on nascent ZGA transcripts. Overall, these

results support a model in which CDK12/13-mediated phosphorylation of ZC3H14 drives speckle reassembly upstream of efficient ZSA, while also acknowledging that the relationship between speckle integrity and splicing activity is likely bidirectional and mutually reinforcing. We have restructured the Results and Discussion sections accordingly to present this causal chain more clearly.

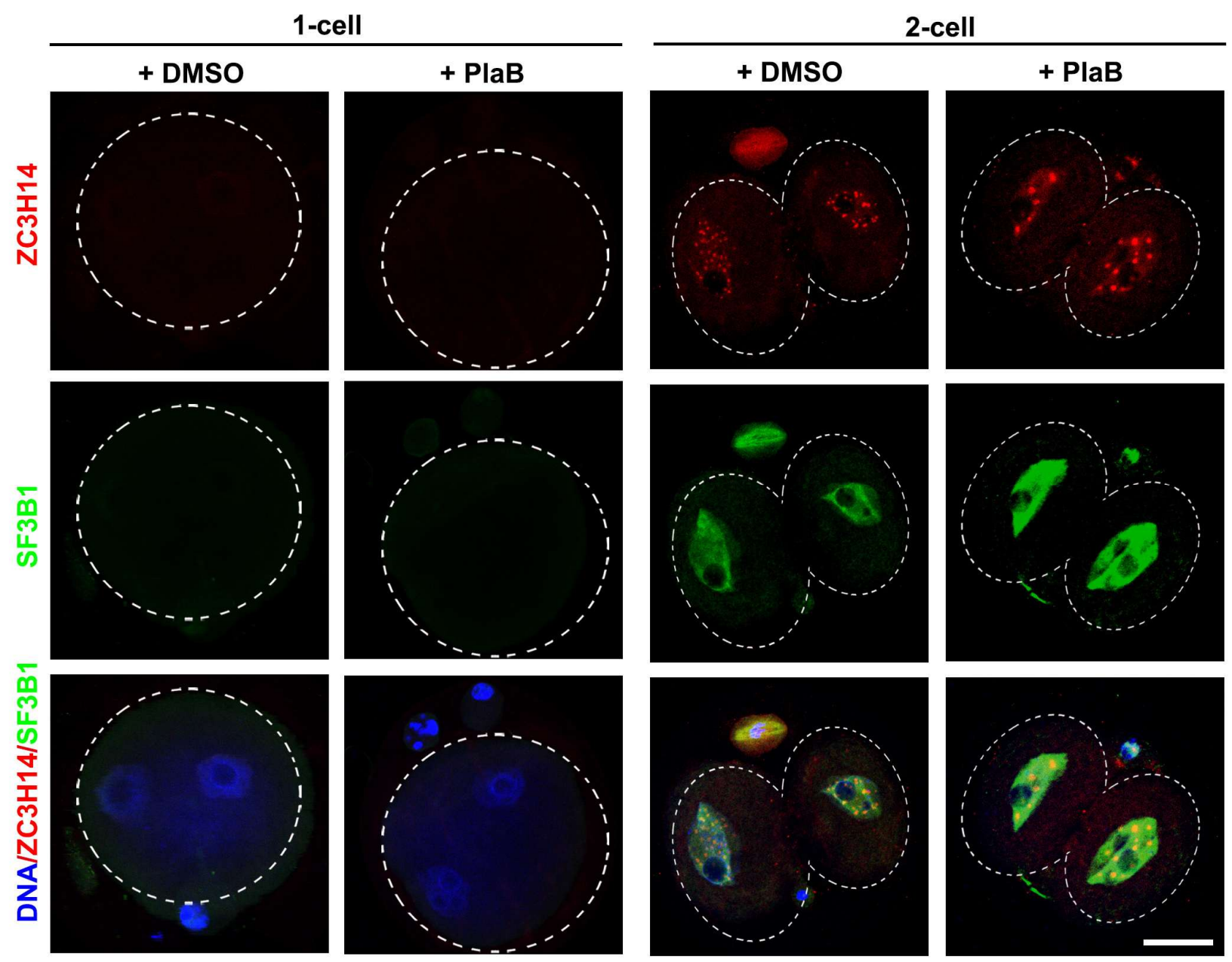

4: In Fig. 2i, it is unclear whether the residual green signal in Zc3h14 knockout oocytes represents background fluorescence or remaining protein expression

Response: We apologize for the lack of clarity. The green signal in original Fig. 2i is indeed background fluorescence that outlines the oocyte membrane and cytoplasm, which is present in both WT and KO samples and helps visualize cell morphology. This signal should not be interpreted as residual ZC3H14 protein, as Western blot analysis (Fig. 2G) clearly demonstrates the complete absence of ZC3H14 protein in the KO samples. To avoid any further confusion, we have revised the figure legend for Fig. 2i to explicitly state that the membrane/cytoplasmic signal represents background fluorescence, and we have cross-referenced the WB data confirming complete knockout.

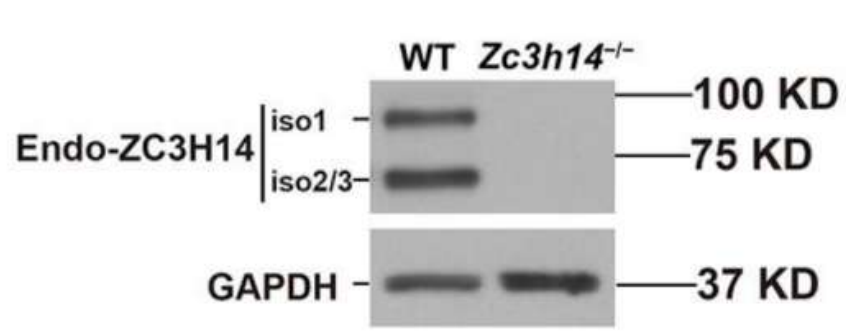

5: Although *Zc3h14* knockout embryos are described as arrested at the 2-cell stage, some embryos appear to progress to the 4-cell stage in the images; a quantitative analysis of developmental stage distribution would strengthen this conclusion, and similar quantification is needed for Fig. 3A-B.

Response: This is a valid point. We agree that a small percentage of *Zc3h14* KO embryos can occasionally cleave to the 4-cell stage; however, these embryos are morphologically abnormal and never progress to the blastocyst stage. To provide a more accurate and quantitative picture, we performed detailed stage-by-stage quantification for both the *Zc3h14* KO embryos (revised Fig. 2J) and the 4SA mutant-injected embryos (revised Fig. 3A). These new quantifications show the proportions of embryos at the 2-cell, 4-cell, 8-cell, morula, and blastocyst stages, with statistical significance indicated. The revised figures are included in the response document. We have also replaced multiple t-tests with two-way ANOVA followed by multiple comparisons tests for these multi-stage analyses, as recommended by the reviewer

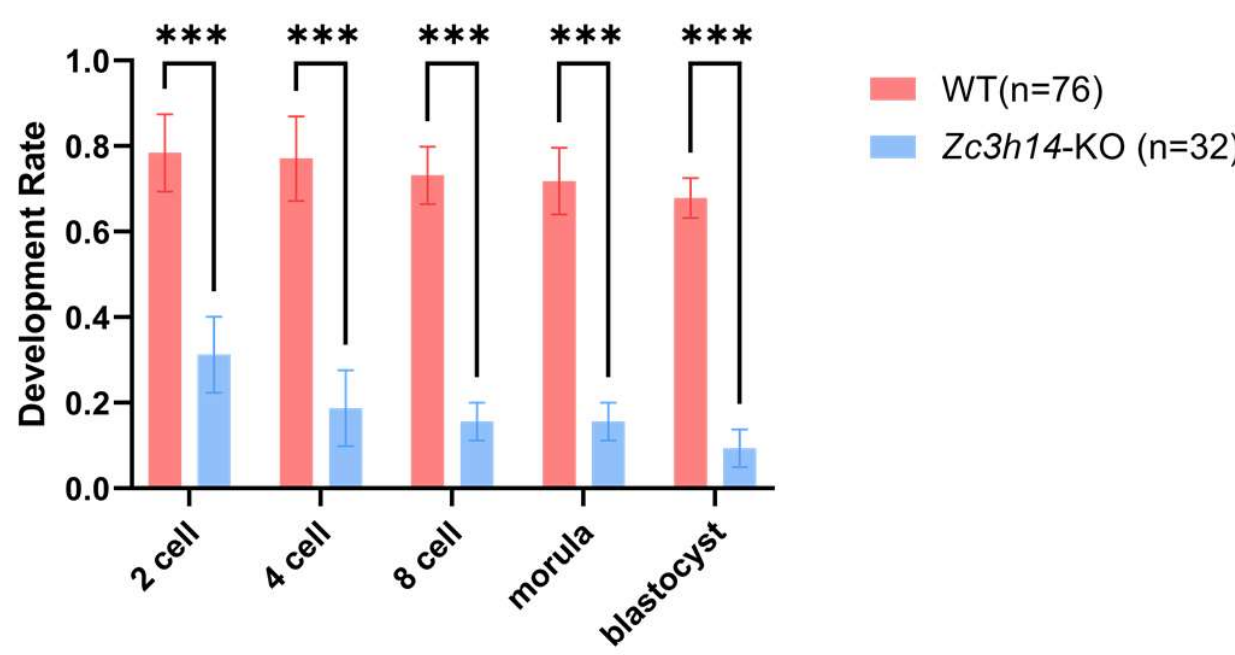

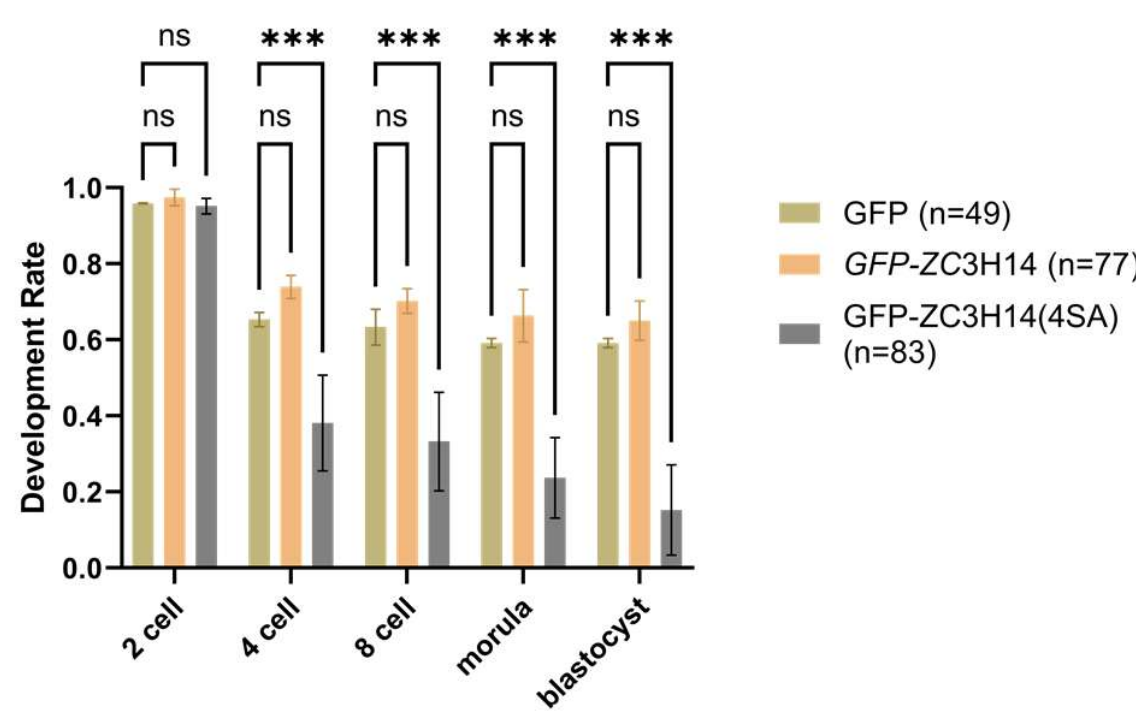

6: There appears to be a discrepancy between reduced oocyte numbers in *Zc3h14* knockout mice and the lack of effect of THZ531 on oocyte maturation.

Response: This point is well taken. We do not view this as a discrepancy, but rather as a reflection of fundamentally different experimental timescales and physiological contexts. The systemic *Zc3h14* knockout affects oocyte development over a prolonged period—from primordial follicle formation through to ovulation—which may lead to reduced oocyte numbers due to cumulative defects in folliculogenesis or oocyte survival. In contrast, THZ531 treatment is acute (a few hours during *in vitro* maturation), affecting only the final meiotic maturation steps of fully-grown oocytes that have already completed their growth phase. ZC3H14 may have redundant or compensatory functions during this short window, consistent with a recent network analysis (Deng et al., NAR, 2026) that predicted its involvement without functional validation. Importantly, the THZ531 treatment data demonstrate that ZC3H14 phosphorylation is dispensable for meiotic progression under these experimental conditions, while the KO phenotype reveals its essential role during the earlier stages of oogenesis and the subsequent embryonic development. We have added an explicit discussion of this distinction in the revised manuscript to avoid any potential

misinterpretation.

7: In the THZ531-treated transcriptome, are both totipotency- and pluripotency-associated genes upregulated at the 1-cell stage and downregulated at the 2-cell stage; a systematic analysis of key markers such as Dux and Zscan4 would provide clearer insight.

Response: We thank the reviewer for this critical suggestion. We have reanalyzed our RNA-seq data as requested and the results are now presented in new Fig. EV2A-C. Our analysis shows that, compared to DMSO-treated controls, THZ531 treatment leads to little change in the expression of totipotency- or pluripotency-associated genes at the 1-cell stage (Fig. EV2A-B). At the 2-cell stage, however, THZ531 treatment significantly downregulates key totipotency markers, including the *Zscan4* family and MERVL, as well as pluripotency markers such as *Nanog* and *Sox2* (Fig. EV2C). Interestingly, we observed that upstream activators like *Dux* show a trend towards upregulation at the 2-cell stage, which may represent a compensatory response to insufficient ZGA. These findings suggest that THZ531-mediated splicing inhibition impairs the normal upregulation of both totipotency and pluripotency programs during the 2-cell stage, thereby hindering the transition from totipotency to pluripotency. We have added a detailed description of these results in the Results section of the revised manuscript.

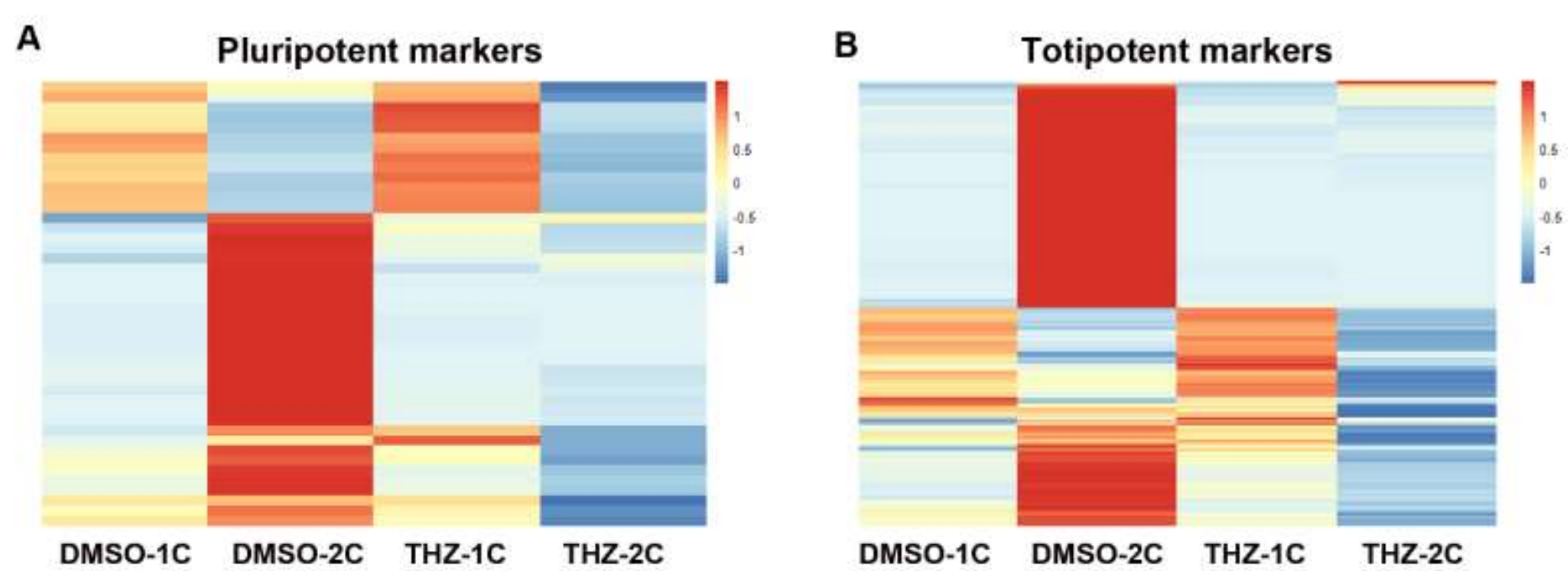

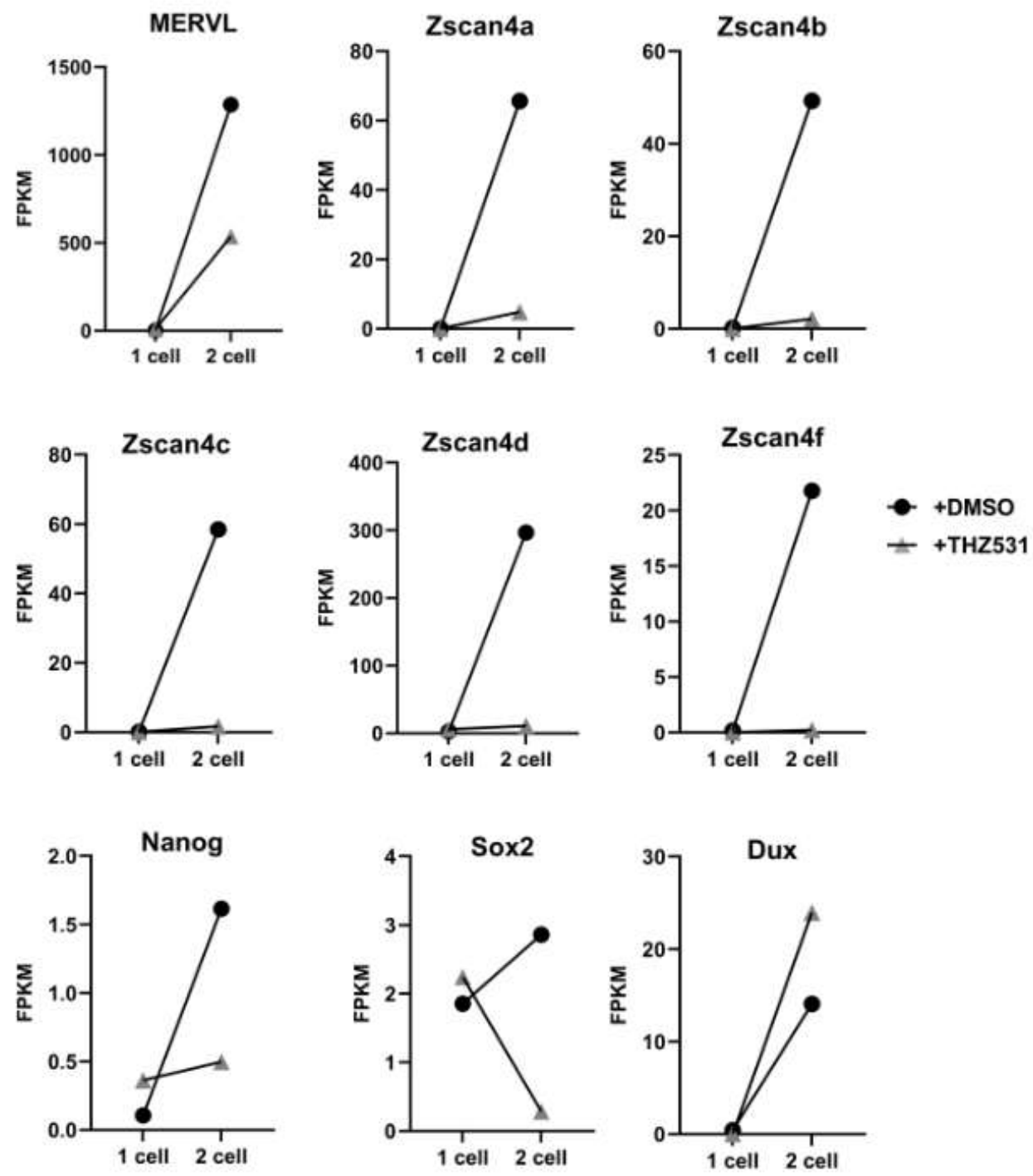

8: Among the genes downregulated by both THZ531 and pladienolide B, which key genes are shared, and what are their developmental stage-specific expression patterns, particularly for canonical 2-cell genes?

Response: We thank the reviewer for this question. We identified 1,050 genes that were commonly downregulated by both THZ531 and PlaB treatment at the 2-cell stage (Fig. 6E). As shown in the heatmap (included in the response document), these shared genes are predominantly those that are normally upregulated during the 1-cell to 2-cell transition, and their upregulation is significantly blunted by both treatments. This gene set includes many known 2-cell-specific genes, such as *Zscan4* family members, *MERVL* elements, and other genes associated with the totipotent 2-cell program.

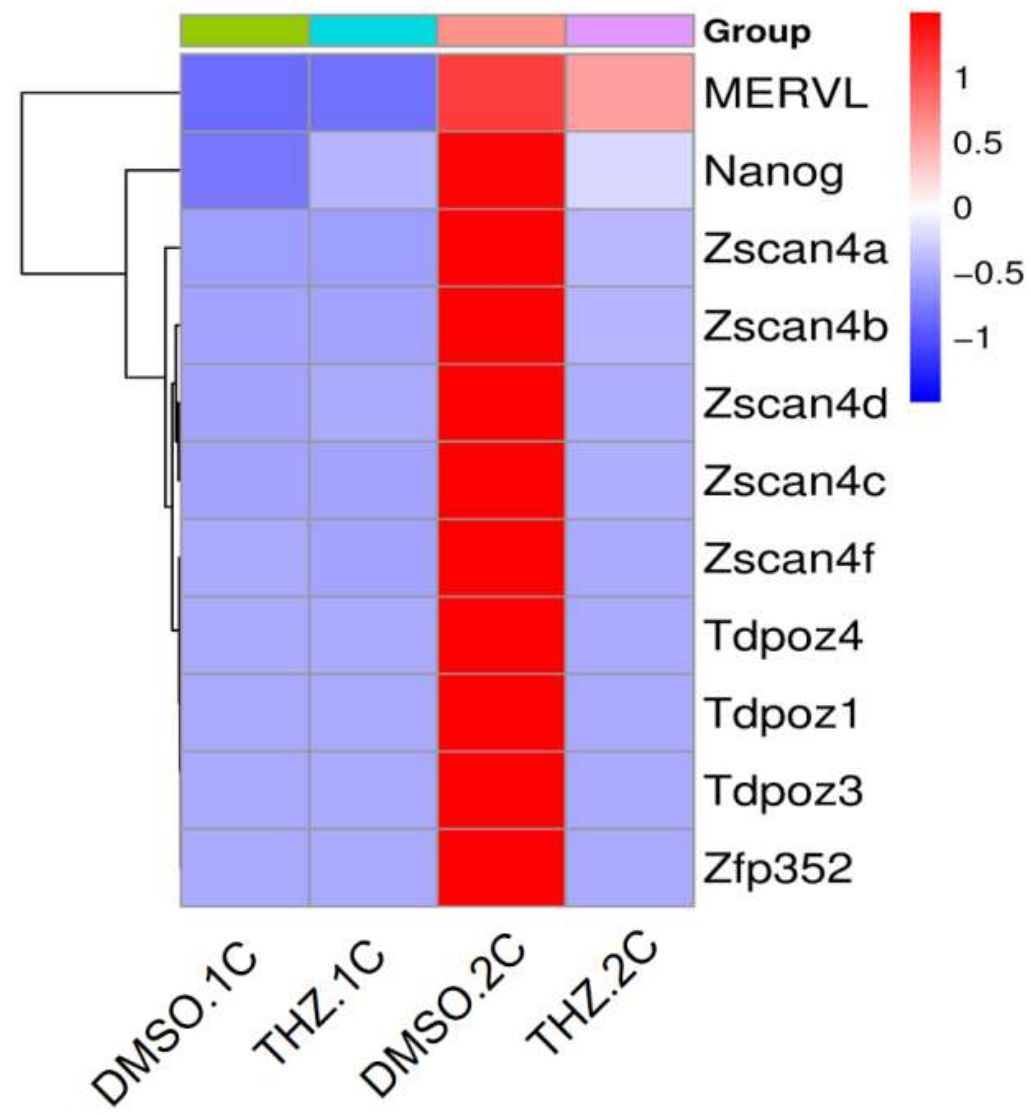

9: Does THZ531-induced splicing inhibition exhibit any preferential effect on totipotency-associated transcripts versus pluripotency-associated transcripts?

Response: We appreciate this question. Using rMATS analysis of our RNA-seq data, we compared the splicing changes induced by THZ531 treatment between totipotency-associated transcripts and pluripotency-associated transcripts. As shown in Fig. 8 (included in the response document), THZ531-induced splicing inhibition does not exhibit a statistically significant preference for transcripts related to totipotency over those related to pluripotency. The splicing defects induced by THZ531 appear to be widespread across the transcriptome, affecting both classes of genes to a similar extent.

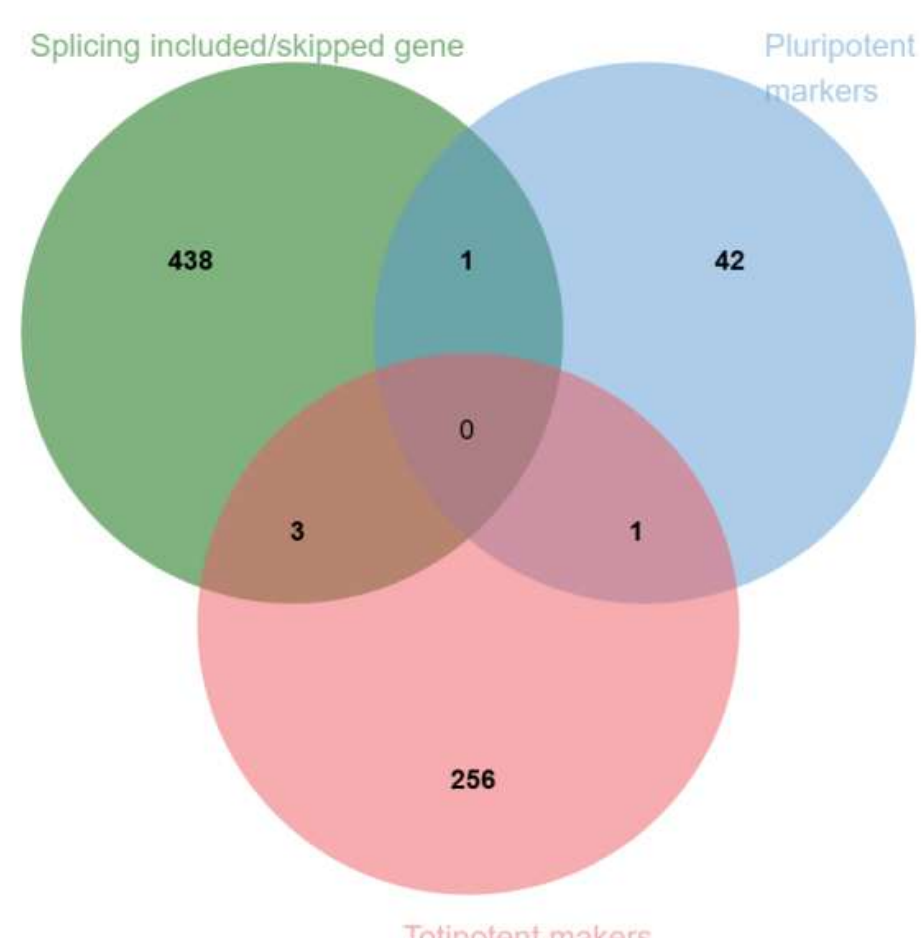

10: What are the key splicing events regulated by ZC3H14, and are totipotency-associated transcripts selectively affected? Has the splicing of 2-cell-specific genes been systematically analyzed? Does ZC3H14 preferentially bind and regulate MERVL or other repeat-derived transcripts?

Response: We thank the reviewer for raising these three important questions.

Key splicing events regulated by ZC3H14: Our rMATS analysis identifies intron retention (RI) and exon skipping (SE) as the most significantly affected alternative splicing events following THZ531 treatment (Fig. 6F). Specific examples of genes with altered splicing include *Ddx39b*, *Phgdh*, and *Sty17* (Fig. 7B, I), all of which exhibited increased intron retention or exon skipping at the 2-cell stage. A complete list of affected splicing events is provided in Supplementary Table EV3.

Selectivity on toti/pluri transcripts and 2-cell-specific genes: As addressed in our response to #9, THZ531-induced splicing inhibition does not show a statistically significant preference for totipotency-associated transcripts over pluripotency-associated transcripts. For 2-cell-specific genes, we have systematically analyzed their splicing patterns using rMATS and found that they are affected in a manner similar to the global transcriptome—namely, increased intron retention and exon skipping—without evidence of selective targeting. These results are now described in the Results section.

ZC3H14 binding to MERVL/repeat-derived transcripts: To directly test whether ZC3H14 binds MERVL or other repeat-derived transcripts, we performed RIP-qPCR experiments on lysates from wild-type 2-cell embryos using a ZC3H14 antibody. Our results show that ZC3H14 significantly enriches MERVL RNA compared to IgG control. Importantly, this enrichment is greatly diminished in the 4SA phosphorylation-deficient mutant, indicating that ZC3H14 phosphorylation is required for efficient binding to MERVL transcripts. We have included these data as a new supplementary figure (Fig. EV2D) and added a description in the Results section.

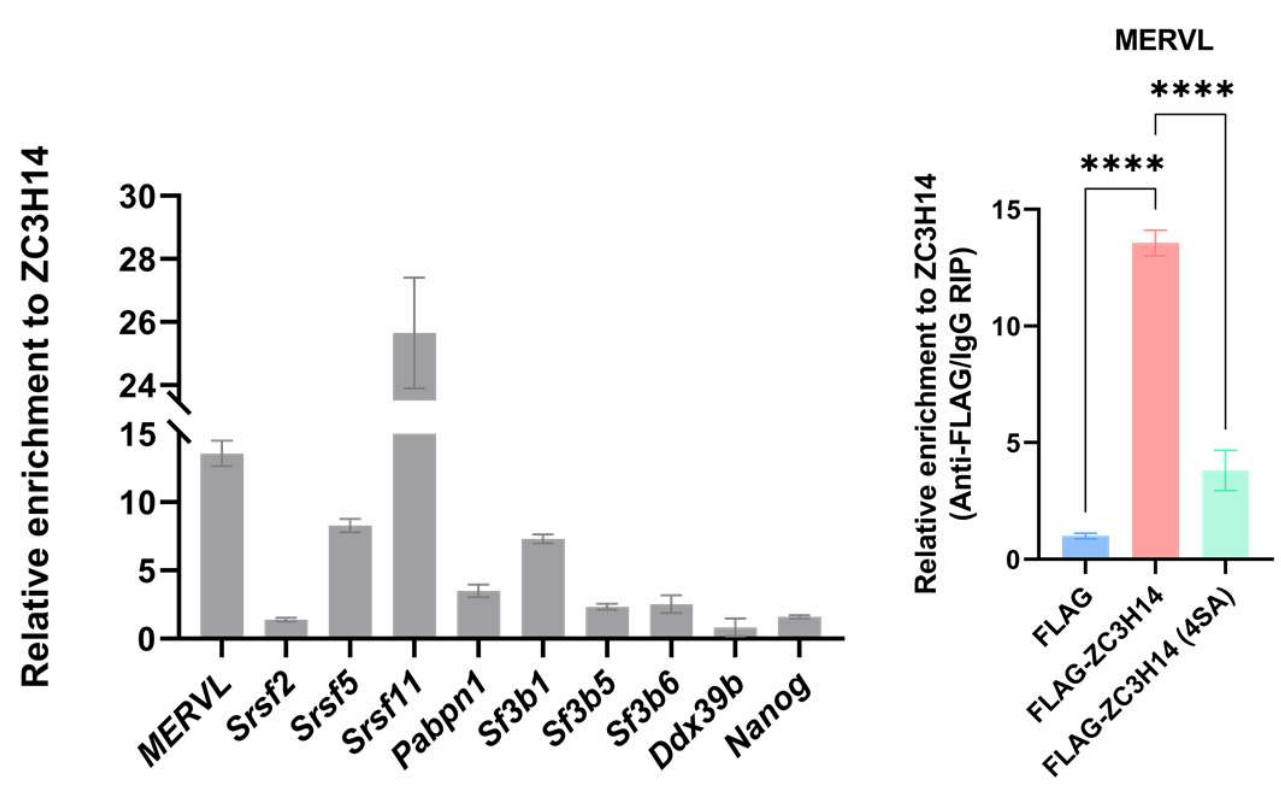

Minor concerns：

1: In Fig. 3A-B, it should be clarified whether mRNA injection experiments were performed in wild-type or knockout embryos to accurately interpret the functional outcomes.

Response: We apologize for the lack of clarity. The mRNA injection experiments shown in Fig. 3A-B were performed in **wild-type (WT)** zygotes. We have now explicitly stated this in the figure legend of the revised manuscript (Fig. 3A-B legend) to ensure accurate interpretation of the functional outcomes.

2: In Fig. 2i, the nature of the residual fluorescence signal in knockout oocytes should be clarified, ideally with additional validation to confirm complete protein depletion.

Response: As addressed in our response to Major Concern #4, the residual green signal in Fig. 2i

is background fluorescence that outlines the oocyte membrane and cytoplasm. We have now explicitly explained this in the revised figure legend for Fig. 2i and cross-referenced the Western blot data (Fig. 2G), which confirm the complete absence of ZC3H14 protein in the knockout samples.

3: Please clearly indicate the source of the data used. For example, which article the embryo data of plab treat is derived from.

Response: We apologize for not making this sufficiently clear. The PlaB-treated embryo RNA-seq data used in our analysis are from a previously published study (Zhang et al., *Advanced Science*, 2024, DOI: 10.1002/advs.202308496). This study is now cited in the "Data availability" section as well as in the main text (lines 72 and 752) of the revised manuscript. We have also included the NCBI accession number (PRJNA1004899) in the Data availability section to facilitate access to the public dataset.

Referee 2:

This study focuses on the function and mechanism of ZC3H14 in regulating de novo assembly of nuclear speckles during zygotic splicing activation (ZSA) in mice. The manuscript features a complete experimental system, diverse technical approaches, and preliminary reveals the critical role of the CDK13-ZC3H14 in early embryonic development, with clear scientific novelty. However, the manuscript requires major revisions for re-evaluation, with minor revisions needed for further refinement.

Response: We sincerely thank Referee 2 for the positive and encouraging evaluation of our experimental system and scientific novelty. We are also deeply grateful for the insightful and rigorous revision requests, which have been invaluable in strengthening our work and enhancing its overall quality.

I. Major Revisions

1. The manuscript clearly states that *Zc3h14* systemic knockout mice are viable with normal development, and only exhibit reduced female fertility with embryonic arrest at the 2-cell stage. However, the core conclusion claims that ZC3H14 is a key regulator of de novo nuclear speckle assembly and global splicing activation during ZSA. If this protein is a core factor for RNA splicing and nuclear speckle assembly in eukaryotic cells, it cannot explain why somatic cells with *Zc3h14* knockout develop and survive completely normally, while only embryos exhibit a lethal phenotype. This represents a fundamental logical contradiction in the manuscript. Please supplement experiments to identify isoform-specific expression of ZC3H14 between somatic cells and early embryos, and verify whether there is an embryo-specific functional isoform. Detect whether there is compensatory upregulation of homologous proteins or activation of alternative nuclear speckle assembly/splicing regulatory pathways in *Zc3h14* knockout somatic cells, to explain the survival mechanism of somatic cells. If the above experiments cannot be completed, an objective and in-depth analysis of the potential mechanisms underlying this phenotypic contradiction should be included in the Discussion.

Response: We thank the reviewer for raising this important point, as it allows us to clarify a potential misunderstanding. The reviewer's statement—*"If this protein is a core factor for RNA splicing and nuclear speckle assembly in eukaryotic cells"*—is **not a claim made in our manuscript**. Our study does **not** propose that ZC3H14 is a general, essential factor for RNA splicing or nuclear speckle assembly in all cell types. Rather, our conclusions are specifically contextualized to **zygotic splicing activation (ZSA) during the maternal-to-zygotic transition (MZT)** in early mouse embryos, and we do not extrapolate our findings to somatic cells.

In fact, the observation that *Zc3h14* systemic knockout mice are viable and develop normally (aside from reduced female fertility) **does not contradict our conclusions; rather, it reinforces the specificity of ZC3H14 function**. This phenotype indicates that ZC3H14 is not required for general cell viability or basic RNA processing in most somatic tissues, likely due to functional redundancy or alternative pathways that can be established during development. However, during the unique developmental window of ZSA—when the embryo must rapidly and accurately splice a massive wave of newly transcribed zygotic genes—the demand on the splicing machinery is extraordinary, and ZC3H14 becomes essential. This context-specific requirement is precisely why the knockout phenotype is restricted to early embryogenesis.

Furthermore, the developmental arrest observed in embryos from *Zc3h14* KO females is a

maternal effect: the embryos arrest because they lack the ZC3H14 protein deposited in the oocyte, not because the zygotic genome fails to express *Zc3h14*. This is fundamentally different from a somatic cell knockout, where cells have ample time to establish compensatory mechanisms during development. In the early embryo, there is no such opportunity before the critical ZSA window closes, and the phenotype is fully penetrant, with most embryos arresting during the 1-cell to 2-cell transition (Fig. 2J-K). Therefore, the "viable systemic KO" phenotype is fully consistent with a **maternal-effect, ZSA-specific** role for ZC3H14 and does not constitute a logical contradiction.

We fully agree with the reviewer that exploring potential compensatory mechanisms in somatic cells—such as upregulation of homologous proteins like ZC3H13 or activation of alternative splicing pathways—would be an interesting future direction. While these experiments are beyond the scope of the current study, we have added a clear discussion paragraph in the revised manuscript explicitly addressing the tissue-specific essentiality of ZC3H14 and the likely existence of compensatory mechanisms in somatic cells.

2. The core conclusion that "phosphorylation of ZC3H14 by CDK13 regulates its function" relies solely on treatment with the inhibitor THZ531. However, THZ531 is a dual inhibitor of CDK12 and CDK13 with no significant subtype specificity, making it impossible to exclude the contribution of CDK12 inhibition to the observed phenotypes. Supplement an in vitro kinase assay: purify recombinant CDK13 kinase and wild-type/4SA mutant ZC3H14 proteins, and directly demonstrate that CDK13 phosphorylates ZC3H14 with the four serine residues as core phosphorylation sites, via isotope labeling or phospho-specific antibody detection. Cdk13-specific siRNA/shRNA knockdown experiments as a parallel validation of pharmacological inhibition, to detect the phosphorylation level of ZC3H14, nuclear speckle assembly, and embryonic developmental phenotypes, and compare with the results of THZ531 treatment.

Response: We thank the reviewer for this thoughtful and technically rigorous comment. We acknowledge that THZ531 is a dual inhibitor of CDK12 and CDK13, and we cannot formally exclude the possibility that CDK12 also contributes to ZC3H14 phosphorylation *in vivo*. However, we would like to clarify the scope of our conclusion.

Our core finding is that phosphorylation of ZC3H14 on four specific serine residues is essential for its function in nuclear speckle reassembly and ZSA. Regarding the upstream kinase(s), the published *in vitro* kinase assay (Insco et al., *Science*, 2023) has already demonstrated that CDK13 can directly phosphorylate these sites. Whether CDK12 shares this capability or whether the two kinases function redundantly during early embryogenesis does **not** diminish the main conclusion of our study.

To provide genetic validation in parallel with the pharmacological inhibition, we carried out **Cdk13-specific siRNA knockdown experiments** in fertilized eggs. After knocking down CDK13 (new Fig. 3H), ZC3H14 nuclear speckles failed to assemble at the 2-cell stage (new Fig. 4E), and embryo development rates dropped significantly (new Fig. 3I-J), phenocopying the effects of THZ531 treatment. These results further support that CDK13 is a major kinase regulating ZC3H14 phosphorylation during ZSA.

To be fully accurate and to address the reviewer's concern, we have revised the wording throughout the manuscript to state that **"CDK12/13-mediated phosphorylation of ZC3H14"** is critical for ZSA, acknowledging the potential functional overlap between these two highly related

kinases. We have included the results of the siRNA experiments in the revised manuscript (new Fig. 3H-J and Fig. 4E).

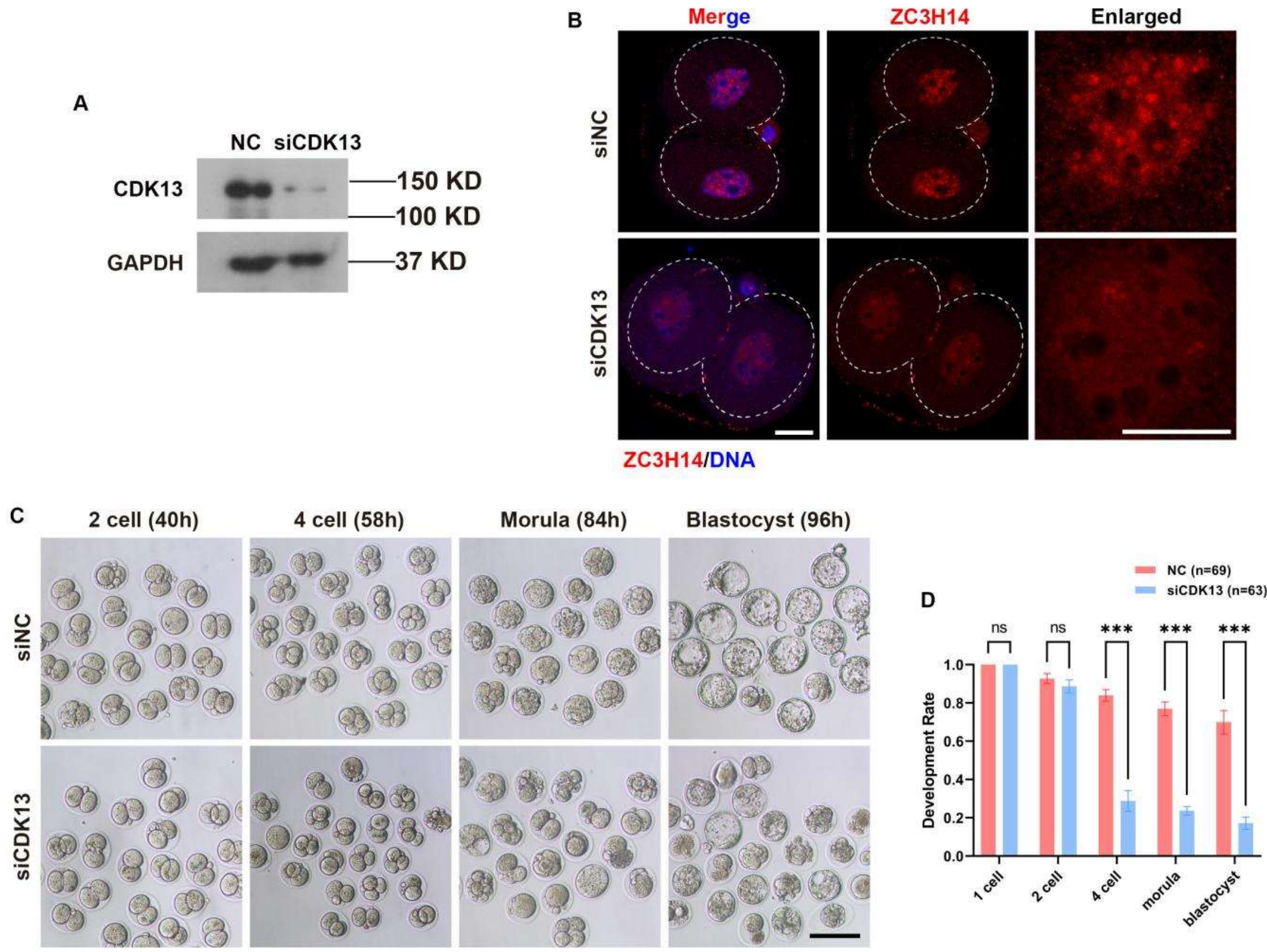

3. The manuscript draws the conclusion of "maternal-specific function of ZC3H14" based on the 2-cell arrest phenotype of embryos from *Zc3h14* systemic knockout female mice, with two critical flaws: (1) Systemic knockout female mice exhibit phenotypes of reduced ovarian volume and significantly decreased ovulation number, making it impossible to distinguish whether embryonic arrest is a direct effect of maternal ZC3H14 depletion in oocytes, or an indirect consequence of abnormal ovarian development and reduced oocyte quality. (2) The functional difference between maternally derived and zygotically derived ZC3H14 is not distinguished, and it cannot be confirmed whether zygotic ZC3H14 is not involved in early embryonic development.

To validate the maternal-specific function of ZC3H14, a mandatory maternal mRNA rescue experiment must be supplemented to confirm that embryonic arrest is directly caused by the loss of zygotic maternally supplied ZC3H14, rather than indirect effects of abnormal oocyte development from systemic knockout. A zygote-specific knockout experiment is also required to verify that zygotic ZC3H14 is dispensable for early embryonic development, which is the core criterion for a maternal effect factor.

Response: These are rigorous methodological points. They have helped us refine the distinction between maternal and zygotic contributions.

Maternal mRNA rescue experiment: To distinguish whether the 2-cell arrest is a direct effect of

maternal ZC3H14 depletion or an indirect consequence of abnormal ovarian development, we microinjected *in vitro* transcribed *Zc3h14* mRNA into *Zc3h14* KO MII oocytes, followed by *in vitro* fertilization (IVF). As shown in As shown in the picture below (included in the response document), this maternal mRNA rescue significantly restores embryonic development beyond the 2-cell stage, directly demonstrating that **the oocyte-supplied ZC3H14 is essential** and that the arrest is not merely an indirect consequence of ovarian abnormalities.

Zygotic ZC3H14 function: We agree with the reviewer that the contribution of zygotically expressed ZC3H14 remains to be formally tested. A zygote-specific knockout experiment—crossing *Zc3h14* heterozygous females with *Zc3h14* heterozygous males, collecting the resulting fertilized eggs (which include 25% *Zc3h14*-/- zygotic KO embryos), and culturing them *in vitro* to assess their development to the blastocyst stage—would provide definitive evidence on whether zygotic ZC3H14 is dispensable. We have proposed this experiment as a future direction in the revised Discussion.

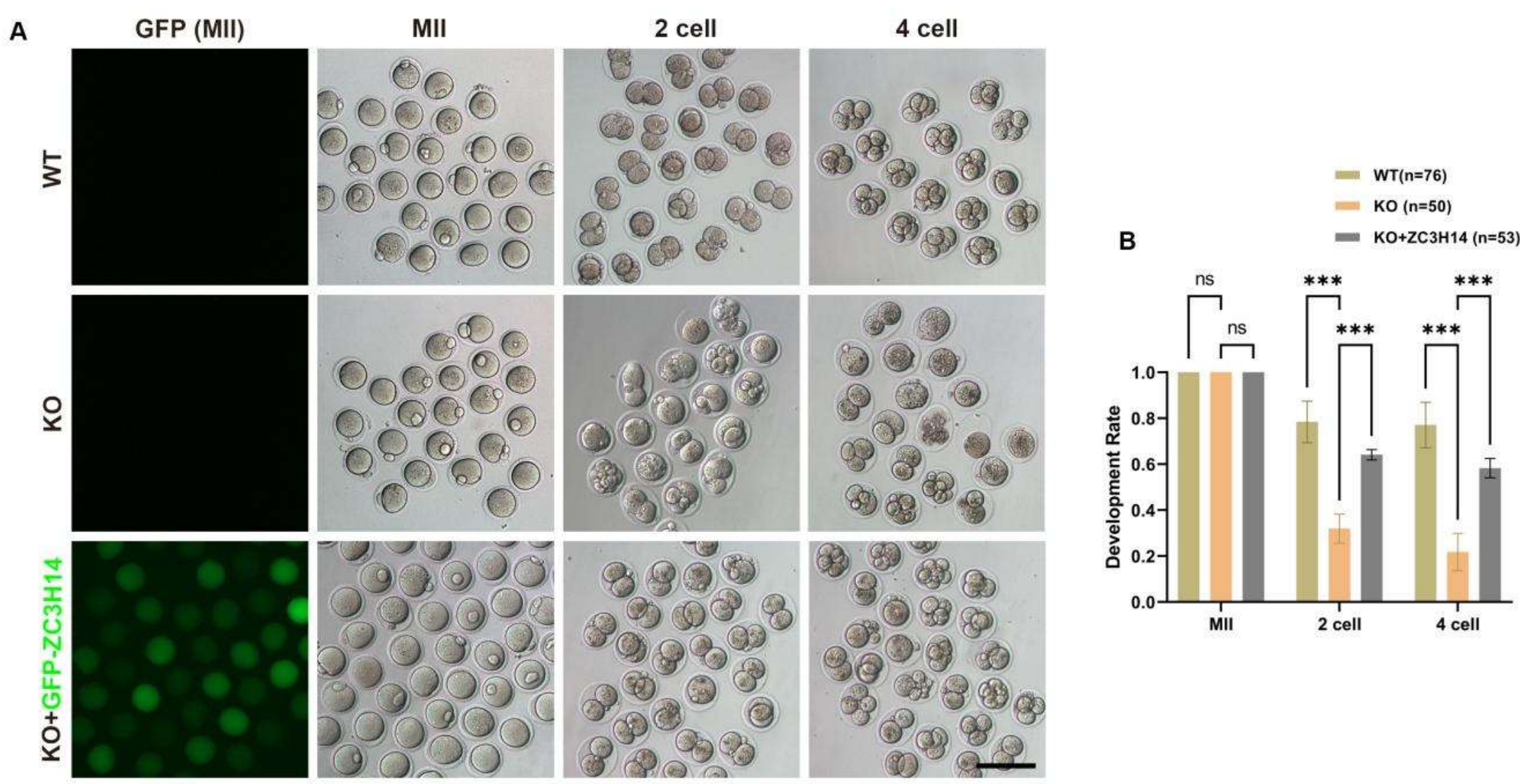

Independent evidence from inhibitor experiments: Importantly, the THZ531 treatment of WT zygotes already provides direct evidence that ZC3H14 phosphorylation is required in the zygote itself, independent of maternal contribution, because the inhibitor acts on the fertilized egg after fertilization. This pharmacological evidence, together with the 4SA/4SD phospho-mutant data, establishes that the phosphorylation-regulated function of ZC3H14 is required during the zygotic stage regardless of whether zygotic ZC3H14 protein is expressed.

We have revised our terminology throughout the manuscript to more precisely state that **maternally deposited ZC3H14 is essential for early embryogenesis**, while acknowledging that the relative contribution of zygotic ZC3H14 remains an open question for future investigation. This refined terminology is fully consistent with our response to Major Concern #1: the viable systemic KO mouse does not contradict our conclusions because the arrest phenotype is driven by the loss of oocyte-deposited ZC3H14, and the requirement for ZC3H14 phosphorylation during ZSA is independently validated by the inhibitor and phospho-mutant data.

4. For the core claim that "ZC3H14 is a key driver of de novo nuclear speckle assembly during ZSA", the manuscript has an incomplete evidence chain and unestablished causal relationship.

The spliceosome inhibitor pladienolide B (PlaB) directly inhibit global splicing activity, and detect whether ZC3H14 nuclear speckle assembly is affected at the 2-cell stage. This is the direct experiment to rule out reverse causality. It will clarify whether the loss of nuclear speckles is the cause of splicing defects, or a secondary consequence of impaired splicing.

Inhibit global zygotic transcription with α-amanitin at the 1-cell stage (prior to nuclear speckle assembly), and observe whether ZC3H14 nuclear speckles can form normally at the 2-cell stage. This experiment will clarify whether ZC3H14-mediated nuclear speckle assembly is dependent on nascent transcripts from zygotic genome activation (ZGA), which is directly related to the core scientific question of the manuscript.

Response: We agree with the reviewer that establishing causality is essential for our core claim. As addressed in our response to Referee 1, Major Concern #3, we performed the key experiments requested to address this issue.

To rule out reverse causation (i.e., whether speckle loss is a consequence rather than a cause of splicing defects): We treated 1-cell embryos with the splicing inhibitor pladienolide B (PlaB). Our results show that ZC3H14 nuclear speckles can still assemble at the 2-cell stage under PlaB treatment, although the number of speckles is significantly reduced (new Fig. EV3G). This is a crucial finding, as it demonstrates that the assembly of ZC3H14 speckles is **not merely a downstream consequence of global splicing activity**, but rather an upstream event that can occur even when splicing is inhibited.

To test whether speckle assembly depends on newly synthesized ZGA transcripts: We inhibited global zygotic transcription with α-amanitin at the 1-cell stage (prior to nuclear speckle assembly). ZC3H14 speckles still formed normally at the 2-cell stage (new Fig. EV3E), indicating that ZC3H14-mediated nuclear speckle assembly does not depend on nascent transcripts from ZGA.

Together with our existing evidence—including the loss of speckles in *Zc3h14* KO oocytes (Fig. 5A), the failure of the 4SA phospho-dead mutant to form speckles (Fig. 5E), and the partial rescue by the 4SD phospho-mimetic (Fig. 3F-G)—these new data establish a causal chain in which CDK12/13-mediated phosphorylation drives ZC3H14 speckle assembly, which in turn recruits splicing machinery and ensures proper ZSA. We have restructured the Results and Discussion sections accordingly to present this logic more clearly, while also acknowledging that the relationship between speckle integrity and splicing activity is bidirectional and mutually reinforcing, as discussed in the revised manuscript.

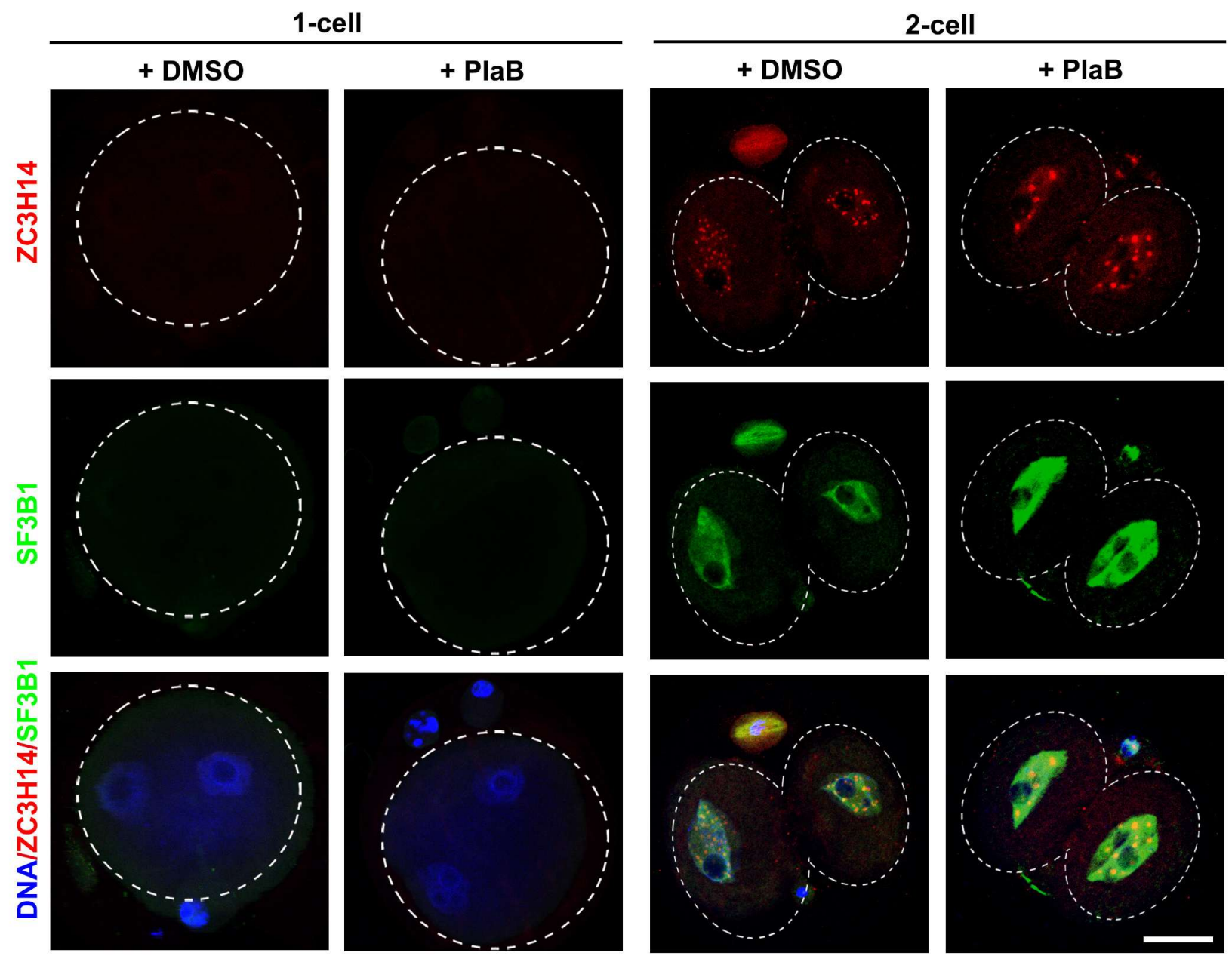

II. Minor Revisions

1. Statistical method standardization: For multi-group comparison experiments (e.g., 3 or more treatment groups in Fig3A, 3F), multiple t-tests should be replaced with one-way analysis of variance (ANOVA) followed by post hoc multiple tests (e.g., Tukey's test). A comprehensive full-text review of the manuscript is recommended.

Response: We thank the reviewer for this suggestion. We have replaced multiple t-tests with one-way or two-way ANOVA followed by post-hoc multiple comparisons tests (Tukey's test) for all multi-group comparisons throughout the revised manuscript, including Fig. 3A, 3F, and other relevant figures. The statistical methods have been updated accordingly in the Materials and Methods section and figure legends.

2. Data presentation standardization: All quantitative plots must clearly indicate the type of error bars (standard error of the mean (SEM)/standard deviation (SD)), and clearly define the meaning of the sample size n: specify whether n is the number of biological replicates (number of mice/independent experiments), technical replicates, or the number of embryos/nuclei counted, and maintain consistency throughout the manuscript.

Response: We have carefully reviewed all quantitative plots in the revised manuscript. Error bars are now clearly indicated as SEM throughout, and the sample size n is explicitly defined in each figure legend—specifying whether n represents the number of embryos analyzed, the number of oocytes, or the number of independent biological replicates (each replicate using embryos from different litters). We have ensured consistency in this presentation across all figures.

3. Abbreviation unification: Unify the abbreviations of proper nouns throughout the manuscript: for example, the abbreviation plaB is given when pladienolide B first appears, and the abbreviation must be consistent throughout the main text and supplementary materials.

Response: We apologize for this oversight. We have now unified all abbreviations throughout the main text and supplementary materials. Specifically, "PlaB" (capitalized consistently) is used for pladienolide B after its first definition, and all other abbreviations have been checked for consistency across the manuscript.

4. Terminology precision correction: "de novo nuclear speckle assembly" is recommended to be revised to "phosphorylation-dependent nuclear speckle reassembly", as nuclear speckles dynamically disassemble and reassemble during the cell cycle, rather than being completely "synthesized de novo"

Response: We fully agree with this important terminological correction. We have revised "*de novo* nuclear speckle assembly" to "**phosphorylation-dependent nuclear speckle reassembly**" throughout the revised manuscript, including the title, abstract, main text, and figure legends. This more accurately reflects the dynamic nature of nuclear speckle formation during the cell cycle and embryonic development.

5. References section correction: Correct the duplicate citation issue (Insco et al, 2023a/b are the same literature and are cited repeatedly). Check the author, year, and journal information of all citations to correct citation errors.

Response: We apologize for these errors. We have corrected the duplicate citation of Insco et al., 2023, and thoroughly checked all references for accuracy of author names, publication years, journal information, and DOI links. All corrections have been made in the revised manuscript.

6. Experimental method details for reproducibility: Supplement the method for the precise volume and concentration of mRNA injection, the specific time point of injection (e.g., specific stage of the pronuclear phase).

Response: We have added the following details to the Materials and Methods section: the injection volume is approximately 5-10 pL, the mRNA concentration is 500-1000 ng/μL, and injections are performed at 20-24 hours post-hCG injection (corresponding to the pronuclear stage). These additions ensure reproducibility of the microinjection experiments.

7. Drug treatment experiment details: Supplement the experimental basis for the selection of drug concentrations of THZ531, α-amanitin, and plaB, the exact time point and duration of treatment. Supplement the detection of transcriptional activity (5-ethynyl uridine (EU) incorporation, RNA polymerase II phosphorylated at serine-2 (pS2) staining) under 5 μM THZ531 treatment to prove that global transcription is not affected at this concentration.

Response: We have now provided detailed information for all drug treatments in the Materials and Methods section. The concentration of THZ531 (1 μM) was chosen based on published studies (Zhang et al., *Nat Chem Biol*, 2016) and our preliminary dose-response assays, which showed significant developmental arrest at 500 nM, 1 μM, and 5 μM, with 1 μM selected to balance efficacy and minimal toxicity. For α-amanitin (25 μg/μl) and PlaB (100 nM), concentrations were selected based on established protocols (Zhang et al., *Adv Sci*, 2024). Zygotes were treated from 22 h post-hCG for 24 hours (through the 1-cell to 2-cell transition). To confirm that 1 μM THZ531 does not affect global transcription, we performed EU incorporation and RNA polymerase II Ser2 phosphorylation (pS2) staining on treated embryos; no significant differences from controls were

observed. We have also included EU and pS2 data under 5 μM THZ531 treatment to further demonstrate that global transcription is unaffected at higher concentrations (new Fig. EV3A-B).

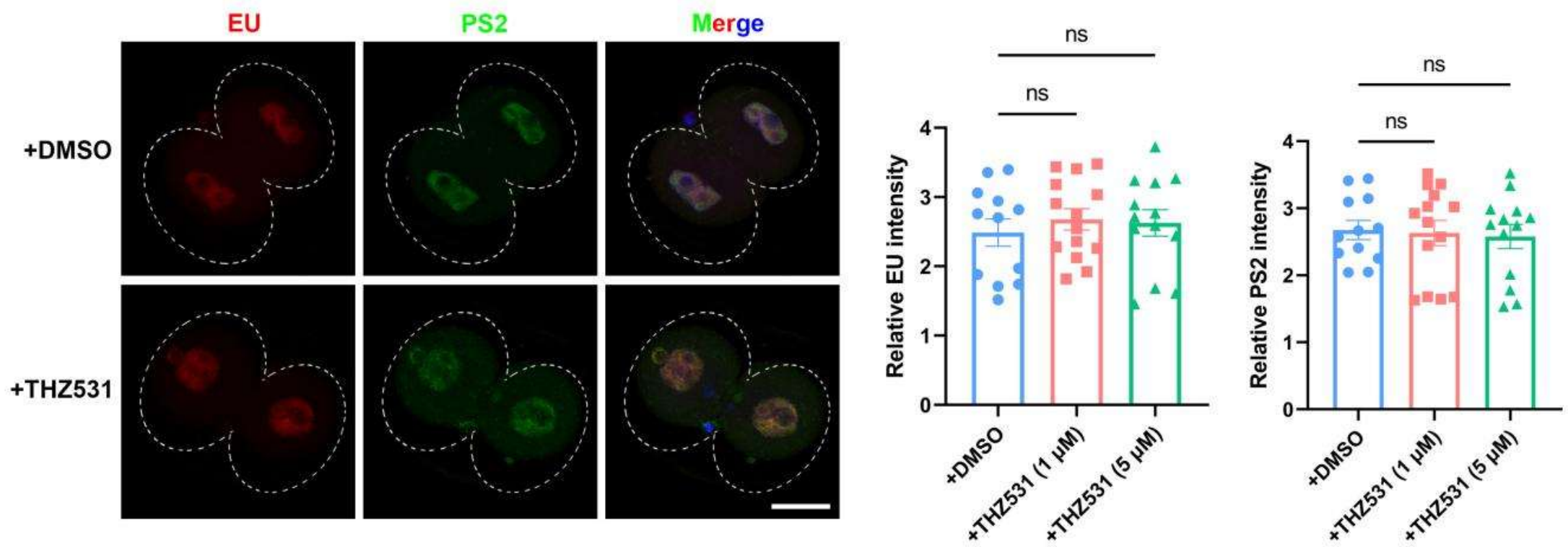

Referee 3:

Major comments

While the study presents a comprehensive ZSA spliced transcriptome dataset related to early embryonic development, its novelty appears limited. As acknowledged by the authors, several aspects of ZC3H14 biology-including its localization to nuclear speckles, involvement in RNA splicing, role in gametogenesis (particularly spermatogenesis), and phosphorylation by CDK13-have been previously reported in other systems. Thus, the scientific impact of the current work remains rather weak.

Response: We sincerely thank the reviewer for the careful reading and for acknowledging the value of our transcriptomic dataset. We fully respect the reviewer's perspective on novelty, and we would like to clarify regarding the unique contributions of our work to the field, particularly in the context of *in vivo* mammalian development.

We acknowledge that certain aspects of ZC3H14 biology—such as its localization to nuclear speckles, involvement in RNA splicing, and phosphorylation by CDK13—have been previously reported, primarily in **heterologous cell line systems** (e.g., HeLa, 293T) or in other biological contexts (e.g., spermatogenesis, cancer models). However, to our knowledge, our study provides the first **physiologically relevant *in vivo* characterization** of ZC3H14 function during a specific, developmentally critical window: zygotic splicing activation (ZSA) in early mammalian embryos. We would like to highlight the following points:

1) From cell lines to *in vivo* development: Prior studies on ZC3H14 nuclear speckle localization and splicing function were largely conducted in immortalized cell lines. Our work demonstrates, for the first time, its **dynamic, stage-specific localization and essential function in a developing mouse embryo *in vivo***, from oocyte to preimplantation stages. The unique physiological demands of ZSA—where the embryo must rapidly and accurately splice a massive wave of newly transcribed zygotic genes—cannot be fully recapitulated in cell line models. This *in vivo* context reveals functional requirements that are simply not observable in heterologous systems.

2) Process-specific novelty (MZT vs. spermatogenesis): We respectfully note that spermatogenesis and the maternal-to-zygotic transition (MZT) are fundamentally distinct developmental processes, with different transcriptional programs, splicing repertoires, and regulatory requirements. The prior report on ZC3H14 in spermatogenesis, while valuable, does not predict or diminish the significance of its essential role in the MZT—a process that governs the very initiation of a new life. The molecular demands and regulatory networks governing these two processes are largely non-overlapping.

3) Mechanistic insight into phosphorylation *in vivo*: We acknowledge that the CDK13-mediated phosphorylation of ZC3H14 was elegantly demonstrated in a previous study (Insco et al., *Science*, 2023), which focused on oncogenic CDK13 mutations in melanoma and its role in nuclear RNA surveillance. However, that study did **not** investigate the developmental function of this phosphorylation event, nor did it establish its essentiality for **nuclear speckle reassembly and ZSA** during normal mammalian embryogenesis. Our study provides the first *in vivo* functional validation of this phosphorylation axis in a developmental context, dissecting *why* phosphorylation matters—that is, how it drives speckle formation, recruits PABPN1, and ensures proper splicing of zygotic transcripts.

We understand that novelty expectations may vary across journals. We are submitting our work to *EMBO reports*, and we believe that a complete, mechanism-driven, physiologically relevant

story—from phenotype to molecular mechanism, supported by multiple state-of-the-art technologies (knockout mice, phospho-mutants, Smart-seq2, and full-length third-generation sequencing)—is well within the scope and standards of the journal. We will, of course, revise the manuscript to more clearly and modestly articulate these points of novelty in the Introduction and Discussion sections, while fully acknowledging the prior foundational work that has enabled our study.

The dataset presented is of potential interest to the field. However, the central claim regarding the requirement of ZC3H14 in ZSA is not sufficiently substantiated. A significant limitation is that many of the key experiments, including transcriptomic analyses, rely on treatment with THZ531. Given that CDK13 has multiple known phosphorylation targets beyond ZC3H14-including components of the translation machinery such as 4E-BP1 and eIF4B, as well as RNA processing factors like RBM39 and ASF/SF2-the observed phenotypes cannot be attributed specifically to ZC3H14 function. Consequently, the immunofluorescence and Smart-seq data derived from THZ531-treated embryos provide only indirect evidence and do not convincingly demonstrate a direct role for ZC3H14 in splicing regulation.

Response: We appreciate the reviewer's concern. We acknowledge that THZ531 is a dual inhibitor of CDK12 and CDK13, and that these kinases have multiple substrates beyond ZC3H14. To address the specificity concern, we have taken the following approaches:

(a) The 4SD phospho-mimetic mutant (where the four serine residues are mutated to aspartic acid) can **partially rescue** the developmental arrest and nuclear speckle defects caused by THZ531 (Fig. 3F-G). This demonstrates that the phosphorylation status of ZC3H14 is a key downstream effector of the inhibitor's action and that at least a significant portion of THZ531's effects on embryonic development are mediated through ZC3H14.

(b) The 4SA phospho-dead mutant phenocopies the effects of THZ531—developmental arrest and loss of speckles—directly showing that preventing ZC3H14 phosphorylation is sufficient to cause the phenotype, independent of any other THZ531 targets.

(c) Based on the reviewer's comment and the acknowledged functional overlap between CDK12 and CDK13, we have **revised the wording throughout the manuscript** to state that **"CDK12/13-mediated phosphorylation of ZC3H14"** is critical for ZSA, rather than attributing it solely to CDK13. This adjustment more accurately reflects the inhibitor specificity and does not affect our core conclusion that ZC3H14 phosphorylation is essential for nuclear speckle reassembly and ZSA.

(d) To provide genetic validation in parallel with pharmacological inhibition, we have performed **CDK13-specific siRNA knockdown experiments** in fertilized eggs (as detailed in our response to Referee 2, Major Concern #2). After knocking down CDK13, ZC3H14 nuclear speckles failed to assemble at the 2-cell stage, and embryo development rates dropped significantly, phenocopying the effects of THZ531 treatment (new Fig. 3H-J and Fig. 4E). These results further support that CDK13 is a major kinase regulating ZC3H14 function during ZSA.

With the phospho-mutant rescue data, the revised wording, and the additional genetic experiments, the specificity concern is adequately addressed while the core conclusion—that ZC3H14 phosphorylation is essential for ZSA—remains firmly supported.

Additionally, the discrepancy between results describing phenotype of *Zc3h14* knockout mice and

the developmental arrest observed upon THZ531 treatment is not adequately addressed. This inconsistency weakens the overall argument and raises concerns about the specificity of the conclusions.

Response: This point is well taken. We have added a clear discussion in the revised manuscript to address this apparent discrepancy. These results are complementary rather than contradictory for the following reasons.

The **systemic *Zc3h14* knockout** leads to a **complete and permanent loss of ZC3H14 protein** throughout oogenesis and early embryogenesis. This results in a fully penetrant maternal-effect phenotype: most embryos arrest during the 1-cell to 2-cell transition, and the remainder arrest at the 2-cell stage, with none progressing to the blastocyst stage (Fig. 2J-K). In contrast, **THZ531 treatment is acute and reversible**, applied to fertilized eggs for a limited 24-hour window. Even at the optimal concentration (1 μM), the inhibitor may not achieve complete and sustained target inhibition in all embryos. Consistent with this interpretation, our dose-response experiments showed that higher concentrations (5 μM) led to more severe effects, while lower concentrations (500 nM) allowed a greater proportion of embryos to escape (data not shown). This concentration-dependent phenotype aligns with the notion that THZ531 produces a partial, reversible inhibition, whereas the knockout creates a permanent, complete loss-of-function state.

Importantly, both interventions converge on the **same core phenotype—developmental arrest at the 2-cell stage**—and both cause **loss of nuclear speckles** and **accumulation of ptRNAs**. The fact that the 4SD phospho-mimetic mutant can partially rescue THZ531-induced arrest (Fig. 3F-G) further confirms that the two phenotypes share a common mechanistic basis: disruption of ZC3H14 phosphorylation. Thus, the observed difference in severity is **quantitative, not qualitative**, and reflects the distinct nature of the two experimental systems rather than any inconsistency in our conclusions.

We have included this analysis in the revised Discussion to preempt any potential misinterpretation.

Several data interpretations require further clarification. For example, in Figure 1D, speckled localization of ZC3H14 is also observed at the 1-cell stage (PN3 and PN5), yet this observation is not sufficiently discussed. This is particularly relevant considering recent studies reporting that embryonic genome activation starts at the 1-cell stage across different mammalian species (e.g., DOI: 10.1073/pnas.2526998123; 10.1016/j.celrep.2023.112023; 10.1016/j.stem.2021.11.012), which are not cited in the manuscript.

Response: We thank the reviewer for this important correction. We agree that the weak speckles observed at the PN3 and PN5 stages are worth discussing, as they coincide with the onset of minor ZGA. We have revised the text to state that **minor, sparse speckle formation can be observed as early as the PN5 stage**, which temporally correlates with the onset of minor ZGA, and that both the number and size of these speckles increase dramatically at the 2-cell stage when major ZGA occurs (revised Fig. 1A). This observation suggests that ZC3H14 speckle assembly begins in parallel with the earliest transcriptional activity of the embryonic genome and scales up with the massive wave of transcription at the 2-cell stage. We have also cited the three recommended papers (Asami et al., *Cell Stem Cell*, 2022; Asami et al., *Cell Rep*, 2023; Jin et al., *PNAS*, 2026) in the revised manuscript to support the concept that ZGA initiates as early as the 1-cell stage. We have added this discussion to the Results section to provide a more complete

description of ZC3H14's dynamic localization during the 1-cell to 2-cell transition.

Additional major points include (italic sentence are quote from the manuscript):

• line 223: *IF results showed that during the transition from the 1-cell to the 2-cell stage, THZ531 treatment led to a reduction or complete disappearance of nuclear speckles in endogenous ZC3H14 cells compared to the control group (Fig. 4E).*

Please add the IF data of the 1-cell stage to support the claim.

Response: We thank the reviewer for this request. We included the IF data for the 1-cell stage in the revised manuscript. As shown in picture below, compared with the DMSO-treated control group, THZ531 treatment led to a reduction of ZC3H14 speckles in the nucleus of 1-cell stage embryos, consistent with the effects observed at the 2-cell stage (Fig. 4F). This confirms that THZ531-mediated inhibition of ZC3H14 phosphorylation begins to affect speckle assembly as early as the 1-cell stage, prior to the major wave of speckle formation at the 2-cell stage. We have added this data and a corresponding description in the Results section of the revised manuscript (new Fig. EV2E).

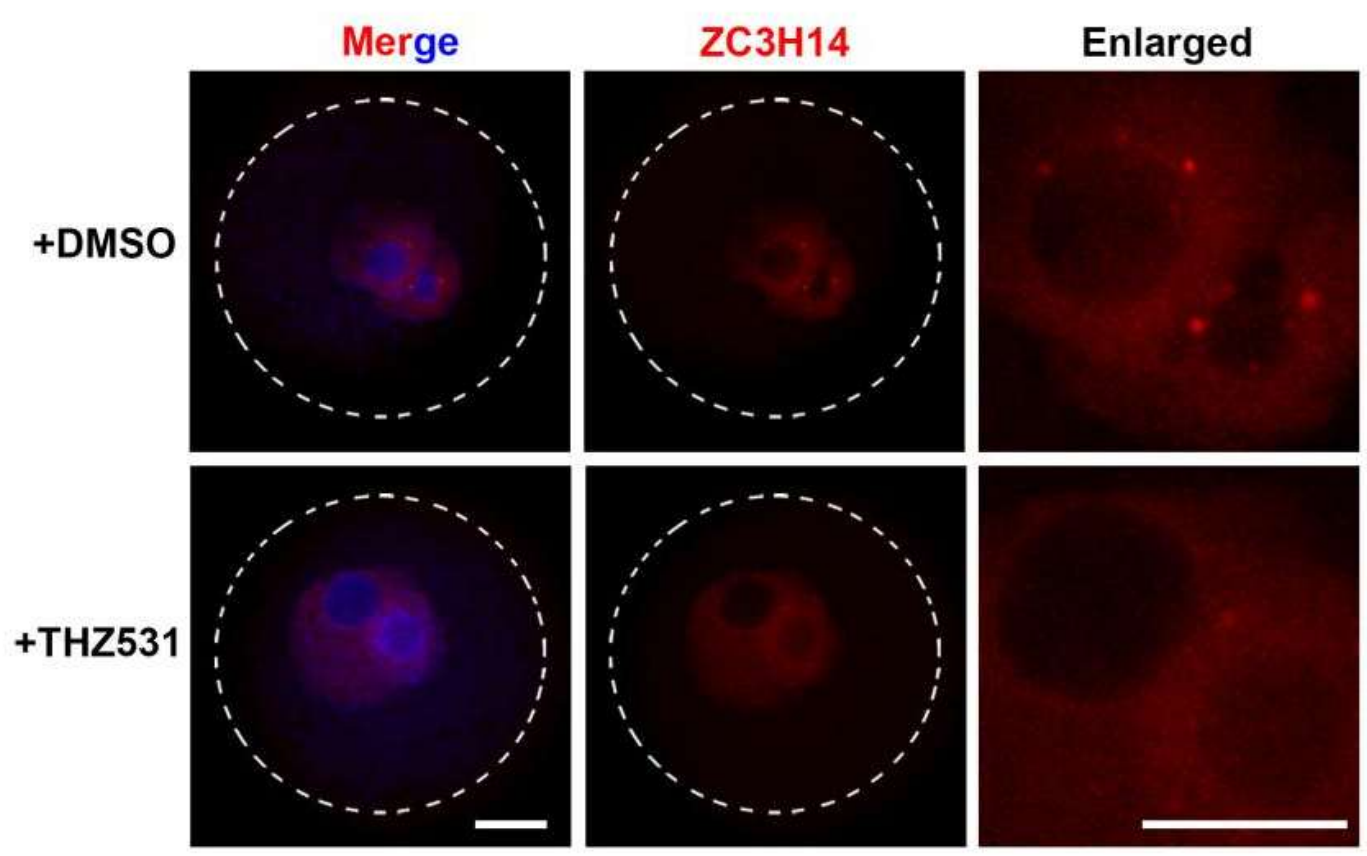

•line 191: *It indicates that ZC3H14 plays an extremely minor role in the maturation of oocytes.*

A recent report by Deng et al. (Nucleic Acid Research, 2026) indicates that ZC3H14 is part of the protein network involved in oocyte maturation, alongside other factors such as TDP43, which is critical for nuclear speckle assembly.

Additionally, multiple previous references indicate the conserved function of ZC3H14 in regulating RNA stability, making its regulation crucial for developmental stages requiring intensive RNA processing, such as oocyte meiotic maturation.

https://doi.org/10.1093/hmg/ddx248

Please explain/discuss the discrepancy of the conclusions.

Response: We thank the reviewer for directing our attention to these important studies. We have carefully read the Deng et al. (NAR, 2026) paper and the additional reference provided.

We do not view our findings as contradictory to these reports, but rather as complementary at different levels of analysis. The Deng et al. study identified ZC3H14 as a component of a protein interaction network in oocytes, which is valuable for understanding the molecular landscape of oocyte maturation. However, this study did not perform functional validation—such as knockout or inhibition—to assess whether ZC3H14 is *essential* for the maturation process. Network predictions

can reveal potential interactions, but they do not establish functional necessity, as genetic redundancy or compensatory mechanisms may mask the phenotypic impact of individual components.

In contrast, our study provides **direct functional evidence**: THZ531 treatment (which inhibits ZC3H14 phosphorylation via CDK12/13) and the 4SA phospho-dead mutant both showed no significant effect on GVBD or PBE rates (Fig. 3D-E). These data demonstrate that ZC3H14 phosphorylation is **not essential** for the meiotic progression of fully-grown oocytes under our experimental conditions. It remains possible that ZC3H14 participates in the oocyte maturation network in a redundant manner alongside other RNA-binding proteins (e.g., ZC3H13, TDP43), consistent with the network prediction, but that its individual loss or inhibition is compensated.

We have now cited the Deng et al. study and the additional reference in the revised Discussion, and we have objectively discussed this distinction between network involvement and functional essentiality. We have revised our conclusion to more precisely state that **ZC3H14 is not essential for oocyte meiotic maturation under our experimental conditions**, while acknowledging that it may participate in the broader molecular network in a redundant capacity.

• line 262: *Principal component analysis (PCA) showed no significant differences between the experimental and control 1-cell embryos, whereas the 2-cell embryos showed clear distinctions (Fig. 6A).*

Fig 6 A: PCA shows a clear difference in clustering between control and experimental 1-cell embryos. Please revise the l.262 statement and expand the discussion to explain this.

Response: We thank the reviewer for this careful observation. Upon re-examining the PCA plot, we agree that there is indeed some separation between THZ531-treated and control samples as early as the 1-cell stage, although the separation is much less pronounced than at the 2-cell stage. We have revised the text accordingly to state: **"Principal component analysis revealed some separation between THZ531-treated and control embryos at the 1-cell stage; however, the transcriptional differences became much more pronounced and robust at the 2-cell stage (Fig. 6A)."**

We have also expanded the discussion to address potential reasons for this early separation. THZ531 was added at the early 1-cell stage (22 h post-hCG), and it is plausible that the inhibitor exerts a mild effect on the minor wave of zygotic transcription that occurs at the 1-cell stage, or alternatively, that it affects the stability of maternal transcripts that are being actively degraded during this period. However, the major transcriptional and splicing defects are clearly manifested at the 2-cell stage, coinciding with major ZGA when the demand on the splicing machinery is at its peak. We have added this discussion to the revised manuscript to provide a more complete interpretation of the PCA results.

• line 339: *To study the effects of ptRNA accumulation on early mouse embryonic development, we constructed Ddx39b-ptRNA with the last four exons deleted at the 3' end and overexpressed it in mouse zygotes via microinjection. The control group was microinjected with GFP mRNA.*

What would be the developmental effect of full-length Ddx39b-RNA?

Response: We thank the reviewer for this important control question. As shown in new Fig. 7J-K (included in the response document), we performed the requested control experiment: overexpression of full-length *Ddx39b* (Ddx39b-FL) in mouse zygotes had **no significant effect** on

embryo development, with embryos progressing normally to the blastocyst stage at rates comparable to the GFP-injected control group. In striking contrast, overexpression of the Ddx39b-ptRNA construct (lacking the last four exons) caused significant 2-cell arrest, phenocopying the effect of THZ531 treatment. This control confirms that the developmental defect is specifically attributable to the accumulation of the truncated ptRNA transcript rather than to any non-specific effect of Ddx39b overexpression. We have added this control data to the revised figures (Fig. 7J-K) and included a corresponding description in the Results section.

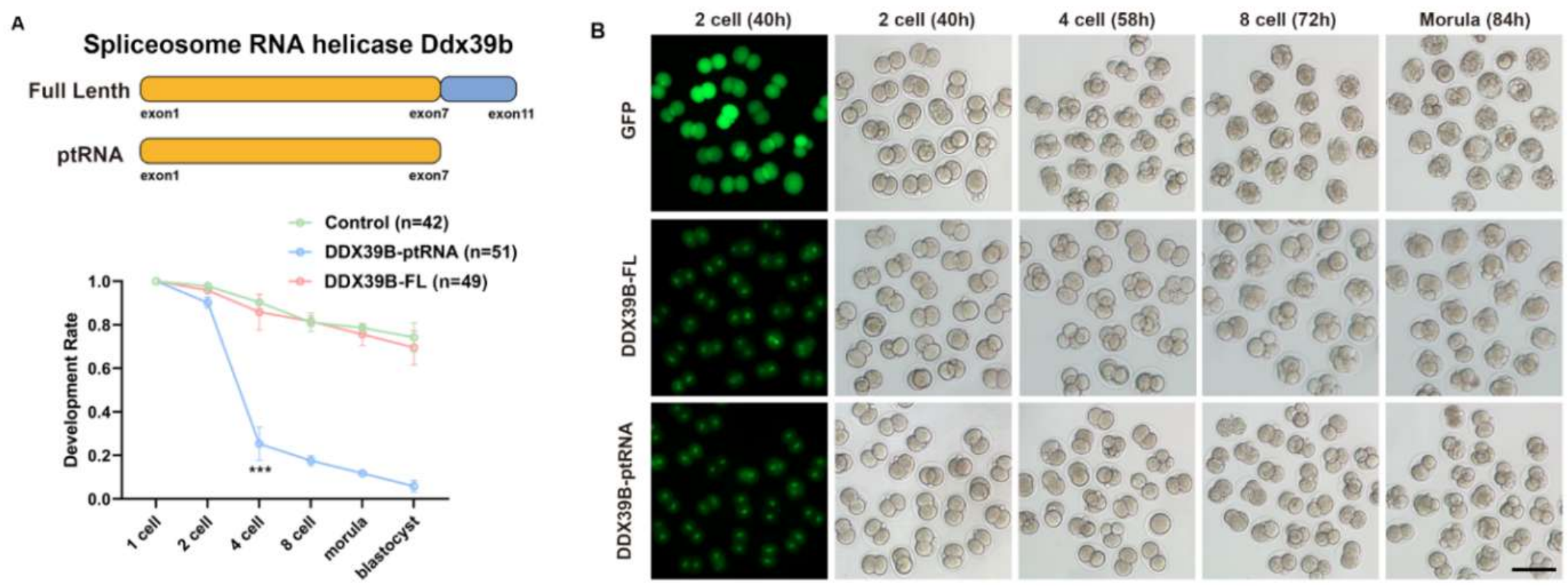

Minor comments

Fig S1B scale bar is missing

Response: We apologize for this oversight. We have now added the scale bar to Fig. EV1E (previously Fig. S1B) in the revised manuscript.

Fig S2 legend says immunofluorescence staining, however the main text says "*.....overexpression of GFP-ZC3H14 in oocytes and early embryos confirmed these observations (Fig. S2).*" Please clarify what it is that is being referred to in each and how they relate to each other.

Response: We apologize for the inaccurate description. We have corrected the legend and text in the revised manuscript to clarify the distinction: **Fig. 1A** (previously Fig. 1D) shows immunofluorescence staining of **endogenous ZC3H14** using a ZC3H14-specific antibody at different developmental stages, while **Fig. EV1G** (previously Fig. S2) shows **GFP-ZC3H14 overexpression** experiments in oocytes and early embryos, which confirm the same stage-specific localization pattern observed for the endogenous protein. The two figures are complementary: the endogenous staining establishes the native localization dynamics, while the overexpression experiment validates these observations with an independently tagged construct.

Dear Dr. Fan,

Thank you for the submission of your revised manuscript. We have now received the enclosed reports from the referees that were asked to assess it and I am happy to say that both support its publication now. Referee 3 has some more suggestions that I would like you to address in the final ms version.

Also a few editorial requests will need to be addressed before we can proceed with the official acceptance of your manuscript:

- Please remove all figures from the ms file and place the legends at the end of the ms file. All main and EV figures need to be uploaded as individual, high quality production files.

- Please place the Data Availability Statement before the Acknowledgments.

- The conflict of interest subheading needs to be corrected to "Disclosure and Competing Interests Statement"

- The co-corr. author Li-Ling Liu needs an institutional email address.

- The author credits need to be removed from the ms file. All credits need to be entered during online ms submission.

- The separate Funding section should be removed from the ms file as this info goes under Acknowledgments.

- The separate highlighted Word file with EV figures, legends and EV tables needs to be deleted.

- "Supplementary Table EV3/4/6/7" is not a correct callout. Table EV1-Table EV5 are datasets and need to be updated to Dataset EV1-Dataset EV5 in all places; Table EV6 and Table EV7 should be removed from the ms and uploaded separately as Expanded View Tables; the legends of all these tables need to be removed from the ms and each legend should be part of its (Excel) table file (as a separate sheet/tab in the Excel file).

- The Materials and Methods section should include a separate Reagents and Tools Table file (listing key reagents, experimental models, software and relevant equipment and including their sources and relevant identifiers) and a Methods and Protocols section in which methods should be described using a step-by-step protocol format with bullet points. More information and a downloadable Table template is available in our guide to authors online: https://link.springer.com/journal/44319/submission-guidelines

- Materials and Methods should be Methods.

Our routine image analysis detected 2 possible figure panel reuses:

- Cell reuse between Figure 1A and Figure 4F, not in the legend.
- Cell reuse between Figure 5B and Figure 7K, not in the legend.

Can you please clarify what happened.

* Figure Legends - Comments *

- Please note that the exact p values are not provided in the legends of figures 1C, 2C, D, F, J; 3A, F, I; 4D, H; 5C, J; 6K, 7A, H; EV2 D, EV3 D, EV5B. Please provide exact values as reasonable.
- Please indicate the statistical test used for data analysis in the legends of figures 6B, D; 7D, F; EV5 E
- Please note that the box plots need to be defined in terms of minima, maxima, centre, bounds of box and whiskers, and percentile in the legend of figure 7H
- Please note that information related to n is missing in the legends of figures 6K, 7A, C, H; EV1 B.
- Please note that the error bars are not defined in the legends of figures 1B, 7C, EV3 B, D, F.
- Please note that the white dashed borders are not defined in the legend of figure 1A, B, C, D, E; 4A, B, E, F; 5A, B, E; EV1 G, EV2 E, EV3 A, C, E, G; EV4 A-C. This needs to be rectified.

I would like to suggest some minor changes to the title and abstract that needs to be written in present tense. Do you agree with this :

ZC3H14 Phosphorylation is Required for Nuclear Speckle Assembly During Zygotic Splicing Activation in Mice

Following zygotic genome activation (ZGA) in embryos, nuclear speckles, which are subnuclear domains where pre-mRNA undergoes splicing, are reassembled in a process called zygotic splicing activation (ZSA). ZSA is vital for early development, but its regulation is poorly understood. This study identifies the RNA-binding protein ZC3H14 as a key promoter of nuclear speckle formation during ZSA. Although viable, Zc3h14 knockout mice exhibit reduced female fertility. Most embryos from these females arrest during the 1-cell to 2-cell transition, and the remaining embryos arrest at the 2-cell stage, highlighting a critical role for maternally deposited ZC3H14. The protein localizes to nuclear speckles, and its phosphorylation by CDK12/13 regulates mRNA binding and initial speckles assembly. Blocking this phosphorylation disrupts proper splicing and leads to abnormal RNA accumulation. Consequently, phosphorylation-mimicking or phosphorylation-blocking ZC3H14 mutants expressed in zygotes positively and negatively affect development, respectively. Taken together, our results indicate that ZC3H14 phosphorylation by CDK12/13 maintains fertility in female mice by promoting nuclear speckle formation during ZSA.

EMBO press papers are accompanied online by A) a short (1-2 sentences) summary of the findings and their significance, B) 2-3 bullet points highlighting key results and C) a synopsis image that is exactly 550 pixels wide and 300 pixels high. The synopsis image should provide a sketch of the major findings, like a graphical abstract. Please note that text needs to be readable at the final size. Please send us this information along with the final manuscript.

I look forward to seeing a final version of your manuscript as soon as possible.

Kind regards,
Esther

Esther Schnapp, PhD
Senior Editor
EMBO reports

Referee #1:

The ms is suitable for publication in EMBO reports without revision

Referee #3:

Thank you to the authors for revising the manuscript and for providing a detailed response to my previous comments. I appreciate the considerable effort made to address the concerns raised during the initial review, and the manuscript has improved as a result.

Most of my previous comments have been satisfactorily addressed. After carefully reviewing the revised manuscript and the authors' responses, I have a few additional suggestions that I hope the authors will find helpful in further strengthening the manuscript:

1. Materials and Methods - Mice
The authors state that Zc3h14−/− mice were generated using CRISPR-Cas9-based gene targeting and maintained on a C57BL/6J genetic background. For clarity and reproducibility, it would be helpful to provide some additional methodological details regarding the generation and validation of the knockout mice, where appropriate. This could include information on the gRNA sequence(s), the targeted genomic region and mutation introduced, the developmental stage at which genome editing was performed, and the strategy used to confirm the genotype and knockout status of the animals.
It may also be valuable to briefly expand the discussion by comparing the Zc3h14−/− mouse model used in this study with previously reported Zc3h14 knockout models, highlighting any relevant similarities or differences.

2. Materials and Methods - Quantitative RT-PCR
The authors state that total RNA was extracted from oocytes using the RNeasy Mini Kit. For additional clarity, it would be helpful to indicate the approximate number of oocytes used per sample for RNA extraction.

3. Terminology regarding genome activation
I would like to suggest that the authors consider whether the terminology used in the statement, "Major ZGA usually occurs at the 2-cell stage," could be further refined. At this developmental stage, following the first cleavage division, the term embryonic genome activation (EGA) may be morphologically and semantically more appropriate, as the concept extends beyond the single-cell zygote stage. While transcription may initiate as an immediate or minor wave in the one-cell embryo/zygote, the major wave occurs after cleavage. Therefore, the use of "major EGA" might provide terminology that more broadly and accurately encompasses genome activation across these developmental stages.

I appreciate that both "major ZGA" and "major EGA" are used in the literature, and the authors may wish to consider this distinction in light of recent studies that use these terms to describe the higher-amplitude transcriptional wave in multicellular embryos (doi: 10.3389/fcell.2025.1594995).

4. Consistency between statements and cited references
Relatedly, the statement "Major ZGA usually occurs at the 2-cell stage" appears to be most directly applicable to the mouse model. However, several references cited in this section concern human and/or bovine embryos, in which the major wave of embryonic genome activation occurs at later developmental stages, approximately the 4-8-cell stage, following the earlier initiation of transcription at the one-cell stage.
To avoid any potential ambiguity, the authors may wish to revise or expand this paragraph to acknowledge these species-specific differences in the timing of genome activation and to ensure that the text is closely aligned with the scope of the cited literature.

Overall, I appreciate the authors' careful revision and thoughtful responses to the previous comments. I hope that these additional suggestions will be helpful in further improving the clarity and scientific context of the manuscript.

Dear Dr. Schnapp,

Thank you for your email regarding the final preparation of our manuscript (EMBOR-2026-63958V2). We are very grateful to you and the Referees for the positive evaluation and for the detailed editorial guidance.

We have now carefully addressed all the requests and have responded to and revised the manuscript in light of the additional suggestions fromReferee 3 point by point. The revised manuscript materials have been prepared and uploaded via the online system. Below is a summary of the main changes and clarifications.

1. Clarification on figure panel reuse

We sincerely apologize for the two instances of suspected figure panel reuse. Upon thorough re-examination of our original data:

Figure 1A vs. Figure 4F: We confirmed that these two panels were inadvertently miss-selected during figure compilation. The images were derived from separate experiments, but due to the large number of immunofluorescence images generated in this study, an incorrect image was mistakenly placed in Figure 4F. We have now retrieved the correct original image for Figure 4F and have replaced it in the revised manuscript.

Figure 5D vs. Figure 7K: Similarly, these two images originated from independent experiments and were incorrectly duplicated during assembly. We have located the correct original image for Figure 5D and have replaced it accordingly.

All corresponding Source Data files have been updated with the correct images. We have double checked all other figure panels to ensure no further miss-assignments exist.

2. Clarification on the co-localization fluorescence intensity profiles in Figure 1B–E

Regarding your request to define error bars and provide exact P-values in the legends of Figures 1B and 1C, we would like to clarify the following:

The curves shown in **Figures 1B, 1C, 1D, and 1E** are not quantitative statistical comparison plots. Rather, they are fluorescence intensity profiles used to qualitatively assess the fluorescence intensity changes of ZC3H14 and different proteins (SRRM2, SRSF2, PABPN1, and Poly(A)) at the same position, thereby evaluating their co-localization. These fitted curves represent qualitative analyses and do not involve quantitative statistical comparisons between multiple groups; therefore, they do not contain error bars or P-values, which is standard practice for co-localization analysis. We have clarified this in the figure legend for this set of panels.

3. Responses to Referee 3's additional suggestions

Referee 3 raised four additional suggestions in the second round of review, all of which we have fully addressed:

(1)Mouse model details: We have supplemented the Methods section with detailed information on the mouse model used in this study.

(2)RTqPCR oocyte numbers: We have added to the Methods section the specific information that approximately 10 oocytes or embryos were used per RNA sample.

(3) ZGA/EGA terminology: Given that our study focuses exclusively on the mouse model, where the term "ZGA" is well-established and widely used in the field, we prefer to retain "major ZGA" in the main text. However , at the first appearance of ZGAin the Introduction, we have added the clarifying phrase "also referred to as embryonic genome activation (EGA) in some contexts."

(4)Species differences and citation consistency: In the Introduction, we have added a statement clarifying the species specific timing of major ZGA/EGA, explicitly indicating that it occurs at the 2 cell stage in mice and at the 4 to 8 cell stage in humans and cattle, ensuring that the text is fully consistent with the cited literature.

4. All other editorial requests have been addressed

We have carefully followed each of your instructions:

- Figures removed from the manuscript file; main and EV figures uploaded as individual high-resolution production files.
- Figure legends placed at the end of the manuscript file.
- Data Availability Statement moved before the Acknowledgements.
- Conflict of interest heading corrected to "Disclosure and Competing Interests Statement".
- The co-corr. author Li-Ling Liu replaced an institutional email address.
- Author contributions and separate Funding section removed from the manuscript file.
- Expanded View tables removed as requested.
- Materials and Methods section updated (Reagents and Tools Table uploaded; "Materials and Methods" changed to "Methods").
- Exact P-values added to all relevant figures and indicated in the corresponding legends; statistical tests and n clearly defined; box plot definitions and white dashed border annotations added.
- Title and abstract revised to present tense as you suggested (we fully agree with your proposed version).
- Synopsis image and bullet points prepared and uploaded.

We believe the revised manuscript now fully meets the journal's requirements. We are grateful for your patience and editorial guidance throughout this process, and we hope that the revised version is now acceptable for publication.

Please let us know if any further adjustments are needed.

With kind regards,

Yang Wang, Ph.D

Life Sciences Institute, Zhejiang University,

Hangzhou, China

Dr. Heng-Yu Fan
Zhejiang University
Life Sciences Institute
866 Yu Hang Tang Rd
Hangzhou 310058
China

Dear Dr. Fan,

My colleague Esther Schnapp is currently travelling and I have therefore taken over the handling of your manuscript. I checked all files and revisions and am very pleased to accept your manuscript for publication in the next available issue of EMBO reports. Thank you for your contribution to our journal.

Your manuscript will be processed for publication by EMBO Press. It will be copy edited and you will receive page proofs prior to publication. Please note that you will be contacted by Springer Nature Author Services to complete licensing and payment information.

You may qualify for financial assistance for your publication charges - either via a Springer Nature fully open access agreement or an EMBO initiative. Check your eligibility: https://link.springer.com/journal/44319/how-to-publish-with-us

Should you be planning a Press Release on your article, please notify the production team via this form in order to coordinate publication and release dates: https://forms.cloud.microsoft/e/eH4A8JVP6g

If you have any questions, please do not hesitate to contact the Editorial Office. Thank you for your contribution to EMBO Reports.

Yours sincerely,

Martina Rembold, PhD
Senior Editor
EMBO reports

>>> Please note that it is EMBO Reports policy for the transcript of the editorial process (containing referee reports and your response letter) to be published as an online supplement to each paper. If you do NOT want this, you will need to inform the Editorial Office via email immediately. More information is available here: https://link.springer.com/partners/embo-press/editorial-policies#Peer%20review

EMBO Press Author Checklist

Corresponding Author Name: Heng-Yu Fan	
Journal Submitted to: EMBO Reports	
Manuscript Number: EMBOR-2026-63958V1	

USEFUL LINKS FOR COMPLETING THIS FORM
The EMBO Journal - Author Guidelines
EMBO Reports - Author Guidelines
Molecular Systems Biology - Author Guidelines
EMBO Molecular Medicine - Author Guidelines

Reporting Checklist for Life Science Articles (updated January
This checklist is adapted from Materials Design Analysis Reporting (MDAR) Checklist for Authors. MDAR establishes a minimum set of requirements in transparent reporting in the life sciences (see Statement of Task: 10.31222/osf.io/9sm4x). Please follow the journal's guidelines in preparing your
Please note that a copy of this checklist will be published alongside your article.

Abridged guidelines for figures
1. Data
The data shown in figures should satisfy the following conditions:
- ➞ the data were obtained and processed according to the field's best practice and are presented to reflect the results of the experiments in an accurate and unbiased manner.
- ➞ ideally, figure panels should include only measurements that are directly comparable to each other and obtained with the same assay.
- ➞ plots include clearly labeled error bars for independent experiments and sample sizes. Unless justified, error bars should not be shown for technical
- ➞ if n<5, the individual data points from each experiment should be plotted. Any statistical test employed should be justified.
- ➞ Source Data should be included to report the data underlying figures according to the guidelines set out in the authorship guidelines on Data

2. Captions
Each figure caption should contain the following information, for each panel where they are relevant:
- ➞ a specification of the experimental system investigated (eg cell line, species name).
- ➞ the assay(s) and method(s) used to carry out the reported observations and measurements.
- ➞ an explicit mention of the biological and chemical entity(ies) that are being measured.
- ➞ an explicit mention of the biological and chemical entity(ies) that are altered/varied/perturbed in a controlled manner.
- ➞ the exact sample size (n) for each experimental group/condition, given as a number, not a range;
- ➞ a description of the sample collection allowing the reader to understand whether the samples represent technical or biological replicates (including how many animals, litters, cultures, etc.).
- ➞ a statement of how many times the experiment shown was independently replicated in the laboratory.
- ➞ definitions of statistical methods and measures:
  - common tests, such as t-test (please specify whether paired vs. unpaired), simple χ2 tests, Wilcoxon and Mann-Whitney tests, can be unambiguously identified by name only, but more complex techniques should be described in the methods section;
  - are tests one-sided or two-sided?
  - are there adjustments for multiple comparisons?
  - exact statistical test results, e.g., P values = x but not P values < x;
  - definition of 'center values' as median or average;
  - definition of error bars as s.d. or s.e.m.

Please complete ALL of the questions below.
Select "Not Applicable" only when the requested information is not relevant for your study.

Materials

Newly Created Materials	Information included in the manuscript?	In which section is the information available? (Reagents and Tools Table, Materials and Methods, Figures, Data Availability Section)
New materials and reagents need to be available; do any restrictions apply?	Yes	All plasmids and reagents can be obtained from the corresponding author or through commercial channels.

Antibodies	Information included in the manuscript?	In which section is the information available? (Reagents and Tools Table, Materials and Methods, Figures, Data Availability Section)
For **antibodies** provide the following information:<br>- Commercial antibodies: RRID (if possible) or supplier name, catalogue number and or/clone number<br>- Non-commercial: RRID or citation	Yes	Reagents and Tools Table

DNA and RNA sequences	Information included in the manuscript?	In which section is the information available? (Reagents and Tools Table, Materials and Methods, Figures, Data Availability Section)
**Short novel DNA or RNA including primers, probes**: provide the sequences.	Yes	Reagents and Tools Table

Cell materials	Information included in the manuscript?	In which section is the information available? (Reagents and Tools Table, Materials and Methods, Figures, Data Availability Section)
**Cell lines:** Provide species information, strain. Provide accession number in repository **OR** supplier name, catalog number, clone number, and/**OR** RRID.	Yes	Materials and Methods
**Primary cultures:** Provide species, strain, sex of origin, genetic modification status.	Yes	Materials and Methods
Report if the cell lines were recently **authenticated** (e.g., by STR profiling) and tested for mycoplasma contamination.	Yes	Materials and Methods

Experimental animals	Information included in the manuscript?	In which section is the information available? (Reagents and Tools Table, Materials and Methods, Figures, Data Availability Section)
**Laboratory animals or Model organisms:** Provide species, strain, sex, age, genetic modification status. Provide accession number in repository **OR** supplier name, catalog number, clone number, **OR** RRID.	Yes	Materials and Methods
**Animal observed in or captured from the field:** Provide species, sex, and age where possible.	Not Applicable	
Please detail **housing and husbandry conditions**.	Yes	Materials and Methods

Plants and microbes	Information included in the manuscript?	In which section is the information available? (Reagents and Tools Table, Materials and Methods, Figures, Data Availability Section)
**Plants:** provide species and strain, ecotype and cultivar where relevant, unique accession number if available, and source (including location for collected wild specimens).	Not Applicable	
**Microbes:** provide species and strain, unique accession number if available, and source.	Not Applicable	

Human research participants	Information included in the manuscript?	In which section is the information available? (Reagents and Tools Table, Materials and Methods, Figures, Data Availability Section)
If collected and within the bounds of privacy constraints report on age, sex and gender or ethnicity for all study participants.	Not Applicable	

Core facilities	Information included in the manuscript?	In which section is the information available? (Reagents and Tools Table, Materials and Methods, Figures, Data Availability Section)
If your work benefited from core facilities, was their service mentioned in the acknowledgments section?	Yes	Materials and Methods

Design

**Study protocol**	**Information included in the manuscript?**	**In which section is the information available?** (Reagents and Tools Table, Materials and Methods, Figures, Data Availability Section)
If study protocol has been **pre-registered, provide DOI in the manuscript**. For clinical trials, provide the trial registration number **OR** cite DOI.	Not Applicable	
Report the **clinical trial registration number** (at ClinicalTrials.gov or equivalent), where applicable.	Not Applicable	

**Laboratory protocol**	**Information included in the manuscript?**	**In which section is the information available?** (Reagents and Tools Table, Materials and Methods, Figures, Data Availability Section)
Provide DOI OR other citation details if **external detailed step-by-step protocols** are available.	Yes	Materials and Methods

**Experimental study design and statistics**	**Information included in the manuscript?**	**In which section is the information available?** (Reagents and Tools Table, Materials and Methods, Figures, Data Availability Section)
Include a statement about **sample size** estimate even if no statistical methods were used.	Yes	Materials and Methods, Figures
Were any steps taken to minimize the effects of subjective bias when allocating animals/samples to treatment (e.g. **randomization procedure**)? If yes, have they been described?	Yes	Materials and Methods
Include a statement about **blinding** even if no blinding was done.	Yes	Materials and Methods
Describe **inclusion/exclusion criteria** if samples or animals were excluded from the analysis. Were the criteria pre-established?<br><br>If sample or data points were omitted from analysis, report if this was due to attrition or intentional exclusion and provide justification.	Yes	Materials and Methods
For every figure, are **statistical tests** justified as appropriate? Do the data meet the assumptions of the tests (e.g., normal distribution)? Describe any methods used to assess it. Is there an estimate of variation within each group of data? Is the variance similar between the groups that are being statistically compared?	Yes	Materials and Methods, Figures

**Sample definition and in-laboratory replication**	**Information included in the manuscript?**	**In which section is the information available?** (Reagents and Tools Table, Materials and Methods, Figures, Data Availability Section)
In the figure legends: state number of times the experiment was **replicated** in laboratory.	Yes	Figures
In the figure legends: define whether data describe **technical or biological replicates**.	Yes	Materials and Methods, Figures

Ethics

**Ethics**	**Information included in the manuscript?**	**In which section is the information available?** (Reagents and Tools Table, Materials and Methods, Figures, Data Availability Section)
Studies involving **human participants**: State details of **authority granting ethics approval** (IRB or equivalent committee(s), provide reference number for approval.	Not Applicable	
Studies involving **human participants**: Include a statement confirming that **informed consent** was obtained from all subjects and that the experiments conformed to the principles set out in the WMA Declaration of Helsinki and the Department of Health and Human Services Belmont Report.	Not Applicable	
Studies involving **human participants:** For publication of **patient photos**, include a statement confirming that consent to publish was obtained.	Not Applicable	
Studies involving experimental **animals**: State details of **authority granting ethics approval** (IRB or equivalent committee(s), provide reference number for approval. Include a statement of compliance with ethical regulations.	Yes	Materials and Methods
Studies involving **specimen and field samples:** State if relevant **permits** obtained, provide details of authority approving study; if none were required, explain why.	Not Applicable	

**Dual Use Research of Concern (DURC)**	**Information included in the manuscript?**	**In which section is the information available?** (Reagents and Tools Table, Materials and Methods, Figures, Data Availability Section)
Could your study fall under dual use research restrictions? Please check biosecurity documents and list of **select agents and toxins** (CDC): https://www.selectagents.gov/sat/list.htm	Not Applicable	
If you used a select agent, is the security level of the lab appropriate and reported in the manuscript?	Not Applicable	
If a study is subject to dual use research of concern regulations, is the name of the **authority granting approval and reference number** for the regulatory approval provided in the manuscript?	Not Applicable	

Reporting

The MDAR framework recommends adoption of discipline-specific guidelines, established and endorsed through community initiatives. Journals have their own policy about requiring specific guidelines and recommendations to complement MDAR.

**Adherence to community standards**	**Information included in the manuscript?**	**In which section is the information available?** (Reagents and Tools Table, Materials and Methods, Figures, Data Availability Section)
State if relevant guidelines or checklists (e.g., **ICMJE, MIBBI, ARRIVE, PRISMA**) have been followed or provided.	Not Applicable	
For **tumor marker prognostic studies,** we recommend that you follow the **REMARK** reporting guidelines (see link list at top right). See author guidelines, under 'Reporting Guidelines'. Please confirm you have followed these guidelines.	Not Applicable	
For **phase II and III randomized controlled trials**, please refer to the **CONSORT** flow diagram (see link list at top right) and submit the CONSORT checklist (see link list at top right) with your submission. See author guidelines, under 'Reporting Guidelines'. Please confirm you have submitted this list.	Not Applicable	

Data Availability

**Data availability**	**Information included in the manuscript?**	**In which section is the information available?** (Reagents and Tools Table, Materials and Methods, Figures, Data Availability Section)
Have **primary datasets** been deposited according to the journal's guidelines (see 'Data Deposition' section) and the respective accession numbers provided in the Data Availability Section?	Yes	Data Availability
Were **human clinical and genomic datasets** deposited in a public access-controlled repository in accordance to ethical obligations to the patients and to the applicable consent agreement?	Not Applicable	
Are **computational models** that are central and integral to a study available without restrictions in a machine-readable form? Were the relevant accession numbers or links provided?	Not Applicable	
If publicly available data were reused, provide the respective **data citations in the reference list**.	Yes	Data Availability
